# Multiomics analysis reveals the mechanical stress-dependent changes in trabecular meshwork cytoskeletal-extracellular matrix interactions

**DOI:** 10.3389/fcell.2022.874828

**Published:** 2022-09-13

**Authors:** Avinash Soundararajan, Ting Wang, Rekha Sundararajan, Aruna Wijeratne, Amber Mosley, Faith Christine Harvey, Sanjoy Bhattacharya, Padmanabhan Paranji Pattabiraman

**Affiliations:** ^1^ Department of Ophthalmology, Glick Eye Institute, Indiana University School of Medicine, Indianapolis, IN, United States; ^2^ Stark Neuroscience Research Institute, Indiana University School of Medicine, Indianapolis, IN, United States; ^3^ Department of Biochemistry and Molecular Biology, Indiana University School of Medicine, Indianapolis, IN, United States; ^4^ Center for Proteome Analysis, Indiana University School of Medicine, Indianapolis, IN, United States; ^5^ Center for Computational Biology and Bioinformatics, Indiana University School of Medicine, Indianapolis, IN, United States; ^6^ Bascom Palmer Eye Institute, Miller School of Medicine at University of Miami, Miami, FL, United States; ^7^ Miami Integrative Metabolomics Research Center, Miami, FL, United States

**Keywords:** trabecular meshwork (TM), glaucoma, ocular hypertension, mechanical stretch, multiomics analysis, cytoskeleton, extracellular matrix, lipid signaling

## Abstract

Trabecular meshwork (TM) tissue is subjected to constant mechanical stress due to the ocular pulse created by the cardiac cycle. This brings about alterations in the membrane lipids and associated cell–cell adhesion and cell–extracellular matrix (ECM) interactions, triggering intracellular signaling responses to counter mechanical insults. A loss of such response can lead to elevated intraocular pressure (IOP), a major risk factor for primary open-angle glaucoma. This study is aimed to understand the changes in signaling responses by TM subjected to mechanical stretch. We utilized multiomics to perform an unbiased mRNA sequencing to identify changes in transcripts, mass spectrometry- (MS-) based quantitative proteomics for protein changes, and multiple reaction monitoring (MRM) profiling-based MS and high-performance liquid chromatography (HPLC-) based MS to characterize the lipid changes. We performed pathway analysis to obtain an integrated map of TM response to mechanical stretch. The human TM cells subjected to mechanical stretch demonstrated an upregulation of protein quality control, oxidative damage response, pro-autophagic signal, induction of anti-apoptotic, and survival signaling. We propose that mechanical stretch-induced lipid signaling *via* increased ceramide and sphingomyelin potentially contributes to increased TM stiffness through actin-cytoskeleton reorganization and profibrotic response. Interestingly, increased phospholipids and diacylglycerol due to mechanical stretch potentially enable cell membrane remodeling and changes in signaling pathways to alter cellular contractility. Overall, we propose the mechanistic interplay of macromolecules to bring about a concerted cellular response in TM cells to achieve mechanotransduction and IOP regulation when TM cells undergo mechanical stretch.

## Introduction

Glaucoma is an aging disease that causes irreversible blindness worldwide, affecting ∼80 million people across the globe (Tham et al., 2014; Susanna et al., 2015). Primary open-angle glaucoma (POAG) is the most common form of glaucoma, affecting over 57.5 million people worldwide (Allison et al., 2020). Elevated intraocular pressure (IOP) is the principal and sole treatable risk factor in POAG (Weinreb et al., 2016), which is regulated by aqueous humor (AH) production and drainage.

The conventional aqueous humor (AH) outflow pathway comprises trabecular meshwork (TM), juxtacanalicular connective tissue (JCT), and Schlemm’s canal (SC), which ends in the collector channels and aqueous veins (Johnstone et al., 2021). This drainage structure aids in the removal of 60%–80% of AH in humans (Hann and Fautsch, 2015). The TM tissue comprises beams of endothelial-type cells fenestrated by extracellular matrix (ECM) and is highly contractile, exhibiting a smooth muscle-like property (Pattabiraman and Toris, 2016). The TM is constantly stretched due to the ocular pulse created by the cardiac cycle, blinking, and eye movement inducing physiological changes in IOP (Johnstone, 2004a; Xin et al., 2017). The IOP is regulated due to the passage of AH across the TM-SC *via* the transcellular and/or paracellular flow (Raviola and Raviola, 1981; Johnson and Erickson, 2000). Though ECM imparts the resistance to fluid flow *via* the TM outflow pathway, it is not completely believed to be the only mechanistic impedance to AH drainage (Acott and Kelley, 2008; Pattabiraman and Toris, 2016; Acott et al., 2021; Johnstone et al., 2021). The distention and contraction phenomenon of TM-SC tissues is regulated by the elastic properties of the tissue (Stumpff and Wiederholt, 2000; Wiederholt et al., 2000). For the efficient function of TM, the elastic nature of the TM must be maintained. Additionally, in metabolically active tissues such as TM and SC (Reina-Torres et al., 2020), acute stress should induce protective measures to maintain tissue homeostasis. Failure to achieve homeostasis can lead to decreased AH flow and increased AH retention leading to elevated IOP. In fact, mechanistic evidence points out that experimental elevation of IOP can drive the cells toward an increased contractile phenotype by activating Rho GTPases, which is known to increase resistance to AH drainage (Pattabiraman et al., 2015a; Pattabiraman et al., 2015b). In glaucoma, the TM has a greater stiffness, and the elastic nature of the TM-SC is compromised (Last et al., 2011; Xin et al., 2016; Wang et al., 2017; Vahabikashi et al., 2019). The cardiac cycle sets up the pulsatile motion of the TM (Johnstone, 2004b). A loss of mechanosensation or transduction of such pulsatile mechanical stress can aid in TM stiffness, which in turn affects the pulsatile motion of the TM. A recent study using phase-sensitive optical coherence tomography (PhS-OCT) showed that the eyes with POAG had a significantly lower maximum velocity of the pulsatile motion and the motion amplitude of the TM compared to the healthy eye (Gao et al., 2020). This can be attributed to the increased stiffness and less elasticity of TM tissue. Thus, indicating a direct relationship between the mechanical stress on TM, TM elasticity, TM stiffness, and regulation of AH drainage.

A well-accepted model to study the mechanical stress on TM is the usage of cyclic mechanical stretch (CMS) (Matsuo et al., 1996; Liton et al., 2005; Vittal et al., 2005; Luna et al., 2009; Ramos et al., 2009; Youngblood et al., 2020). Studies have tested the effects of CMS on the changes in specific genes, proteins, and metabolites, including prostaglandins (Matsuo et al., 1996), matrix metalloproteases (Okada et al., 1998; Bradley et al., 2001; Keller et al., 2007), oculomedin (Sato et al., 1999), transforming growth factor beta-1 (TGF-β1) (Liton et al., 2005), and lysophosphatidic acid (Ho et al., 2018), as well as unbiased gene expression (Luna et al., 2009) and RNA sequencing studies (Youngblood et al., 2020). These studies show effective changes of TM under stretch in the processes, including calcium influx (Lakk et al., 2021), induction of autophagy (Shim et al., 2020; Shim et al., 2021), changes in actin networks (Duffy and O'Reilly, 2018), and upregulation of tyrosine phosphorylation of ECM protein in the TM (Maddala et al., 2017). Besides these studies toward understanding the response by TM subjected to CMS, studies involving global analysis of protein and lipid changes have not been performed, and an integrated analysis to investigate the underlying response mechanisms in TM subjected to mechanical stress is unavailable. Toward addressing the above lacunae in the field, we utilized a multiomics approach to better understand the physiological and pathological relevance of changes in the levels of RNA, proteins, and lipids exerted by CMS by combining mRNA sequencing, proteomics, and lipidomic analysis.

## Material and methods

### Materials

#### Reagents, antibodies, and materials

The reagents, antibodies, and materials used in this study were purchased as follows: Life Technologies (Austin, TX, United States)—TRIzol^TM^ LS Reagent (10296028); Zymo Research (Irvine, CA, United States)—Direct-zolTM RNA MiniPrep (R2050S); primary antibodies: Novus Biologicals LLC (Centennial, CO, United States)—anti-MKL2 (NBP1-46209) and anti-SREBP2 (NBP1-54446); Santa Cruz Biotechnology, (Dallas, TX, USA)—anti-HMGCS1 (sc-166763), anti-PAI-1 (sc-5297), anti-COL1A2 (sc-393573), and anti-GAPDH (sc-25778); rabbit anti-laminin HB-4 (anti-laminin-1 sera recognizing the total laminin including α1, β1, and γ1) (LeMosy et al., 1996) (gift from Dr. Harold Erickson, Duke University). Secondary antibodies conjugated with horseradish peroxidase (Jackson Immuno Research, West Grove, USA)—Donkey anti-mouse IgG (715-035-150) and anti-rabbit IgG (715-035-144). Cell Signaling Technology (Danvers, MA, United States) RIPA Buffer (9806S). GE Healthcare (Chicago, IL, United States)—nitrocellulose membrane (10600003).

### Primary trabecular meshwork cell culture

Primary human TM (HTM) cells were cultured from TM tissue isolated from the leftover donor corneal rings after they had been used for corneal transplantation at the Indiana University Clinical Service, Indianapolis, as described previously (Keller et al., 2018). HIPPA compliance guidelines have been adhered to for the use of human tissues. The usage of donor tissues was exempt from the DHHS regulation and the IRB protocol (1911117637), approved by the Indiana University School of Medicine IRB review board. The age, race, and sex of the donors were obtained from eye banks, which provided the corneas (Supplementary Figure S1). Briefly, TM tissue extracted from the corneal ring was chopped into fine pieces and placed in a 2% gelatin-coated 6-well tissue culture plate sandwiched by a coverslip. The tissues were grown in OptiMEM (Gibco, #31985-070), containing 20% FBS and penicillin-streptomycin-glutamine solution (Gibco, #10378-016). The expanded population of HTM cells was sub-cultured after 1–2 weeks in DMEM, containing 10% FBS and characterized by the detection of dexamethasone-induced myocilin (Supplementary Figure S1) using rabbit anti-myocilin antibody (generously provided by Dr. Daniel Stamer, Duke University).

### Cyclic mechanical stretch

Up to four biological replicates of HTM cells were seeded (∼40,000 cells/well) in collagen-coated plates (BioFlex plates, BF-3001C Flexcell International, Burlington, NC, United States) after counting using a hemocytometer. Once cells attain 90% confluency, cells were serum-starved for 3 h. Plates were placed on a Flexcell FX-6000 Tension System (Flexcell International, NC, USA). The cells were subjected to CMS at 0.69 Hz frequency with 15% stretching as published earlier (Luna et al., 2009; Shim et al., 2020; Youngblood et al., 2020) for 8 h for mRNA sequencing (HTM lines 1–4) and 24 h for proteomics and lipidomic analyses (HTM lines 1, 2, and 4). Control cells were cultured in the same conditions but were not subjected to CMS.

### mRNA sequencing

#### Sample preparation for mRNA sequencing

Post CMS, the cells were washed with 1XPBS and collected and homogenized in TRI reagent. The total RNA was extracted and purified using Direct-zol^TM^ RNA MiniPrep kit following the manufacturer’s protocol.

#### KAPA mRNA HyperPrep method for mRNA sequencing

Purified total RNA was first evaluated for its quantity and quality using Agilent Bioanalyzer 2100. For RNA quality, a RIN number of 7 or higher was utilized. One hundred nanograms of total RNA were used for preparing a cDNA library that includes mRNA purification/enrichment, RNA fragmentation, cDNA synthesis, ligation of index adaptors, and RNA amplification by following the KAPA mRNA Hyper Prep Kit Technical Data Sheet, KR1352—v4.17 (Roche Diagnostics, Basel, Switzerland). Each resulting indexed library was quantified, its quality was assessed by Qubit and Agilent Bioanalyzer, and multiple libraries were pooled in equal molarity. The pooled libraries were then denatured and neutralized before loading to NovaSeq 6000 sequencer at 300 pM final concentration for 100 b paired-end sequencing (Illumina, Inc.). Approximately 30–40 M reads per library were generated. A Phred quality score (Q score) was used to measure the sequencing quality. More than 90% of the sequencing reads reached Q30 (99.9% base call accuracy).

#### Mapping QC and data analysis

The sequencing data were first assessed using FastQC (Babraham Bioinformatics, Cambridge, United Kingdom) for quality control. Then, all sequenced libraries were mapped to the human genome (hg38) using STAR RNA-seq aligner (v.2.5) (Dobin et al., 2013) with the following parameter: “--outSAMmapqUnique 60.” The reads distribution across the genome was assessed using bamutils (from NGSUtils v.0.5.9) (Breese and Liu, 2013). Uniquely mapped sequencing reads were assigned to hg38 refGene genes using feature Counts (from subread v.1.5.1) (Liao et al., 2014) with the following parameters: “-s 2 –p–Q 10.” Differential expression analysis was performed using edgeR (Robinson et al., 2010), (McCarthy et al., 2012). Counts were normalized to counts per million reads (CPM) for each sample. Data were examined by Multidimensional Scaling in the edgeR package (Robinson et al., 2010) to detect outliers. The data were normalized using the TMM (trimmed mean of M values) method. False discovery rates (FDR) were calculated using the Benjamini & Hochberg method (Benjamini and Hochberg, 1995) within edgeR. All raw and processed data are available *via* Gene expression omnibus (GEO).

The GEO accession: GSE195756

### Proteomics

#### Sample preparation for proteomics

Cell pellets were lysed in 8 M urea (Bio-Rad Laboratories Inc., CA, United States, 161-0731), 100 mM Tris-HCl, pH 8.5 (Sigma-Aldrich, St. Louis, MO, United States, 10812846001) by sonication in 1.5 ml Micro Tubes (TPX Plastic for Sonication from Diagende Inc.) using a Bioruptor® sonication system (Diagenode Inc., New Jersey, United States, B01020001) with 30 s/30 s on/off cycles for 15 min in a water bath at 4°C. After subsequent centrifugation at 14,000 rcf for 20 min, protein concentrations were determined by Bradford protein assay (BioRad, Hercules, 5000006). A 20 µg equivalent of protein from each sample was reduced with 5 mM tris(2-carboxyethyl) phosphine hydrochloride (TCEP, Sigma-Aldrich, C4706) for 30 min at room temperature, and the resulting free cysteine thiols were alkylated with 10 mM chloroacetamide (CAA, Sigma-Aldrich, C0267) for 30 min at room temperature in the dark. Samples were diluted with 50 mM Tris HCl, pH 8.5, to a final urea concentration of 2 M for Trypsin/Lys-C based overnight protein digestion at 37°C (1:100 protease: substrate ratio, mass spectrometry grade, Promega Corporation, Madison, WI, V5072).

#### Peptide purification and labeling

Digestions were acidified with trifluoracetic acid (TFA, 0.5% v/v) and desalted on Sep-Pak® Vac cartridges (Waters^TM^, Milford, Massachusetts, United States, WAT054955) with a wash of 1 ml 0.1% TFA followed by elution in 70% acetonitrile 0.1% formic acid (FA). Peptides were dried by speed vacuum and resuspended in 29 µL of 50 mM triethylammonium bicarbonate. Peptide concentrations were checked by Pierce Quantitative colorimetric assay (Thermo Fisher Scientific, Waltham, Massachusetts, United States, 23275). The same amount of peptide from each sample was then labeled for 2 hours at room temperature with 0.2 mg of Tandem Mass Tag reagent (Thermo Fisher Scientific, TMT™ Isobaric Label Reagent Set, 90309, lot no. VI307195B). Labeling reactions were quenched by adding 0.3% hydroxylamine (v/v) to the reaction mixtures at room temperature for 15 min. Labeled peptides were then mixed and dried by speed vacuum.

#### High pH basic fractionation

For high pH basic fractionation, peptides were reconstituted in 0.1% TFA and fractionated using methodology and reagents from Pierce™ High pH reversed-phase peptide fractionation kit (8 fractions, ThermoFisher, A32993).

#### Nano-LC-MS/MS analysis

Nano-LC-MS/MS analyses were performed on an EASY-nLC HPLC system (SCR:014993, Thermo Fisher Scientific) coupled to Orbitrap Exploris 480™ mass spectrometer (Thermo Fisher Scientific) with a FAIMS pro interface. About 1/8 of each global peptide fraction and 1/4 of each phosphopeptide fraction were loaded onto a reversed-phase EasySpray™ C18 column (2 μm, 100 Å, 75 μm × 25 cm, Thermo Scientific, ES902A) at 400 nl/min. Peptides were eluted from 4%–30% with mobile phase B (mobile phases A: 0.1% FA, water; B: 0.1% FA, 80% acetonitrile (Thermo Fisher Scientific, LS122500) over 160 min, 30%–80% B over 10 min, and dropping from 80%–10% B over the final 10 min. The mass spectrometer was operated in positive ion mode with a 4 s cycle time data-dependent acquisition method with advanced peak determination. The FAIMS CV was maintained at −50 V. Precursor scans (m/z 375-1600) were done with an orbitrap resolution of 60,000, RF lens% 40, maximum inject time 50 ms, normalized AGC target 300%, and MS2 intensity threshold of 5e4, including charges of 2–6 for fragmentation with 60 s dynamic exclusion. MS2 scans were performed with a quadrupole isolation window of 0.7 m/z, 35% HCD CE, 45,000 resolution, 200% normalized AGC target, auto maximum IT, and fixed first mass of 110 m/z.

#### Data analysis

The resulting RAW files were analyzed in Proteome Discover™ 2.4 (Thermo Fisher Scientific) with a Homo sapiens UniProt FASTA (last modified 021517) plus common contaminants. SEQUEST HT searches were conducted with a maximum number of two missed cleavages, precursor mass tolerance of 10 ppm, and a fragment mass tolerance of 0.02 Da. Static modifications used for the search were as follows: 1) carbamidomethylation on cysteine (C) residues, 2) TMT6plex label on N-peptide N-termini, and 3) TMT6plex label on lysine (K) residues. Dynamic modifications used for the search were oxidation of methionines and acetylation, Met-loss, or Met-loss plus acetylation of protein N-termini. The percolator false discovery rate was set to a strict setting of 0.01 and a relaxed setting of 0.050. In the consensus workflow, peptides were normalized by total peptide amount with no scaling. Co-isolation of 50% and average reporter ion S/N 10 were used as thresholds for quantification. Resulting normalized abundance values for each sample type, abundance ratio and log_2_(abundance ratio) values (Supplementary Figure S2), and respective *p*-values (ANOVA) from Proteome Discover™ were exported to Microsoft Excel. All raw and processed data are available *via* ProteomeXchange.

Project accession: PXD031347

Project DOI: 10.6019/PXD031347

### Lipidomics

#### Cellular lipid extraction and multiple reaction monitoring profiling-based MS for lipidomics

Samples represented by pellets containing around 50,000 cells were processed using the Bligh and Dyer protocol (Bligh and Dyer, 1959). Immediately after stretch, the cell lysis was achieved by adding 200 μl of ultrapure water and repeated pipetting for 1 minute. After that, 550 μl of methanol and 250 μl of chloroform were added, and the samples were incubated for 15 min at 4°C. Then, 250 μl of ultrapure water and 250 μl of chloroform were added, causing the solution to become biphasic. Phase separation was improved by centrifugation at 10,000 rpm for 5 min. The bottom phase (lipid extract) was then transferred to a clean microtube, and the extracts were dried using a SpeedVac centrifuge. Dried lipid extracts were diluted in 5 μl of chloroform and 45 μl of injection solvent (acetonitrile/methanol/ammonium acetate 300 mM 3:6.65:0.35 [v/v]) to obtain a stock solution. The stock solution was diluted ten times into injection solvent spiked with 0.1 ng/μl of EquiSPLASH Lipidomics (Avanti Polar Lipids, AL, United States #330731) for sample injection. The MRM-profiling methods and instrumentation used have been recently described in previous reports (de Lima et al., 2018) (Dipali et al., 2019) (Suárez-Trujillo et al., 2020) (Clyde-Brockway et al., 2021). In summary, data acquisition was performed using flow injection (no chromatographic separation) from 8 μl of the diluted lipid extract stock solution delivered using a micro-autosampler (G1377A) to the ESI source of an Agilent 6410 triple quadrupole mass spectrometer (Agilent Technologies, Santa Clara, CA, United States). A capillary pump was connected to the autosampler and operated at a flow rate of 7 μl/min and a pressure of 100 bar. The capillary voltage on the instrument was 5 kV, and the gas flow was 5.1 L/min at 300°C. The MS data obtained from these methods were processed using an in-house script to obtain a list of MRM transitions with their respective sum of absolute ion intensities over the acquisition time.

#### Cell culture media lipid extraction and high-performance liquid chromatography-mass spectrometry

For extracellular lipid analysis, control and stretched HTM cell culture media were collected (around 6 ml), and the speed vacuum was concentrated (SpeedVac DNA 130, Thermo Fisher Scientific) to generate the pellets. The lipids were extracted using the Bligh& Dyer protocol as described above (Bligh and Dyer, 1959). Lipid samples were eluted using reverse phase liquid chromatography with a Hypersil Gold C18 column (particle size 1.9 uM, 150 × 2.1 mm ID, Thermo Fisher Scientific). The column was used with the Vanquish Horizon UHPLC System (Thermo Fisher Scientific) equipped with an autosampler and binary pump combination. The HPLC system was coupled to a Q-Exactive mass spectrometer (Thermo Fisher Scientific) for high-resolution mass spectrometry analysis. The column temperature was 55°C, and the injection volume was 8 μl. A gradient of 30 min with a flow rate of 0.260 ml/min was run from 10% to 100% solvent B. Solvent A was a 50:50 ratio of acetonitrile:water +0.1% FA + 5 mM ammonium formate, and Solvent B was 88:10:2 ratio of isopropanol:acetonitrile:water +0.1% formic acid + 5 mM ammonium formate. A heated electrospray ionization (HESI) source was used as an ionization method and coupled to the Q-Exactive instrument. The conditions for the HESI were as follows: spray voltage was 4.00 kV for positive mode and 2.50 kV for negative mode. The heated capillary temperature was set to 325°C and the auxiliary heater to 275°C. The sheath gas flow rate was set to 35 units and the aux gas flow rate to 15 units. The S-lens RF level was 70. The resolution for the full scan was set to 70,000 at a mass range of 250–1,200 m/z. The automatic gain control target was 1 × 10^6, and the maximum injection time (IT) was 240 ms. The data-dependent acquisition had a resolution of 17,500 and an AGC target of 1 × 10^5 with a maximum IT of 50 ms. The isolation window was 1.0 m/z, and the collision energies were set to 20, 30, and 40 eV.

Raw files from the mass spectrometer were analyzed for lipid identification using LipidSearch software version 4.2. The following parameters were used for identification: parent and product search tolerance of 5 ppm. Filters were set at the top rank, main isomer peak, and FA priority, with an m-score threshold of 5.0 and a c-score threshold of 2.0. Quantification was set with an m/z tolerance of 5 ppm and retention time tolerance of 0.5 min. The following adducts were allowed in positive mode: [M + H]+, [M + NH4]+, [M + 2H]2+, and [M − H]−, [M − 2H]2−, [M − CH3] − in negative mode. All lipid classes were selected for search except for glycoglycerolipids and derivatized lipids.

All potential lipid matches for each sample were aligned together for the positive and negative modes. All technical replicates were aligned together. The retention time set for the alignment was 0.1 min, with top-ranked filtered and main isomer peak selected. All peaks with the same annotated lipid species were merged in the result.

#### Lipidomics data analysis

Statistical analysis was performed utilizing MetaboAnalyst 5.0 (http://www.metaboanalyst.ca/). Data on the relative amounts were auto-scaled to obtain a normal distribution and evaluated by principal component analysis (PCA) to find the directions that best explain the variance in the dataset, cluster analysis/heatmap to visualize the concentration values in the data table, and paired *t*-test to compare different lipid classes content in control and stretched TM cells/media. Informative lipids were analyzed according to class, fatty acyl residue chain length, and unsaturation level.

### Data integration analysis

The RNA-seq, proteomics, and lipidomics data were integrated based on the differentially expressed genes and proteins and regulated lipid classes. The common up- and downregulated genes were matched with the proteins using gene names. For pathway analysis, comparison between differentially expressed genes and proteins based on pathways involving cytoskeleton organization (actin- and tubulin-related), ECM organization, apoptosis, oxidative stress (redox), lipid metabolism, autophagy, and proteostasis (chaperones and protein quality control) were filtered. These were fed on STRING search (https://string-db.org/) to obtain predicted interaction maps for each pathway. Lipid classes known to be involved in the regulation of the pathway mentioned above were compared with differentially expressed genes and proteins.

### Western blotting

The cell lysates containing total protein were prepared using 1X RIPA buffer composed of 50 mM Tris-HCl (pH 7.2), 150 mM NaCl, 1% NP-40, 0.1% SDS, 1 mM EDTA, and 1 mM PMSF with protease and phosphatase inhibitors and then sonicated. The protein concentration was determined using Bradford Assay Reagent. A total of 25–40 μg of the protein sample was mixed with 4X Laemmli buffer and separated on 8%–10% SDS polyacrylamide gel. Following the gel run, the proteins were transferred to a 0.45 µM pore size nitrocellulose membrane. Ponceau S staining of the membrane was performed to document protein loading after transfer. Membranes were blocked in either 5% non-fat dry milk or 5% BSA in Tris-buffered saline with 0.1% Tween for 2 h, followed by respective primary antibodies overnight at 4°C (∼16 h) and then horseradish peroxidase-conjugated secondary antibodies (Jackson Immuno Research). The blots were washed with 1X TBST, and the immunoreactivity was detected using Western Lightning Plus Enhanced Chemiluminescence (ECL) Substrate (Perkin Elmer, Shelton, United States) and imaged in a ChemiDoc MP imaging system (Bio-Rad). Blots were stripped using mild stripping buffer if required to reprobe for the loading control and multiple proteins within the same molecular weight range. Data were normalized to GAPDH. Semi-quantitative analyses and fold changes were calculated from the band intensities measured using ImageJ software.

### Statistical analysis

All data are presented as the mean ± standard error of the mean (SEM) of biological replicates of four independent observations for RNA-seq and three independent observations for proteomics and lipidomics mass spectrometry analysis. For immunoblotting, four independent biological replicates were used. GraphPad Prism 8 was used to generate graphs. Quantitative data were analyzed by the student’s paired *t*-test. *p*-value ≤ 0.050 was considered statistically significant.

## Results

### Differential expression of genes in TM subjected to CMS

**FIGURE 1:**
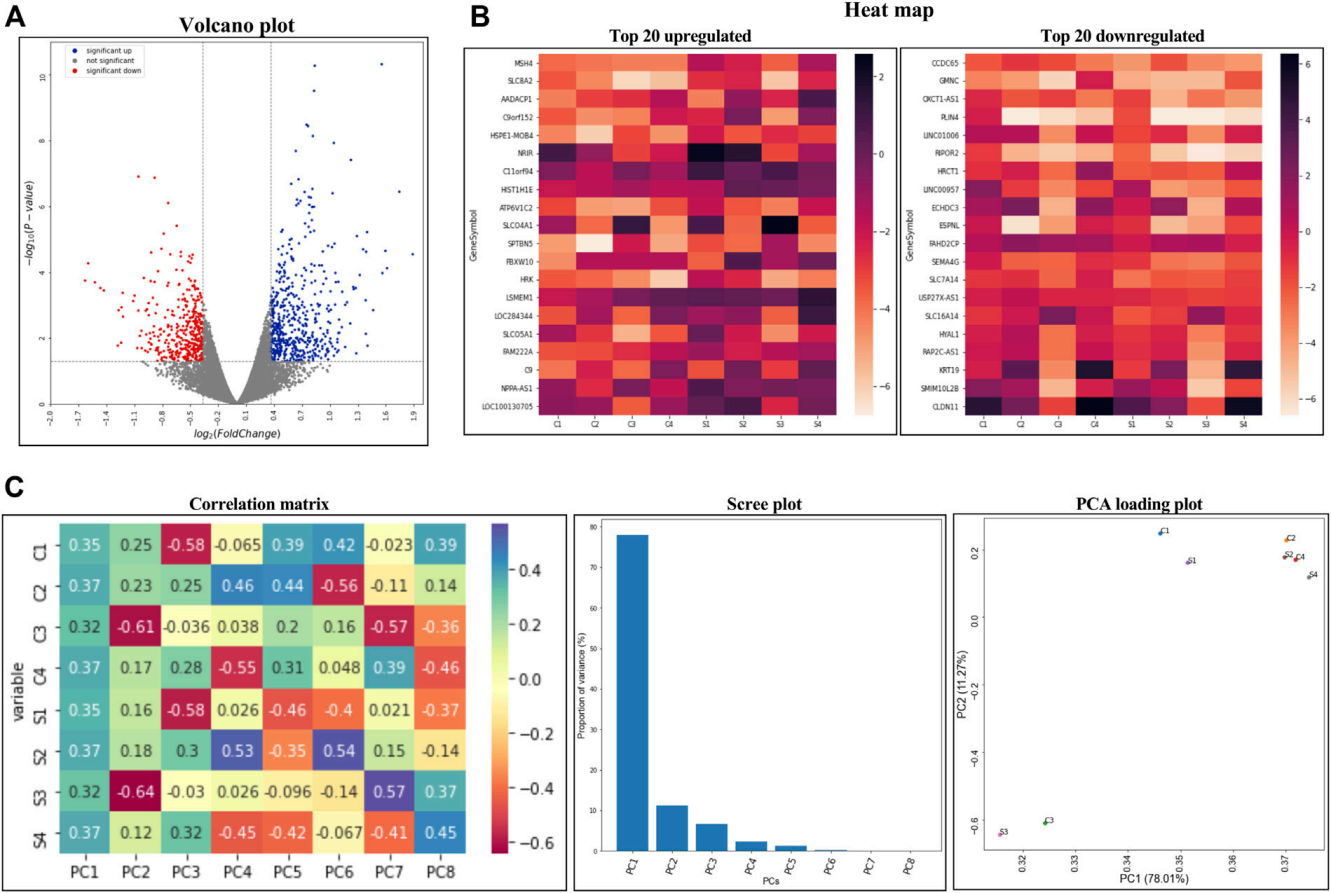
Transcriptomics analysis of RNA-seq data. **(A)** Volcano plot of all genes significantly regulated by cyclic mechanical stress. **(B)** Heatmaps of the top 20 upregulated (left) and top 20 downregulated (right) transcripts abundances based on Log_2_ FPKM (fragments per kilobase of transcript per million mapped reads). **(C)** PCA on the correlation matrix (left), scree plot representing the eigenvalues and the proportion of variance accounted for by the principal components (middle), and 2D PCA loading plots for the top 2 (PC1 and PC2) components (right).

Primary HTM cells from four biological replicates were subjected to CMS for 8 h to evaluate the effects of mechanical stress on the gene expression profile in TM. We focused on the 8 h window because the estimated median mRNA half-life in human cells is approximately 10 h (Yang et al., 2003). The mRNA sequencing analysis identified 14,942 transcripts. Based on the statistical significance (*p* ≤ 0.050) and false discovery rate (FDR) < 6%, 211 genes were identified to be differentially expressed. A systematic evaluation of the RNA-seq data is shown in Figure 1. The volcano plot (Figure 1A) with all genes significantly modulated due to CMS—red dots represent downregulated genes, blue dots represent upregulated genes, and grey dots represent the unchanged genes—compared to the controls. Among them, based on absolute log_2_(fold change), 148 genes were upregulated (≥0.3 log_2_FC), and 63 genes were downregulated (≤0.3 log_2_FC) (Supplementary Table S1) in HTM cells subjected to CMS. Figure 1B represents the heatmap of the top 20 upregulated and downregulated genes. The PCA correlation matrix (Figure 1C, left), scree plot representing eigenvalues (proportion of variance) (Figure 1C middle), and the elbow nature of the graph indicating PCA worked well on the data and 2D PCA loading plots for the top two components are shown in Figure 1C (right). The top 20 upregulated genes are provided in Table 1.

**TABLE 1:** Top 20 upregulated genes.

Gene symbol	Description	Fold change	log_2_FC	*p*-value
MSH4	mutS homolog 4	5.827	2.543	4.8E-10
SLC8A2	Solute carrier family 8 member A2	4.425	2.146	4.5E-05
AADACP1	Arylacetamide deacetylase pseudogene 1	4.347	2.120	2.1E-06
C9orf152	Chromosome 9 open reading frame 152	4.081	2.029	2.0E-04
HSPE1-MOB4	HSPE1-MOB4 readthrough	3.702	1.888	2.8E-05
NRIR	Negative regulator of interferon response	3.351	1.745	3.6E-07
C11orf94	Chromosome 11 open reading frame 94	3.059	1.613	7.6E-05
HIST1H1E	H1.4 Linker Histone, cluster member	3.022	1.595	2.3E-05
ATP6V1C2	ATPase H+ transporting V1 subunit C2	2.955	1.563	1.1E-04
SLCO4A1	Solute carrier organic anion transporter family member 4A1	2.940	1.556	4.9E-11
SPTBN5	Spectrin beta, non-erythrocytic 5	2.637	1.399	6.1E-06
FBXW10	F-box and WD repeat domain containing 10	2.618	1.389	2.4E-05
HRK	Harakiri, BCL2 interacting protein	2.609	1.383	7.1E-04
LSMEM1	Leucine rich single-pass membrane protein 1	2.560	1.356	2.0E-05
LOC284344	Uncharacterized LOC284344	2.497	1.320	1.2E-04
SLCO5A1	Solute carrier organic anion transporter family member 5A1	2.444	1.289	8.0E-06
FAM222A	Family with sequence similarity 222 member A	2.424	1.277	3.0E-04
C9	Complement C9	2.387	1.255	3.3E-04
NPPA-AS1	NPPA antisense RNA 1	2.379	1.250	2.8E-04
LOC100130705	ATP6V1F neighbor	2.338	1.225	3.9E-08

Top 20 genes significantly upregulated in TM subjected to CMS with FDR < 6% and log_2_FC ≥ 0.3. Columns (left to right) show the gene symbol, gene description, fold change, log_2_FC determined by mRNA sequencing analysis, and calculated *p*-value, where *p* < 0.050 was statistically significant.

Interestingly, the most upregulated gene under CMS was mutS homolog 4 (MSH4) (2.54-log_2_FC, 5.83-FC), which is involved in meiotic recombination (Kneitz et al., 2000) and protein ubiquitination pathway (Xu and Her, 2013). Several stress-related genes were differentially expressed: a) heat shock protein 70 family (Radons, 2016), HSPA5 (0.60-log_2_FC; 1.51-FC), HSPA1B (0.92-log_2_FC; 1.90-FC); b) DnaJ/heat shock protein 40 family, DNAJA1 (0.46-log_2_FC; 1.37-FC), DNAJB1 (0.45-log_2_FC; 1.36-FC), DNAJB9 (0.76-log_2_FC; 1.70-FC); c) heat shock protein family H (Hsp110) member 1 (HSPH1) (0.73-log_2_FC; 1.66-FC); d) heat shock protein family B (small) member 8 (HSPB8) (0.43-log_2_FC; 1.35-FC); and e) co-chaperone for HSP90 cysteine and histidine-rich domain containing 1 (CHORDC1) (0.66-log_2_FC; 1.583-FC) were upregulated significantly. Under CMS, there was significant upregulation of actin-cytoskeleton associated genes including SLIT-ROBO rho GTPase-activating protein 1 (SRGAP1) (0.39-log_2_FC; 1.31-FC), spectrin-β chain, non-erythrocytic 5 (SPTBN5) (1.40-log_2_FC; 2.64-FC), formin-like 2 (FMNL2) (0.38-log_2_FC; 1.30-FC), and RAS like proto-oncogene A (RalA) (0.43-log_2_FC; 1.348-FC). Additionally, both frizzled receptor class, FZD4 (0.65-log_2_FC; 1.57-FC) and FZD8 (1.04-log_2_FC; 2.06-FC) genes, were upregulated, which are involved in Wnt/β-catenin canonical signaling pathway (Gurney et al., 2012). On the contrary, Dickkopf WNT signaling pathway inhibitor 1 (DKK1) gene, a Wnt signaling inhibitor (Semënov et al., 2008), was also increased (0.54-log_2_FC; 1.45-FC), indicating a homeostatic transcriptional control of Wnt signaling when TM is under mechanical stress. Further, looking at genes involved in ECM and fibrosis regulation, sphingosine kinase (SphK1) (0.83-log_2_FC; 1.78-FC), a rate-limiting enzyme in the synthesis of profibrotic sphingosine-1-phosphate (S1P) (Fyrst and Saba, 2010) was augmented, and countering fibrosis increased in membrane-type metalloprotease 16 (MMP16) (0.36-log_2_FC; 1.29-FC) involved in ECM degradation and Mothers against decapentaplegic homolog 7 (Smad7) (0.5-log_2_FC; 1.42-FC), a negative regulator of the transforming growth factor β and fibrosis (Zhu et al., 2011).

Intriguingly, we found that genes involved in transmembrane transport were upregulated: a) calcium ion transport (solute carrier family 8 member A2) (SLC8A2) (2.1-log_2_FC; 4.43-FC); b) myo-inositol transport (solute carrier family 5 member 3) (SLC5A3) (0.81-log_2_FC; 1.76-FC); c) amino-acid transport (solute carrier family 38 member 2) (SLC38A2) (0.59-log_2_FC; 1.50-FC); d) glycine transport (solute carrier family 6 member 9) (SLC6A9) (0.58-log_2_FC; 1.50-FC); and e) acetyl-coenzyme A transporter (solute carrier family 33 member 1) (SLC33A1) (0.39-log_2_FC; 1.32-FC). These proteins aid in ions, solutes, and metabolites across the biological membranes (Sahoo et al., 2014). Among the upregulated mRNA, three non-coding RNA—LOC284344 (1.32-log_2_FC; 2.50-FC), LOC100379224 (0.56-log_2_FC; 1.47-FC), and LOC646762 (0.47-log_2_FC; 1.39-FC) —and two uncharacterized protein-coding RNA—LOC100130705 (1.23-log_2_FC; 2.34-FC) (predicted protein, ATP6V1FNB, UniProtKB, A0A1B0GUX0) and C11orf94 (1.61-log_2_FC; 3.06-FC) (UniProtKB - C9JXX5)—were found. The network map of all the upregulated genes in TM subjected to CMS is provided inSupplementary Figure S3.

Gene ontology enrichment analysis for upregulated genes in TM under CMS based on its molecular function and the biological process performed using ShinyGO v0.75 (Ge et al., 2020) are given in Table 2 and Supplementary Table S2, respectively. Based on molecular functions, most of the upregulated genes were associated with membrane transporter (eight genes), protein ubiquitination (nine genes), transcription repression (eight genes), stress-response (six genes), chaperone activity (five genes), and SMAD binding (four genes) (Table 2). Pathway enrichment analysis of genes based on their biological process (Supplementary Table S2) demonstrated genes that were upregulated and involved in transmembrane signaling, signal transduction, responders to stimuli such as stress, and the SMAD signaling pathway.

**TABLE 2:** Pathway enrichment analysis for upregulated genes using ShinyGO based on molecular function.

Pathway	Genes
Active and secondary ion transmembrane transporter activity	SLCO4A1 SLC8A2 SLCO5A1 SLC33A1 SLC5A3 SLC38A2 SLC6A9
Organic anion transmembrane transporter activity	SLCO4A1 SLC38A2 SLCO5A1 ABCC5 SLC33A1 SLC6A9
Neutral amino acid:sodium symporter activity	SLC38A2 SLC6A9
Ubiquitin protein ligase binding	TRAF1 HSPA1B TRIB1 RALA HEL-S-89n DNAJA1 SMAD7 FZD4 FZD8
Heat shock protein binding	HSPA5 DNAJB1 HSPA1B DNAJA1 DNAJB9 CHORDC1
DNA-binding transcription repressor activity	ZBTB1 ZBTB2 BHLHB2 SKIL JDP2 HES1 E2F7 HEY1
Chaperone binding	DNAJB1 DNAJA1 HES1 DNAJB9 HSPA5
SMAD binding	SMAD7 PMEPA1 SKIL LDLRAD4

Columns (left to right) show the pathways and genes involved in the given pathway in TM subjected to CMS, based on molecular function using ShinyGO pathway enrichment analysis.

On examining the 63 downregulated genes, we found that the coiled-coil domain containing 65 (CCDC65) gene (−1.629-log_2_FC; −3.09-FC) was the most significantly downregulated. Table 3 shows the top 20 downregulated genes. We found that the only stress-response gene that was downregulated was DnaJ heat shock protein family (Hsp40) member B5 (DNAJB5) (−0.71-log_2_FC; −1.64-FC). Other notable downregulated genes included the pro-apoptotic gene thioredoxin interacting protein (TXNIP) (−0.57-log_2_FC; −1.49-FC), rho family interacting cell polarization regulator 2 (RIPOR2), and a negative regulator of small GTPase Rho (−1.23-log_2_FC; −2.34-FC). Neural crest cells are progenitors of TM (Kaiser-Kupfer, 1989), and the expression of keratin 19 (KRT19), a neural crest marker (Lignell et al., 2017), was downregulated (−0.79-log_2_FC; −1.72-FC) due to CMS. Hyaluronidase-1, an enzyme degrading hyaluronan in the ECM, was downregulated significantly (−0.85-log_2_FC; −1.80-FC). Tight junction adhesion molecule claudin 11 (CLDN11) (−0.74-log_2_FC; −1.67-FC) and cell adhesion molecule vascular cell adhesion molecule 1 (VCAM1) (−0.52-log_2_FC; −1.44-FC) were decreased significantly indicating a potential loss of the cell–cell contact and communication in TM endothelial cells (Cerutti and Ridley, 2017).

**TABLE 3:** Top 20 downregulated genes.

Gene symbol	Description	Fold change	log_2_FC	*p*-value
CCDC65	Coiled-coil domain containing 65	−3.093	−1.629	1.80E-04
GMNC	Geminin coiled-coil domain containing	−3.023	−1.596	5.371E-05
OXCT1-AS1	OXCT1 antisense RNA 1	−2.876	−1.524	2.02E-04
PLIN4	Perilipin 4	−2.761	−1.465	0.0003009
LINC01006	Long intergenic non-protein coding RNA 1006	−2.691	−1.428	0.0003568
RIPOR2	RHO family interacting cell polarization regulator 2	−2.339	−1.226	4.23E-04
HRCT1	Histidine-rich carboxyl terminus 1	−2.163	−1.113	0.0007467
LINC00957	Long intergenic non-protein coding RNA 957	−2.142	−1.099	5.14E-04
ECHDC3	Enoyl-CoA hydratase domain containing 3	−2.079	−1.056	1.24E-07
ESPNL	Espin-like	−1.998	−0.998	0.0001494
FAHD2CP	Fumarylacetoacetate hydrolase domain containing 2C, pseudogene	−1.899	−0.925	1.86E-04
SEMA4G	Semaphorin 4G	−1.892	−0.920	2.50E-05
SLC7A14	Solute carrier family 7 member 14	−1.868	−0.902	5.99E-04
USP27X-AS1	USP27X antisense RNA 1 (head to head)	−1.845	−0.883	0.0001952
SLC16A14	Solute carrier family 16 member 14	−1.844	−0.883	1.36E-07
HYAL1	Hyaluronidase 1	−1.798	−0.847	9.25E-05
RAP2C-AS1	RAP2C antisense RNA 1	−1.752	−0.809	1.94E-05
KRT19	Keratin 19	−1.723	−0.785	6.72E-06
SMIM10L2B	Small integral membrane protein 10 like 2B	−1.676	−0.745	0.0002565
CLDN11	Claudin 11	−1.667	−0.737	7.94E-07

Top 20 genes significantly downregulated in TM subjected to CMS with FDR<6% and log_2_FC ≤ −0.3. Columns (left to right) show the gene symbol, gene description, fold change, log_2_FC determined by mRNA sequencing analysis, and calculated *p*-value, where *p* < 0.050 was statistically significant.

Interestingly, two lipid metabolism-related genes, including carnitine palmitoyltransferase 2 (CPT2) (−0.51-log_2_FC; −1.42-FC) and short-chain specific acyl-CoA dehydrogenase (ACADS) (−0.62-log_2_FC; −1.53-FC), which are involved in fatty acid beta-oxidation (Houten and Wanders, 2010), went down significantly. Pathway enrichment analysis for downregulated genes was performed using the ShinyGO v0.75 (Ge et al., 2020) tool based on their molecular function (Table 4). Based on the molecular functions, our results indicate that CMS on TM negatively regulated the cellular metabolic process at the transcription levels. A network map of downregulated genes in TM subjected to CMS is provided in Supplementary Figure S4. The original mRNA sequencing data files are available at GEO under the GEO accession: GSE195756.

**TABLE 4:** Pathway enrichment analysis for downregulated genes using ShinyGO based on molecular function.

Pathway	Genes
Beta-Alanine metabolism	ALDH3A1 ALDH3B1 ACADS
Histidine metabolism	ALDH3A1 ALDH3B1
Phenylalanine metabolism	ALDH3A1 ALDH3B1
Peroxisome	CRAT IDH2 PEX11B
Metabolic pathways	ALDH3A1 ALDH3B1 FECH GCNT1 HYAL1 IDH2 ACADS MPI PLCD1 ETNK2
AGE-RAGE signaling pathway in diabetic complications	PLCD1 CCL2 VCAM1
Tyrosine metabolism	ALDH3A1 ALDH3B1
Fatty acid degradation	CPT2 ACADS

Columns (left to right) show the pathways and genes involved in the given pathway in TM subjected to CMS, based on molecular function using ShinyGO pathway enrichment analysis.

### Differential expression of proteins in TM subjected to CMS

For proteomics analysis, TM cells were subjected to CMS for 24 h. The time provided for stretch and examination of protein changes was based on published data (Chen et al., 2016). The published study suggested a median half-life of a protein of around 9 h out of the 804 proteins traced for half-life. Nevertheless, roughly 272 proteins had a half-life ranging between 10 and 36 h (Chen et al., 2016). Therefore, we decided to examine protein changes after 24 h of CM using a TMT-based LC/MS-MS proteomics approach. In total, 4,758 proteins were detected. Further, based on FDR ≤5% and statistical significance (*p* ≤ 0.050), 147 proteins were significantly upregulated and 66 were significantly downregulated. Based on mean ± 2σ of log_2_ ≥ 0.3 of confidence fold change limits (Supplementary Figure S2), 10 proteins were upregulated and 29 proteins were downregulated. Further, using the criteria mean ± 2σ of log_2_ < 0.3–0.1, 82 proteins were upregulated and 17 proteins were downregulated in TM under CMS (Supplementary Table S3). The list of top 10 upregulated proteins is provided in Table 5.

**TABLE 5:** Top 10 upregulated cellular proteins.

Gene symbol	Accession	Description	Fold change	log_2_FC	*p*-value
METTL13	Q8N6R0	Methyltransferase-like protein 13	1.879	0.910	0.035
SFSWAP	Q12872	Splicing factor, suppressor of white-apricot homolog	1.399	0.480	0.031
FAM8A1	Q9UBU6	Protein FAM8A1	1.324	0.410	0.050
SLC38A2	Q96QD8	Sodium-coupled neutral amino acid transporter 2	1.308	0.390	0.038
GATAD2B	Q8WXI9	Transcriptional repressor p66-beta	1.312	0.390	0.018
SNRNP27	Q8WVK2	U4/U6.U5 small nuclear ribonucleoprotein 27 kDa protein	1.3	0.380	0.012
NRBF2	Q96F24	Nuclear receptor-binding factor 2	1.272	0.350	0.007
ATXN3	P54252	Ataxin-3	1.263	0.340	0.014
FDFT1	P37268	Squalene synthase	1.256	0.330	0.044
MKL2	Q9ULH7	MKL/myocardin-like protein 2	1.249	0.320	0.020

Top 10 proteins upregulated in TM subjected to CMS with FDR ≤ 5% and log_2_FC ≥ 0.3. Columns (left to right) show the gene symbol, UniProt accession number, protein description, fold change, log_2_FC determined by global proteomic analysis, and calculated *p*-value, where *p* < 0.050 was statistically significant.

The most significantly upregulated protein was methyltransferase-like protein 13 (METTL13) (0.91-log_2_FC; 1.879-FC), which catalyzes protein methylation of elongation factor 1-alpha (Jakobsson et al., 2018), indicating a potential increase in protein synthesis (Jakobsson et al., 2017; Jakobsson et al., 2018). Many proteins that bind to and/or are associated with actin cytoskeleton organization were upregulated. Among them, the significant ones were a) ataxin-3 (0.34-log_2_FC; 1.26-FC), which is involved in cytoskeletal organization and cell survival (Rodrigues et al., 2010), and b) MKL/myocardin-like protein 2 (MKL2) which was upregulated (0.32-log_2_FC; 1.25-FC), a coactivator that binds to transcriptional factor serum response factor (SRF), is regulated by activation of Rho GTPase, and induces genes involved in myogenic differentiation, actin cytoskeletal organization, focal adhesion assembly, and tissue fibrosis (Pipes et al., 2006; Pattabiraman and Rao, 2010; Pattabiraman et al., 2014; Xu et al., 2015). Other upregulated cytoskeleton-related and ECM proteins were cytoskeleton binding protein thymosin beta 4 (TMSB4X) (0.13-log_2_FC; 1.10-FC) (Xue et al., 2014) and tropomodulin-3 (TMOD3) (0.14-log2FC; 1.11-FC) involved in F-actin stabilization (Parreno and Fowler, 2018), collagen 1A2 (COL1A2), which is a part of a large molecule type I collagen (0.14-log_2_FC; 1.10-FC), pro-fibrotic mothers against decapentaplegic homolog 3 (SMAD3) (0.11-log_2_FC; 1.08-FC) (Flanders, 2004), and laminin subunit gamma-1 (0.09-log_2_FC; 0.09-FC), though it did not fall under our stringency criteria for consideration (log_2_FC > 0.1). Mechanical stress on TM triggered upregulation of anti-apoptotic proteins such as DnaJ homolog subfamily A member 1 (DNAJA1) (0.46-log_2_FC; 1.12-FC) significantly and the death domain-associated protein 6 (DAXX) (0.14-log_2_FC; 1.10-FC) and tax1-binding protein 1 (TAX1BP1) (0.12-log_2_FC; 1.08-FC). Additionally, nuclear receptor-binding factor 2 (NRBF2) (0.35-log_2_FC; 1.27-FC) and maturation- gamma-aminobutyric acid receptor-associated protein-like 1 (GABARAPL2) (0.21-log_2_FC; 1.16-FC)—proteins involved in autophagosome formation (Behrends et al., 2010; Birgisdottir et al., 2019)—were upregulated.

Interestingly, mechanical stretching of TM upregulated proteins was associated with cholesterol biosynthesis. Hydroxymethylglutaryl-CoA synthase (HMGCS1) (0.21-log_2_FC; 1.16-FC), isopentenyl-diphosphate delta isomerase 1 (IDI1) (0.12-log_2_FC; 1.08-FC), and squalene synthase (FDFT1) (0.33-log_2_FC; 1.26-FC) were all upregulated in TM subjected to CMS, thus indicating the modulation of membrane rigidity, exocytosis, and mechanotransduction (Najafinobar et al., 2016; Lakk et al., 2021) under mechanical stress. Pathway enrichment of upregulated proteins by ShinyGO analysis (Ge et al., 2020) based on molecular function and biological process are given in Table 6 and Supplementary Table S4, respectively. The molecular functions of most of the upregulated proteins in TM subjected to CMS were strongly associated with RNA binding and/or involved in post-translational modifications. Network analysis by STRING shows a map of upregulated proteins whose biological functions are interlinked (Supplementary Figure S5).

**TABLE 6:** Pathway enrichment analysis for upregulated cellular proteins using ShinyGO based on molecular function.

Pathway	Proteins
RNA binding	BCLAF1 STAU2 CCAR1 DAZAP1 PUM3 DDX18 FUS PUS7 EIF3D ZC3H14 RBM3 HNRNPUL1 MRPL27 EFTUD2 RPL24 RBM25 G3BP2 EIF3H ZFP36L2 LARP4 RPL29 HNRNPF TMSB4X NELFE DDX3X NAP1L4 HNRNPH1 MRPS18A SFSWAP SMARCE1 PHAX
Single-stranded RNA binding	DAZAP1 ZC3H14 LARP4 HNRNPF DDX3X HNRNPH1
Poly-purine tract binding	DAZAP1 ZC3H14 LARP4 DDX3X
Nucleic acid binding	BCLAF1 STAU2 CCAR1 DAZAP1 SMARCE1 PUM3 DDX18 FUS PUS7 EIF3D ZC3H14 RBM3 HNRNPUL1 MRPL27 EFTUD2 RPL24 RBM25 G3BP2 EIF3H ZFP36L2 LARP4 RPL29 HNRNPF TMSB4X NELFE DDX3X GATAD2B NAP1L4 HNRNPH1 MRPS18A SMAD3 SFSWAP PHAX SNRNP27
Poly(A) binding	ZC3H14 LARP4 DDX3X
Isomerase activity	IDI1 PUS7 GLCE GNE PPIH HMGCS1
Cadherin binding	MICALL1 RPL24 USP8 TMOD3 STX5 RPL29 MRTFB DDX3X
Ubiquitin protein ligase binding	GABARAPL2 GABARAPL1 ATXN3 DNAJA1 NDUFS2 SMAD3 DAXX
Ubiquitin binding	BUB3 NEDD4 SMAD3 TAB2
Nuclear receptor binding	SMARCE1 FUS SMAD3 DAXX
Tat protein binding	DNAJA1 GABARAPL1
RNA polymerase II-specific DNA-binding transcription factor binding	SMARCE1 FUS DNAJA1 GABARAPL1 SMAD3 DAXX
RNA stem-loop binding	DDX3X DAZAP1
Transcription coactivator activity	CCAR1 MRTFB FUS DAXX NCOA4 SMARCE1

Columns (left to right) show the pathways and proteins involved in the given pathway in TM subjected to CMS, based on molecular function using ShinyGO pathway enrichment analysis.

**FIGURE 2:**
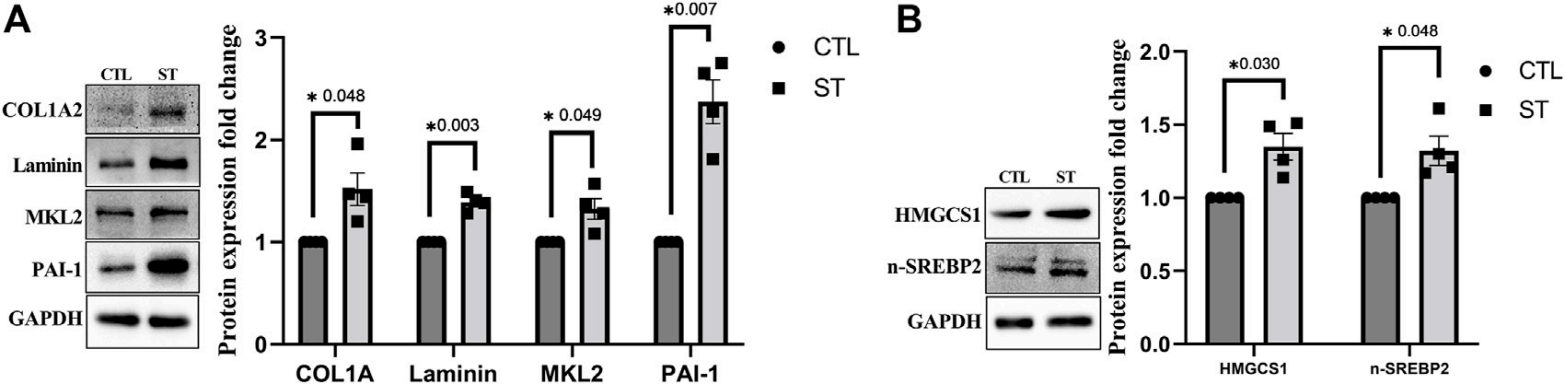
Immunoblot analysis for ECM and fibrosis-related proteins and proteins involved in cholesterol biosynthesis. **(A)** Confirmatory analysis for protein expression changes in HTM cells subjected to CMS for 24 h. COL1A2, total laminin, MKL2, and PAI-1 showed a significant upregulation at 24 h of CMS (ST) compared to unstretched control (CTL). **(B)** Confirmatory analysis for proteins involved in cholesterol biosynthesis in HTM cells subjected to CMS for 24 h. HMGCS1 and n-SREBP2 showed a significant upregulation at 24 h of CMS (ST) compared to unstretched control (CTL). The results were based on immunoblot analysis with subsequent densitometric analysis. GAPDH was immunoblotted as the loading control. Circles and boxes in the histograms represent sample numbers for CTL and ST, respectively. Values represent mean ± SEM, where *n* = 4 (biological replicates). *p ≤ 0.050 was considered significant.

We further performed a confirmatory immunoblotting analysis of TM cells subjected to CMS for selected proteins upregulated in proteomics. Figure 2A shows significant increase in the expression of ECM proteins such as COL1A2 (*n* = 4, *p* = 0.0048) and total laminin (*n* = 4, *p* = 0.003) and MKL2 (*n* = 4, *p* = 0.049), a coactivator of SRF and a regulator of actin polymerization, in TM cells subjected to CMS (ST) compared to control (CTL). Though there was no significant change in plasminogen activator inhibitor-1 (PAI-1) expression in proteomic analysis, we found it to be significantly increasing (*n* = 4, *p* = 0.007) in immunoblotting (Figure 2A), suggesting an increase in fibrogenic response (Ghosh and Vaughan, 2012) in TM subjected to CMS. Additionally, investigating cholesterol biosynthesis, our data from immunoblotting support the proteomics data in demonstrating a significant increase in HMGCS1 (*n* = 4, *p* = 0.030) (Figure 2B). The production of HMGCS is controlled by the activation of a transcription factor sterol regulatory element-binding protein 2 (SREBP2), a master regulator of cholesterol biosynthesis (Madison, 2016). Upon activation, SREBP traffics from ER to Golgi and gets cleaved, and the cleaved N-terminal protein moves into the nucleus to initiate the transcription by binding to the sterol response element (SRE) (Madison, 2016). In TM subjected to CMS, on examining the active form of SREBP2 (nSREBP2) by immunoblotting, we identified a significant increase (*n* = 4, *p* = 0.048) (Figure 2B), indicating a direct relationship between mechanical stress on inducing cholesterol synthesis.

Among the downregulated proteins, the coiled-coil domain-containing proteins, namely, CCDC113 (−0.95-log_2_FC; 0.52-FC) and CCDC170 (−0.91-log_2_FC; 0.53-FC), a component of centriole satellite and protein involved in microtubule organization and stabilization, respectively, were the most downregulated (Table 7). Intriguingly, CCDC113 was downregulated at mRNA and protein levels.

**TABLE 7:** Top 20 downregulated cellular proteins.

Gene symbol	Accession	Description	Fold change	log_2_FC	*p*-value
CCDC113	Q9H0I3	Coiled-coil domain-containing protein 113	0.518	−0.950	0.009
CCDC170	Q8IYT3	Coiled-coil domain-containing protein 170	0.53	−0.910	0.028
RASSF3	Q86WH2	Ras association domain-containing protein 3	0.556	−0.850	0.042
CASP8AP2	Q9UKL3	CASP8-associated protein 2	0.585	−0.770	0.035
SYNE2	Q8WXH0	Nesprin-2	0.588	−0.770	0.037
DOCK2	Q92608	Dedicator of cytokinesis protein 2	0.59	−0.760	0.026
LRRC7	Q96NW7	Leucine-rich repeat-containing protein 7	0.643	−0.640	0.023
HYDIN	Q4G0P3	Hydrocephalus-inducing protein homolog	0.676	−0.570	0.040
NDE1	Q9NXR1	Nuclear distribution protein nudE homolog 1	0.681	−0.550	0.016
MYH4	Q9Y623	Myosin-4	0.682	−0.550	0.017
NRXN3	Q9Y4C0	Neurexin-3	0.687	−0.540	0.008
ZNF207	O43670	BUB3-interacting and GLEBS motif-containing protein ZNF207	0.704	−0.510	0.036
KEAP1	Q14145	Kelch-like ECH-associated protein 1	0.718	−0.480	0.042
CHD8	Q9HCK8	Chromodomain-helicase-DNA-binding protein 8	0.729	−0.460	0.015
FSHR	P23945	Follicle-stimulating hormone receptor	0.747	−0.420	0.022
AP3M2	P53677	AP-3 complex subunit mu-2	0.755	−0.410	0.040
PMM2	O15305	Phosphomannomutase 2	0.756	−0.400	0.023
PCBP3	P57721	Poly(rC)-binding protein 3	0.765	−0.390	0.007
ATG13	O75143	Autophagy-related protein 13	0.766	−0.390	0.036
TMEM132B	Q14DG7	Transmembrane protein 132B	0.766	−0.380	0.035

Top 20 proteins significantly downregulated in TM subjected to CMS with FDR ≤ 5% and log_2_FC ≤ -0.3. Columns (left to right) show the gene symbol, UniProt accession number, protein description, fold change, log_2_FC determined by global proteomic analysis, and calculated *p* value, where *p* < 0.050 was statistically significant.

Collagen alpha-2(IV) chain (COL4A2), one of the most abundant type IV collagens involved in the formation of the basement membrane, was downregulated (−0.15-log_2_FC; 0.90-FC). Actin binding Nesprin-2 (−0.77-log_2_FC; 0.59-FC) (Dawe et al., 2009) and the dedicator of cytokinesis protein 2 (DOCK2) (−0.76-log2FC; 0.59-FC), which is involved in activating Rho family of GTPases (Laurin and Côté, 2014), were downregulated significantly. Pro-apoptotic protein, CASP8-associated protein 2 (CASP8AP2), was downregulated (−0.77-log_2_FC; 0.59-FC). Though proteins involved in autophagosome formation and maturation were upregulated, we found that autophagy-related protein 13 (ATG13), essential for autophagy induction and autophagosome formation (Behrends et al., 2010), was significantly downregulated (−0.39-log_2_FC; 0.77-FC). Thus, indicating a potential activation and a balancing act on the autophagic mechanisms under mechanical stress in the TM cells. Pathway enrichment analysis performed using ShinyGO v0.75 (Ge et al., 2020) tool for downregulated proteins based on molecular function (Table 8) showed several proteins associated with biological quality, cellular localization, and stress response. Network analysis of downregulated proteins is shown in Supplementary Figure S6.

**TABLE 8:** Pathway enrichment analysis for downregulated cellular proteins using ShinyGO based on molecular function.

High level GO category	Proteins
Regulation of biological quality	GRIPAP1 KEAP1 F10 BDH2 STIM1 DDAH1 FSHR APRT ZNF207 TNIP2 NMT1
Cellular localization	ATG13 GRIPAP1 NDE1 SYNE2 AP3M2 DOCK2 HEL-S-163pA F10 NMT1 APRT
Response to stress	F10 UBE4B BRCC3 KEAP1 DDAH1 TFEC TNIP2 ATG13 FSHR
Response to external stimulus	CHD8 CMPK2 DOCK2 TNIP2 NRXN3 F10 NMT1 FSHR
Regulation of molecular function	KEAP1 STIM1 FSHR PHACTR3 DOCK2 BRCC3 NDE1 DDAH1
Regulation of localization	ATG13 GRIPAP1 STIM1 SYNE2 DOCK2 F10 NMT1
System process	DDAH1 MYH4 CHD8 FSHR NRXN3 STIM1
Catabolic process	UBE4B ATG13 KEAP1 DDAH1 BDH2 PRELP
Regulation of signaling	TNIP2 CHD8 F10 FSHR NMT1 STIM1
Cellular component biogenesis	ZNF207 CCDC113 ATG13 NDE1 SYNE2 MRM3
Regulation of multicellular organismal process	COL4A2 KEAP1 DDAH1 FSHR STIM1 F10
Immune system process	DOCK2 FSHR TNIP2 HEL-S-163pA APRT

Columns (left to right) show the pathways and proteins involved in the given pathway in TM subjected to CMS, based on molecular function using ShinyGO pathway enrichment analysis.

### Lipid changes in TM subjected to CMS

Lipidomics was performed in control and stretched HTM cells from cellular lipids and conditioned media.

The PCA for the following lipid classes yielded two distinct clusters between control and stretched HTM cells in total phospholipids (Supplementary Figure S7A), total ceramide (Cer) (Supplementary Figure S7B), total cholesteryl ester (Supplementary Figure S7C), and total diacylglycerol (DG) (Supplementary Figure S7D). The first and second principal components, PC1 and PC2, explained >50% of the variance, indicating that total phospholipids, Cer, cholesteryl esters, and DG were highly different between control and stretched HTM cells. The PCA for total TAG showed no distinct cluster between control and stretched HTM cells (Supplementary Figure S7E).

In the lipids from conditioned media, the PCA revealed two distinct clusters of total phospholipids in control and stretched HTM conditioned media (Supplementary Figure S7F). The first two principal components explained >50% of the variance, indicating that total phospholipids differed highly between control and stretched HTM conditioned media (Supplementary Figure S7F). However, in PCA for the lysophospholipids, there was no distinct grouping in control and stretched HTM conditioned media (Supplementary Figure S7G).

**FIGURE 3:**
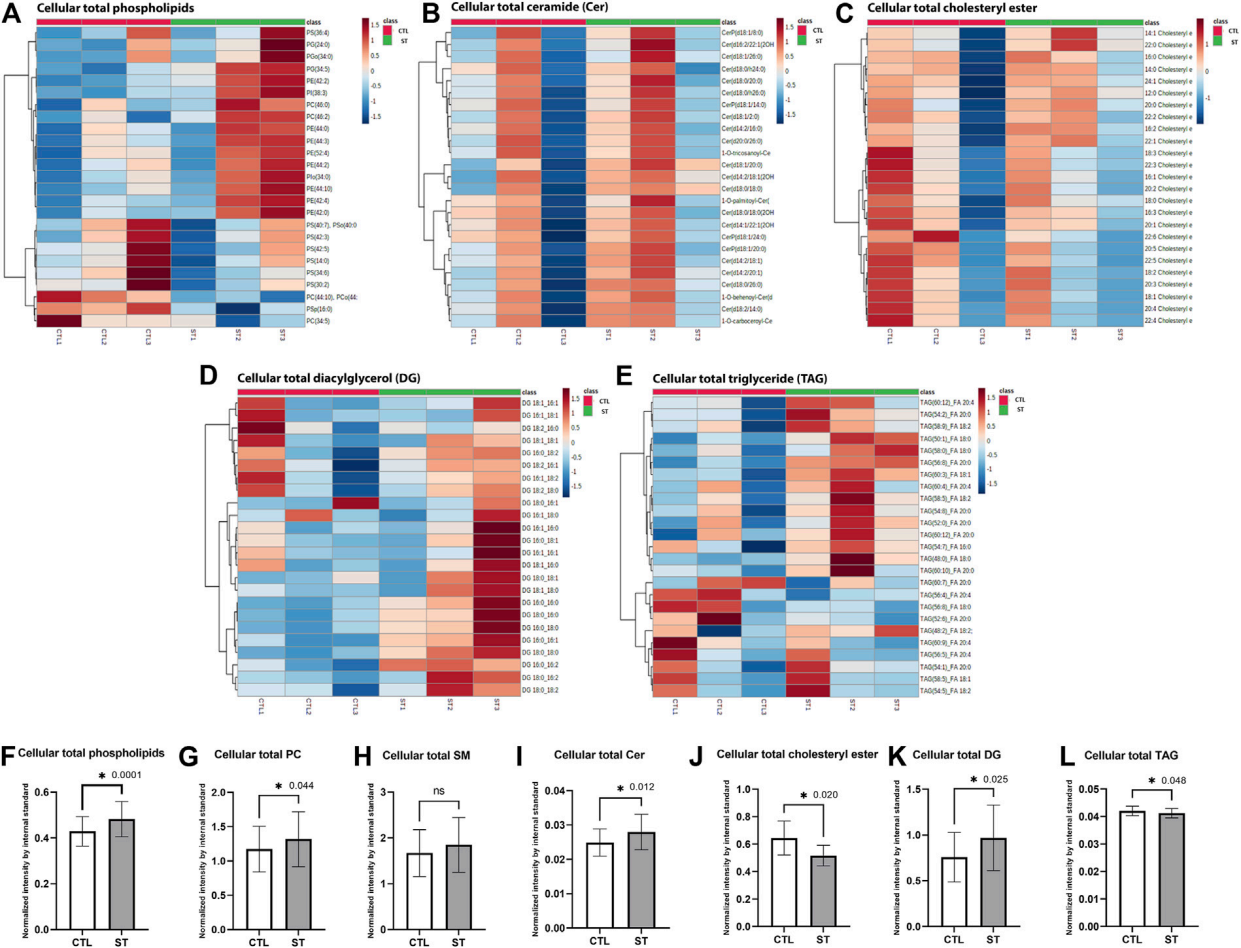
Cellular lipid analysis. **(A)** Heatmap of the total cellular phospholipids in control and stretched HTM cells. **(B)** Heatmap of the total cellular ceramide (Cer) in control and stretched HTM cells. **(C)** Heatmap of the total cellular cholesteryl ester in control and stretched HTM cells. **(D)** Heatmap of the total cellular diacylglycerol (DG) in control and stretched HTM cells. **(E)** Heatmap of the total cellular triglyceride (TAG) in control and stretched HTM cells. All heatmaps are presented by control (left 3 red columns) and stretched HTM cells (right green columns), total *n* = 3 pairs (biological replicates). Analysis parameters utilized the Ward clustering algorithm, Euclidean distance measure, and autoscaling based upon samples (performed using MetaboAnlayst 5.0). Comparison between control and stretched HTM cells. **(F)** Total cellular phospholipids were significantly increased in stretched HTM cells. **(G)** Total cellular phosphatidylcholine (PC) was significantly increased in stretched HTM cells. **(H)** Total cellular sphingomyelin (SM) was increased in stretched HTM cells but was nonsignificant. **(I)** Total cellular Cer was significantly increased in stretched HTM cells. **(J)** Total cellular cholesteryl ester was significantly decreased in stretched HTM cells. **(K)** Total cellular DG was significantly increased in stretched HTM cells. **(L)** Total cellular TAG was significantly decreased in stretched HTM cells. Values represent the mean ± SEM, where *n* = 3 (biological replicates). *p ≤ 0.050 was considered significant.

Further analysis of each class of lipids is shown as follows:

(1) Total 603 phospholipid classes, including phosphatidylcholine (PC), phosphatidylinositol (PI), phosphatidylethanolamine (PE), phosphatidylserine (PS), phosphatidylglycerol (PG), and sphingomyelin (SM), were identified. Cluster heatmap of the top 25 phospholipids showed that most were increased in stretched HTM cells compared to the control (Figure 3A). Paired *t*-test showed that there was a significant increase in total phospholipids (*p* = 0.0001) in stretched HTM cells compared to control (Figure 3F).
(2) Among all phospholipids, 101 PC classes were identified. PC is an abundant component in the cell membrane (Kanno et al., 2007). Therefore, we next measured the change of total PC level in phospholipids of control and stretched HTM cells. The paired *t*-test showed that total PC was significantly increased (*p* = 0.044) in stretched HTM cells compared to control (Figure 3G). In addition to PC, we also compared the changes of PI, PE, PG, and PS in control and stretched HTM cells. A total of 133 PI classes were identified. The paired *t*-test showed that total PI was significantly increased (*p* = 0.0001) in stretched HTM cells (Supplementary Figure S8A). A total of 141 PE classes were identified, and the paired *t*-test showed that total PE was significantly increased (*p* = 0.0002) in stretched HTM cells (Supplementary Figure S8B). A total of 134 PG classes were identified in lipidomics analysis, and the paired *t*-test showed that total PG was significantly increased (*p* = 0.007) in stretched HTM cells (Supplementary Figure S8C). A total of 131 PS classes were identified, and the paired *t*-test showed no difference in the total PS level between control and stretched HTM cells (Supplementary Figure S8D).
(3) Since sphingosine-1-phosphate (S1P) is known to regulate IOP (Mettu et al., 2004; Stamer et al., 2009; Sumida and Stamer, 2010, 2011; Honjo et al., 2018; Ho et al., 2020), we next checked the phospholipid sphingomyelin (SM) level, a source of S1P synthesis, in control and stretched HTM cells. Among all phospholipids, 27 SM classes were identified. We also found an increase in SM with no significance in stretched HTM cells compared to control (Figure 3H).
(4) In addition to SM, we also checked the levels of Cer, another lipid involved in S1P synthesis in the control and stretched HTM cells. A total of 80 Cer classes were identified. The cluster heatmap showed the top 25 upregulated Cer in stretched HTM cells compared to control (Figure 3B), and the paired *t*-test for the total Cer resulted in significantly increased Cer levels (*p* = 0.012) in stretched HTM cells (Figure 3I). Such increases in SM and Cer indicate a potential increase in the biogenesis of S1P in stretched HTM cells.
(5) A total of 25 cholesteryl ester classes were identified. The cluster heatmap showed that cholesteryl esters were decreased in two stretched HTM cell lines and increased in one line (Figure 3C). The paired *t*-test showed that the total cholesteryl ester significantly decreased (*p* = 0.02) in stretched HTM cells compared to the control (Figure 3J).
(6) A total of 24 diacylglycerol (DG) classes were identified. Heatmap from cluster analysis showed that total DG was increased in stretched HTM cell samples (Figure 3D), and in accordance with this observation, the paired *t*-test also showed that total DG was significantly increased (*p* = 0.025) in stretched HTM cells compared to the control (Figure 3K).
(7) A total of 328 triglyceride (TAG) classes were identified. The cluster heatmap of the top 25 TAGs showed that some were increased, and some were decreased in stretched HTM cells (Figure 3E). The paired *t*-test showed that total TAG levels were significantly decreased (*p* = 0.048) in stretched HTM cells compared to the controls (Figure 3L).

**FIGURE 4:**
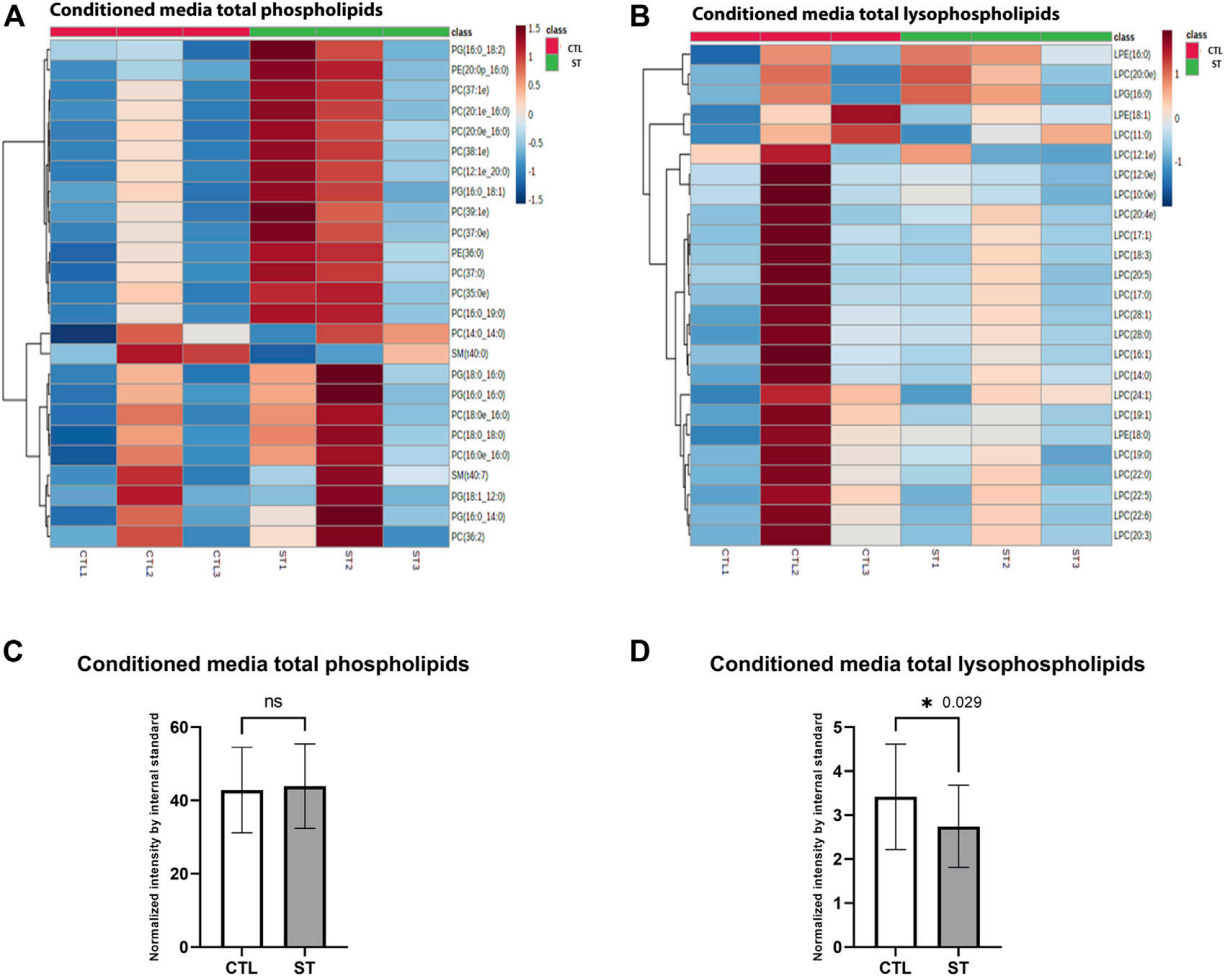
Secretory lipid analysis. **(A)** Heatmap of the total media phospholipids in control and stretched HTM cells. **(B)** Heatmap of the total media lysophospholipids in control and stretched HTM cells. Comparison between control and stretched HTM cell culture media. **(C)** Total media phospholipids were increased in stretched HTM cell culture media but were nonsignificant. **(D)** Total media lysophospholipids were significantly decreased in HTM cell culture media. Values represent the mean ± SEM, where *n* = 3 (biological replicates). *p ≤ 0.050 was considered significant.

In addition to the analysis of cellular lipids, we also evaluated lipid changes in the conditioned media obtained from control and stretched HTM. Since lysophosphatidic acid (Liu et al., 2018) is an important extracellular lipid growth factor involved in the regulation of TM cell function and IOP (Mettu et al., 2004; Honjo et al., 2018; Ho et al., 2020), we analyzed the changes in total phospholipids (PC, PI, PE, PS, PG, and SM) and lysophospholipids (lysophosphatidylcholine, lysophosphatidylethanolamine, and lysophosphatidylglycerol) level in conditioned media, which are the resources of extracellular LPA. Lipidomics analysis identified 384 phospholipid and 43 lysophospholipid classes in control and stretched HTM conditioned media. Cluster heatmap of the top 25 phospholipids showed that most were increased in stretched HTM conditioned media compared to control (Figure 4A), and paired *t*-test showed a small increase in total phospholipids level in stretched HTM conditioned media (Figure 4C). Cluster heatmap of the top 25 lysophospholipids showed that they were decreased in stretched HTM conditioned media compared to the control (Figure 4B), and paired *t*-test also presented a significant decrease in total lysophospholipids (*p* = 0.029) in stretched HTM conditioned media (Figure 4D).

The lipidomics analysis revealed interesting changes in lipid classes in TM experiencing CMS, indicating an active remodeling of lipid components in the TM. The intracellular and extracellular lipid changes, such as Cer, SM, and lysophospholipids, can promote S1P and LPA biosynthesis, further regulating HTM cell biomechanics and TM stiffness.

## Discussion

To date, two studies have utilized the “omics” approach to identify changes in genes and pathways in TM subjected to CMS. Luna et al. (2009) used microarray analysis to find the differentially expressed genes, and a more recent study (Youngblood et al., 2020) utilized RNA sequencing to identify mRNAs, micro RNAs, and long non-coding RNAs. Interestingly, a comparative analysis of our study where we performed mRNA-seq with the study of Youngblood et al. (2020) that utilized total RNA-seq analysis identified a similar upregulation of genes such as MARCKS like 1 (MARCKSL1), leucine-rich adaptor protein 1 like (LURAPIL), SLC38A2, a regulator of chromosome condensation 1 (RCC1), SLC5A3, CEP295 N-terminal like (CEP295NL), and downregulation of thioredoxin interacting protein (TXNIP).

To better understand and integrate the stress-responsive mechanisms utilized by TM due to mechanical stress, in this exploratory study, we have performed an unbiased multiomics analysis to identify links between changes in mRNA, proteins, and lipids using RNA sequencing, proteomics, and lipidomics on TM cells subjected to CMS. Our study reveals that mechanical stress induced a potential protein quality control response to counter oxidative stress and induction of cellular contractility and cell adhesive interactions combined with pro-fibrogenic signaling *via* increased lipid signaling and metabolic response. In addition, we find that the cells turn on various homeostatic control signaling machinery to counteract such stress.

### TM counters CMS-induced oxidative stress and apoptosis

Based on the RNA-seq analysis, our study found 211 differentially expressed genes. Many genes found to be differentially expressed in our study belonged to stress-response, protein ubiquitination, transcription repression, and chaperone activity. Understanding the role of oxidative stress and reactive oxygen species is of significant interest in the pathogenesis of ocular hypertension and glaucoma. Multiple lines of investigations pointed out the relationship between oxidative stress-related damage to TM and elevated IOP (Saccà and Izzotti, 2008; Saccà et al., 2016; Tanito et al., 2016; Wu et al., 2022). First, we propose that CMS induces oxidative stress on the TM and activates transcription and translation to negate oxidative stress. Our RNA-seq analysis revealed that downstream response genes under oxidative stress, including HSPA1B and HES1, were upregulated, whereas CCL2, TXNIP, and MEKK6/MAP3K6 were downregulated. An earlier study also indicated the induction of oxidative stress in TM upon stretching (Vittal et al., 2005). Our proteomics data support the potential negative regulation of oxidative stress by the downregulation of the Kelch-like ECH-associated protein 1 (KEAP1). Mechanistically, nuclear factor erythroid 2- (NFE2-) related factor 2 (NRF2) is a regulator of cellular resistance to oxidative stress, and under basal conditions, NRF2 is suppressed by binding to the repressor protein KEAP1. During oxidative stress, NRF2 becomes active by dissociating from KEAP1 (Deshmukh et al., 2017). In addition, we found upregulation of casein kinase 2α (CSNK2A2), which is required for the phosphorylation of NRF2, which aids in the translocation of NRF2 to the nucleus and activates several cytoprotective genes (Apopa et al., 2008). Thus, the negative regulation of KEAP1 in TM indicates a potential activation of NRF2 because of the likely oxidative stress damage of TM subjected to CMS. Our study also points out that the loss of counter-mechanisms to oxidative stress in TM can be an important step in ocular hypertension and glaucoma pathogenesis. Interestingly, TXNIP and MAP3K6 are pro-apoptotic genes, and the decrease in their transcription aid in protecting the cells from undergoing cell death (Takeda et al., 2007; Li J. et al., 2017). We observed that proteins involved in inhibition of apoptosis were increased in TM undergoing CMS. Upregulation of DnaJ homolog subfamily A member 1 (DNAJA1), a co-chaperone for HSPA8/Hsc70 prevents BAX translocation to mitochondria, can inhibit apoptosis (Gotoh et al., 2004); death domain-associated protein 6 (DAXX), a transcription repressor is a negative regulator of p53-mediated apoptosis (Zhao et al., 2004; McDonough et al., 2009); and tax1-binding protein 1 (TAX1BP1) is an inhibitor of TNF-induced apoptosis (Shembade et al., 2007). Incidentally, DNAJA1 was upregulated at transcript and protein levels, signifying its effects on regulating chaperone function and inhibiting apoptosis. In contrast, CASP8AP2, which is required for FAS-mediated apoptosis, was downregulated. Even in our mRNA sequencing data, we found that TXNIP, which is known to be involved in apoptosis (Li J. et al., 2017), was going down. We believe these apoptotic inhibitory aspects in TM cells could be a cell survival mechanism to overcome mechanical stress. The potential role of oxidative stress-induced DNA damage can upregulate the expression of DNA repair enzymes (Bridge et al., 2014). In our RNA-seq analyses, the top upregulated gene was MSH4, which is involved in meiotic recombination (Snowden et al., 2004) during DNA repair (Tock and Henderson, 2018), indicating potential protection against oxidative DNA damage. MSH4 also interacts with von Hippel-Lindau binding protein 1 (VBP1), which binds to VHL ubiquitin E3 ligase that regulates cellular response to oxidative stress, functioning in the protein degradation pathway (Xu and Her, 2013).

### TM overcomes mechanical stress by upregulating transcriptional and translational control of autophagy and protein quality control

Autophagy is an adaptive response mechanism for the survival of cells experiencing stress. Autophagy induction in TM subjected to CMS was previously reported (Shim et al., 2020). In our study, we found the upregulation of HSPB8 mRNA, which promotes autophagosome formation (Li X. C. et al., 2017), and an increase in NRBF2 and GABARAPL2 proteins, which are essential for the formation and maturation of autophagosome, respectively (Behrends et al., 2010). Hence, the increase in proteins related to autophagy can potentially aid the TM cells to combat stress (Rao et al., 2021) and modulate the fibrogenic pathway in TM (Nettesheim et al., 2019), and an increase in autophagic process/response serves as an anti-apoptotic signal (Liu et al., 2015). Protein folding mechanisms can be compromised in cells under stress due to mechanical insults (Höhfeld et al., 2021). Such misfolded proteins are cleared by the process of ubiquitination. Our study showed the induction of various HSP genes and TNF receptor-associated factor 1(TRAF1) involved in the regulation of proteostasis. In addition, we found upregulation of TAB2 and downregulation of TNFAIP3-interacting protein 2 (TNIP2), which are involved in protein quality control mechanism *via* the NFκB pathway (Kanayama et al., 2004; Banks et al., 2016). The NFκB pathway plays an important role in protein clearance by regulating autophagy (Nivon et al., 2016) and is known to regulate TGF-β2-mediated ocular hypertension (Hernandez et al., 2020).

Lipids are involved in the autophagy pathway (Soto-Avellaneda and Morrison, 2020). Phosphoinositides, a class of phospholipids derived from PI, play a major role in the regulation of autophagy. They can control the pathway that directly activates or deactivates mTORC1, which controls the balance between growth and autophagy (Soto-Avellaneda and Morrison, 2020). Generally, mTORC1 is active in the presence of nutrients, driving the growth and anabolic process while suppressing autophagy. In the absence/depletion of nutrients, mTORC1 is inactivated, and autophagy is promoted. We speculate that CMS on TM promotes nutrient utilization to meet energy expenditure. Since the cells are stretched in the conditioned media for 24 h, nutrients are used up from the conditioned media and leading to the depletion of some nutrients. In addition to PI, in the absence/depletion of nutrients, PC and its product phosphatidic acid (PA), as well as DG, can promote autophagy by mediating signaling and membrane remodeling to support autophagy activation. The Cer and its product S1P can contribute to mTORC1 inhibition and promote autophagy (Dall'Armi et al., 2013). In our lipidomics analysis, we found significantly increased PI, PC, DG, and Cer in stretched HTM cells implying the balance between growth and autophagy under CMS. On the contrary, we observed increased SREBP2 activation and lipid biogenesis in stretched HTM cells, which can be downstream of mTORC1 signaling (Lewis et al., 2011; Peterson et al., 2011). All these lipid changes imply that the HTM cell is achieving a balance between growth and autophagy under mechanical stress.

### Induction of actin-based contraction and ECM buildup by the interplay of the gene, protein, and lipid signaling in TM undergoing CMS

Cells respond to various signals, especially mechanical stimuli, through changes in genes and proteins associated with actin-cytoskeleton and ECM interactions (Martino et al., 2018). Not surprisingly, this study identified the upregulation of actin-cytoskeleton-associated genes, including SRPGAP1, SPTBN5, FMNL2, Ral A, and CHORDC1, and the downregulation of RIPOR2/RIPR2 under CMS. Interestingly, CHORDC1/CHP1, a negative regulator of Rho GTPase signaling by inhibiting Rho kinase (Ferretti et al., 2010), acts as a circuit breaker of Rho-Rho kinase signaling, indicating the potential homeostatic effect on Rho GTPase-mediated mechanotransduction. Looking at the regulation of protein, we found upregulation of cytoskeletal-associated proteins such as a) ataxin-3 involved in the maintenance of actin, b) MKL2, a transcriptional coregulator of SRF, c) thymosin beta 4, which is involved in sequestering of g-actin, and d) tropomodulin-3 that is involved in actin stabilization and downregulation of DOCK2, which can activate Rac GTPases (Sanui et al., 2003), and Nesprin-2, which modulates mechanotransduction between the cytoplasm and nuclear lamina (Stewart-Hutchinson et al., 2008). Of importance is the upregulation of MKL2, which is involved in the regulation of actin cytoskeletal organization and fibrosis in HTM cells through the transcriptional control of SRF-mediated actin polymerization (Pattabiraman and Rao, 2010; Pattabiraman et al., 2014). Thymosin beta 4 and tropomodulin-3 increase in TM under experimental IOP elevation (Gonzalez et al., 2000; Borrás, 2003; Vittitow and Borrás, 2004). It is interesting to note that the most upregulated protein is the methyltransferase-like protein 13, catalyzing the methylation of elongation factor 1-alpha (eEF1A) (Jakobsson et al., 2018). eEF1A has been reported to be colocalized with F-actin and associated with changes in the cytoskeletal organization (Liu et al., 1996). In gene and protein expression analysis, the most downregulated are coiled-coil domain-containing proteins, which are involved in microtubule binding, organization, and stabilization (Pal et al., 2010; Kumari and Panda, 2018). Microtubules are reported to self-repair in response to mechanical stress (Schaedel et al., 2015). It has been reported that microtubule disruption leads to a cellular contraction in human TM cells in response to stimuli (Gills et al., 1998). We speculate that the downregulation of proteins related to microtubule organization can potentially disrupt microtubule assembly or act as a response mechanism to mechanical stress on TM. The major lipid growth factors, including LPA and S1P, are known to regulate actin cytoskeleton and barrier function in endothelial cells and function in TM by binding their cognate receptors to regulate IOP (Mettu et al., 2004; Sumida and Stamer, 2011; Rao, 2014). The S1P is synthesized inside the cell, which is generated by phosphorylation of sphingosine through SphK (Fyrst and Saba, 2010). Sphingosine is derived by the hydrolysis of Cer during the sequential degradation of plasma membrane glycosphingolipids and SM (Proia and Hla, 2015). We found that SM and Cer increased in stretched HTM cells, and our mRNA sequencing analysis also found an increase in the expression of SphK in stretched HTM cells. These results indicate that, under CMS, HTM cells generate more substrates in the S1P biosynthesis pathway. Combined with increased SphK, more S1P will be produced inside the cells and exported into the extracellular environment. Outside the cell, S1P can bind to the S1P receptors (S1PR1-5) located on the cell membrane and cause a series of downstream effects (Proia and Hla, 2015). A speculative sequence of events in TM due to altered S1P and LPA identified in this study and previous studies are shown in Supplementary Figure S9. Additionally, in the extracellular space, phospholipids are converted to lysophospholipids by phospholipase A1/2, which is converted to LPA by the enzyme autotaxin (ATX) (Perrakis and Moolenaar, 2014). LPA can bind to their cognate LPA receptors, activate a series of downstream signaling cascades, and regulate HTM cell biomechanics (Supplementary Figure S9). Our lipidomics analysis demonstrated no significant difference in total phospholipid levels in conditioned media between control and stretched HTM. However, the total lysophospholipid levels were significantly decreased in stretched HTM conditioned media. We predict this is due to the dynamic conversion of lysophospholipid to LPA as previously reported that ATX and its activity are increased in stretched TM and the conditioned media (Iyer et al., 2012; Ho et al., 2018).

Mechanical stress positively regulates growth factor-induced fibrogenic activation in TM (Liton et al., 2005; Ashwinbalaji et al., 2020). Like earlier studies (Vittal et al., 2005; Luna et al., 2009), our analysis found regulation in the genes related to enzymes involved in ECM regulation—MMP16 (upregulated) and hyaluronidase-1(downregulated). The differences in the degree of changes in these studies could result from differences in experimental technique (microarray vs. mRNA-seq) and altered response by the donor tissue to prepare the HTM cells. We found a potential increase in fibrogenic activation with an increase in SMAD3 protein known to signal *via* the canonical TGF-β signaling pathway and PAI-1 levels in TM subjected to CMS (Flanders, 2004; Pattabiraman et al., 2014) and an increase in COL1A2 and total laminin proteins. Interestingly, the COL4A2 protein, one of the major structural components of the basement membrane, was downregulated, correlating with earlier microarray analysis (Luna et al., 2009).

The cellular phospholipids and cholesterol composition play an important role in the cell-ECM interactions (Márquez et al., 2008; Horn and Jaiswal, 2019), and their dysregulation can result in pathology as observed in lung fibrosis (Burgy et al., 2022) and renal pathology (Abrass, 2004). A previous study found that patients with glaucoma have higher cholesterol levels (Posch-Pertl et al., 2022), and patients with hyperlipidemia will have an increased risk of getting glaucoma (Wang and Bao, 2019). In addition, cholesterol-lowering statin, an inhibitor of HMG-CoA reductase, can decrease the risk of POAG and be an effective therapy for POAG (McGwin et al., 2004; Stein et al., 2012; Wu et al., 2020). Statins are proposed to decrease IOP by inhibiting the Rho and Rho-kinase activity in TM cells (Song et al., 2005; Von Zee et al., 2009; Cong et al., 2018). This inhibition can alter TM cell morphology and decrease actomyosin contractile activity and ECM assembly. The current understanding of how cholesterol changes in stretched HTM cells is still unclear. Due to poor ionization and the low amount of free cholesterol in the cell, detection of free cholesterol is difficult, and free cholesterol is usually detected by GC-MS (Edwards et al., 2011). We evaluated the cholesteryl ester level changes in stretched HTM cells. We found that cholesteryl ester was significantly decreased in stretched HTM cells. Interestingly, our proteomics and immunoblotting analysis found that HMGCS, a critical enzyme in the cholesterol biosynthesis pathway, was significantly increased in HTM cells. Moreover, immunoblotting of whole cell lysate post mechanical stretch, we found activation of SREBP2, a transcriptional factor promoting cholesterol biosynthesis (Madison, 2016). The decreased cholesteryl ester levels observed in stretched HTM cells are speculated to result from less stockpile of cholesterol inside the cell as it is used for rapid membrane changes and the release of free cholesterol from cholesteryl esters (van Meer, 2001; Walther and Farese, 2012). Low cholesterol levels act as a stimulus for SREBP activation (Madison, 2016), which can turn on more cholesterol synthesis. These results put together indicate an increased cholesterol biosynthesis in stretched HTM cells.

DG is an intracellular lipid second messenger involved in multiple processes and pathways. Cellular DG can be synthesized from TAG and phospholipid (Carrasco and Mérida, 2007; Fagone and Jackowski, 2009; Coleman and Mashek, 2011). Cellular DG can directly interact with membrane-bound PKC and lead to its activation. The role of PKC in TM has been studied before. The DG analog diC8 can stimulate membrane-bound PKC and further cause increased TM cell contraction (Thieme et al., 1999). PKC activators such as phorbol-12-myristate 13-acetate (PMA) and phorbol-12,13-dibutyrate (PDBu) increased the formation of actin stress fibers and focal adhesions and myosin light chain (MLC) phosphorylation in TM cells (Khurana et al., 2003). In contrast, PKC inhibitors such as H7 or chelerythrine can induce relaxation in TM (Wiederholt et al., 1998; Thieme et al., 1999). These inhibitors have been used successfully to lower IOP in the animal model (Tian et al., 1999). In our lipidomics analysis, we found that both DG and its substrate—phospholipid—are increased in stretched HTM cells, indicating that under CMS, more DG generated can result in the activation of PKC and further cause increased TM cell contraction.

#### Interplay between mRNA, protein, and lipid pathway changes in response to CMS

**FIGURE 5:**
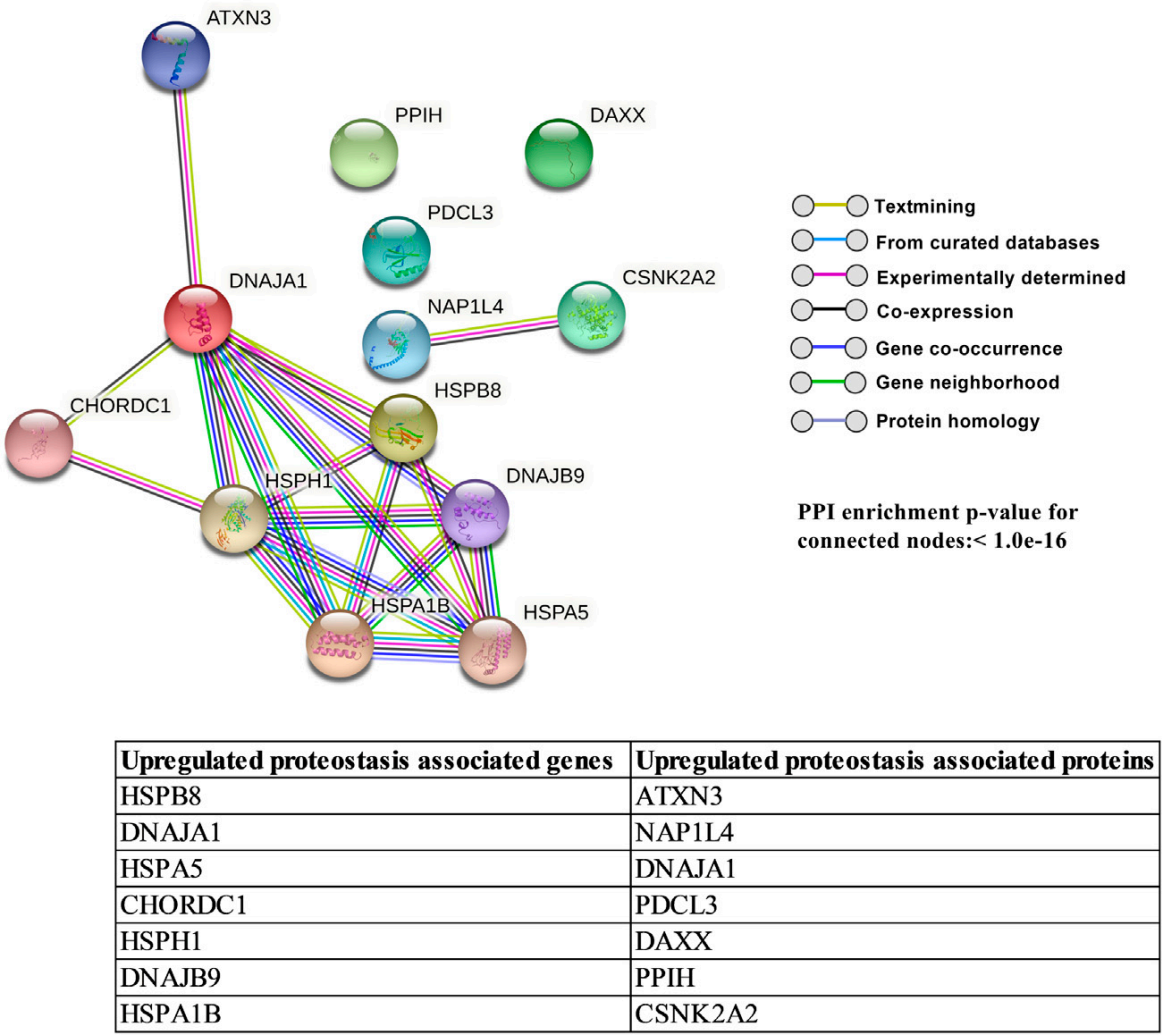
STRING network analysis of upregulated proteostasis-associated genes and proteins. Colored lines between the nodes indicate their basis for interconnection. Table here gives a list of inclusive genes and proteins. Overall, the protein–protein interaction (PPI) enrichment *p*-value is shown for connected nodes and is considered significant if *p* < 0.05 with individual PPI interaction significance not shown.

**FIGURE 6:**
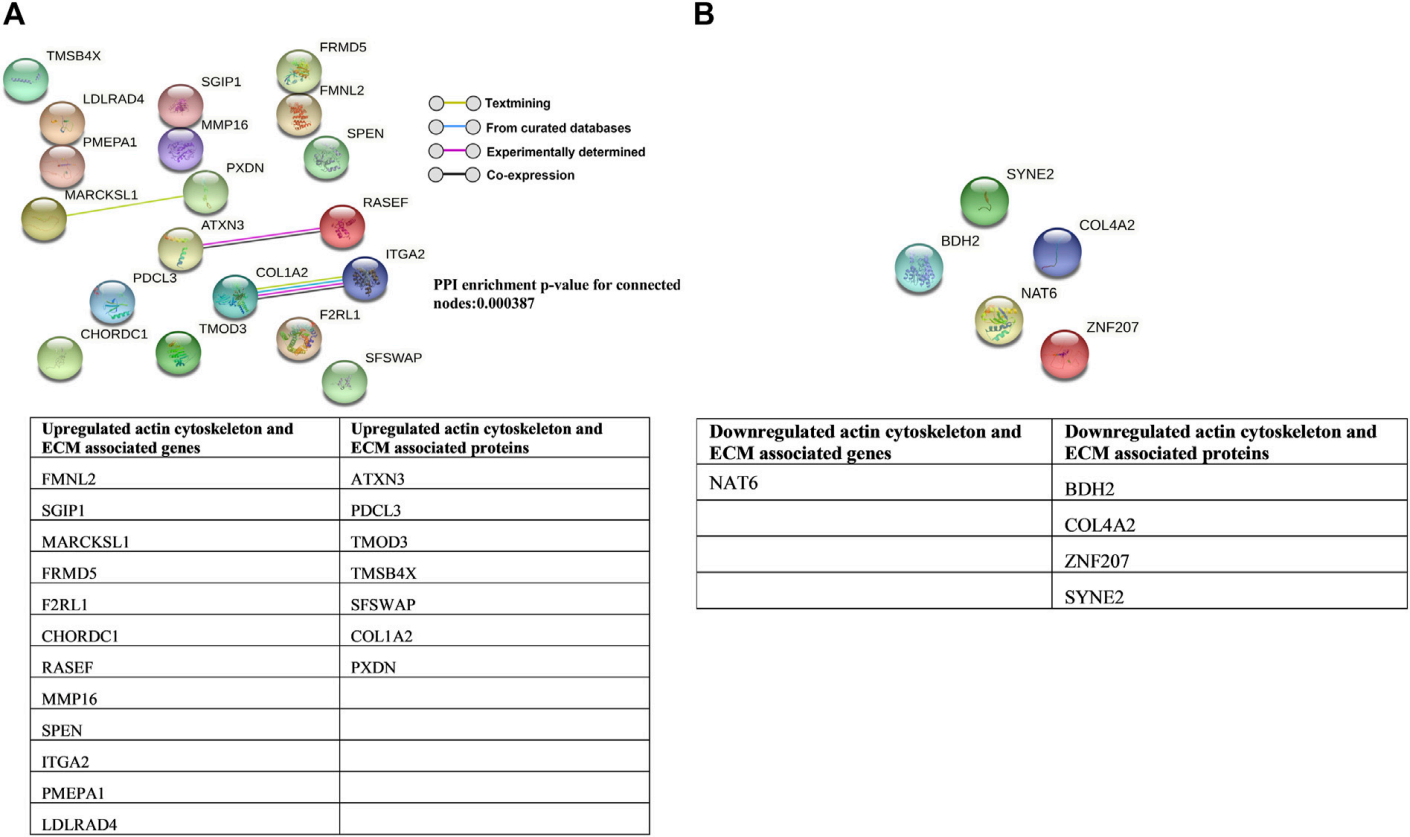
STRING network analysis of actin cytoskeleton and ECM-associated genes and proteins. **(A)** Network analysis of all upregulated actin cytoskeleton and ECM-associated genes and proteins. **(B)** Network analysis of all downregulated actin cytoskeleton and ECM-associated genes and proteins. Table here gives a list of inclusive genes and proteins. Colored lines between the nodes indicate their basis for interconnection. Overall PPI enrichment *p*-value is shown for connected nodes and is considered significant if *p* < 0.05 with individual PPI interaction significance not shown.

Though there is no direct method to derive the interplay between mRNA, protein, and lipid pathways due to CMS, to create a conceptual interactome, we utilized the STRING database and analyzed the interactions and interaction strength between molecules either upregulated or downregulated within pathways. Interestingly, we found strong and significant interactions in the genes and proteins upregulated in association with proteostasis (Figure 5), actin cytoskeleton and ECM-associated pathway (Figure 6), and apoptosis pathway (Supplementary Figure S10) and downregulated in association with lipid metabolism (Supplementary Figure S11). Other network analysis of the downregulated oxidative stress-related genes and proteins (Supplementary Figure S12), downregulated apoptosis-associated proteins (Supplementary Figure S13), upregulation and downregulation of autophagy-related proteins (Supplementary Figures S14, S15), and upregulated lipid-related genes and proteins (Supplementary Figure S16) showed minimal interactors.

One limitation to the study is the choice of a low cut-off for significance, which was not arbitrary. We believe that even a small fold change in proteins can yield functional significance. In signal transduction, stoichiometry is extremely important because enzymes, repressors, and activators, among others, directly induce signal transduction cascades (Lee and Yaffe, 2016). Also, because we have investigated the changes in RNA for 8 h and proteins and lipids for 24 h, we believe that such changes can also include the consequence of a homeostatic mechanism. Another limitation is only performing proteomics and lipidomics analysis on the cellular proteins and lipids, respectively, because the secreted biomolecules also play a significant role in the outside-in signaling. Further studies on the secreted proteins and the investigation into cell-based and animal model-based studies can reveal the significance of such interaction in TM physiology and their potential pathological relevance in ocular hypertension.

### Disease relevance of RNA, proteins, and lipids modulated in TM under CMS

Some of the genes, proteins, and lipid pathways we report here are associated with systemic hypertension, elevated IOP, and POAG pathogenesis. Comparing the GWAS catalog (https://www.ebi.ac.uk/gwas/home) under open-angle glaucoma trait with the differentially regulated genes and proteins found in our analysis, we found nearly 15 genes and protein changes. The upregulated genes and proteins include FMNL2, DNAJB1, DNAJA1, DNAJB1, KPNA3, NAP1L4, DNAJA1, OXNAD1, NAA50, and GPALPP1. The downregulated genes and proteins include DNAJB5, NAT6, SNAPC2, RNASE4, and PMM2. Additionally, aldehyde dehydrogenase 3 family member A1 (ALDH3A1) mRNA downregulated in our study has been reported to decrease in TM from POAG patients (Liu et al., 2013). Aldehyde dehydrogenases play an important role in lipid peroxidation and in the maintenance of redox homeostasis (Choudhary et al., 2005). Interestingly, SLC38A2, a calcium transporter that shows a significant increase in both mRNA and protein and splicing factor, and the suppressor of white-apricot homolog (SFSWAP), which is increased in protein expression analysis, are implicated in systemic hypertension (Iwamoto et al., 2004; Zhang et al., 2010; de Las Fuentes et al., 2013). We found that SLC2A12 and VCAM1 genes decrease significantly under CMS, and, interestingly, SLC2A12 has been shown to influence the POAG risk (Gharahkhani et al., 2021). As SLC2A12 is known to code for a glucose transporter (Wood et al., 2003), further studies on the role of the SLC2A12 and the correlation with metabolic regulations in TM can provide deeper insights into the relationship between hyperglycemia and POAG (Zhou et al., 2014; Hymowitz et al., 2016). The cell adhesion protein VCAM1 has been predicted to be a marker for low flow regions in the TM (Staverosky et al., 2020), thus implicating their significant function in the regulation of AH humor outflow *via* the TM outflow pathway. Thus, further investigations on the role of VCAM1 will help better understand the role of cell adhesion proteins and the pathogenesis of ocular hypertension and POAG.

In general, fluctuations in arterial pressure induce changes in IOP (Bakke et al., 2009). Population-based studies have shown a positive association between blood pressure and IOP (McLeod et al., 1990; Chen and Lai, 2005; Wang et al., 2009). Significant downregulation of the phosphomannomutase 2 (PMM2) level was observed in our proteomic study. PMM2 has been shown by multiple GWAS studies to be a novel locus for POAG (Chen et al., 2014; Wiggs and Pasquale, 2017). PMM2 is known to be involved in glycosylation, enabling posttranslational modifications (addition of N-linked oligosaccharides) on proteins (López-Gálvez et al., 2020). Among the upregulated genes, we found induction of CAVIN4, which is involved in the formation of caveolae (Ogata et al., 2014). Interestingly, genetic polymorphism in caveolin-1 and -2 is associated with POAG (Thorleifsson et al., 2010), and caveolin knockout mice had elevated IOP (Elliott et al., 2016). Additionally, studies have shown oxidative DNA damage to TM cells in patients with POAG (Sacca et al., 2005), implicating oxidative stress as a cause of TM damage in glaucoma (Wu et al., 2022). Finally, the induction of actin cytoskeleton-based contractility and activation of pro-fibrotic pathways in TM have been implicated in glaucoma (Zhavoronkov et al., 2016), and our study identifies mechanical stretch-induced activation of the fibrogenic process, indicating CMS as an appropriate model to study pathological signaling.

## Conclusion

**FIGURE 7:**
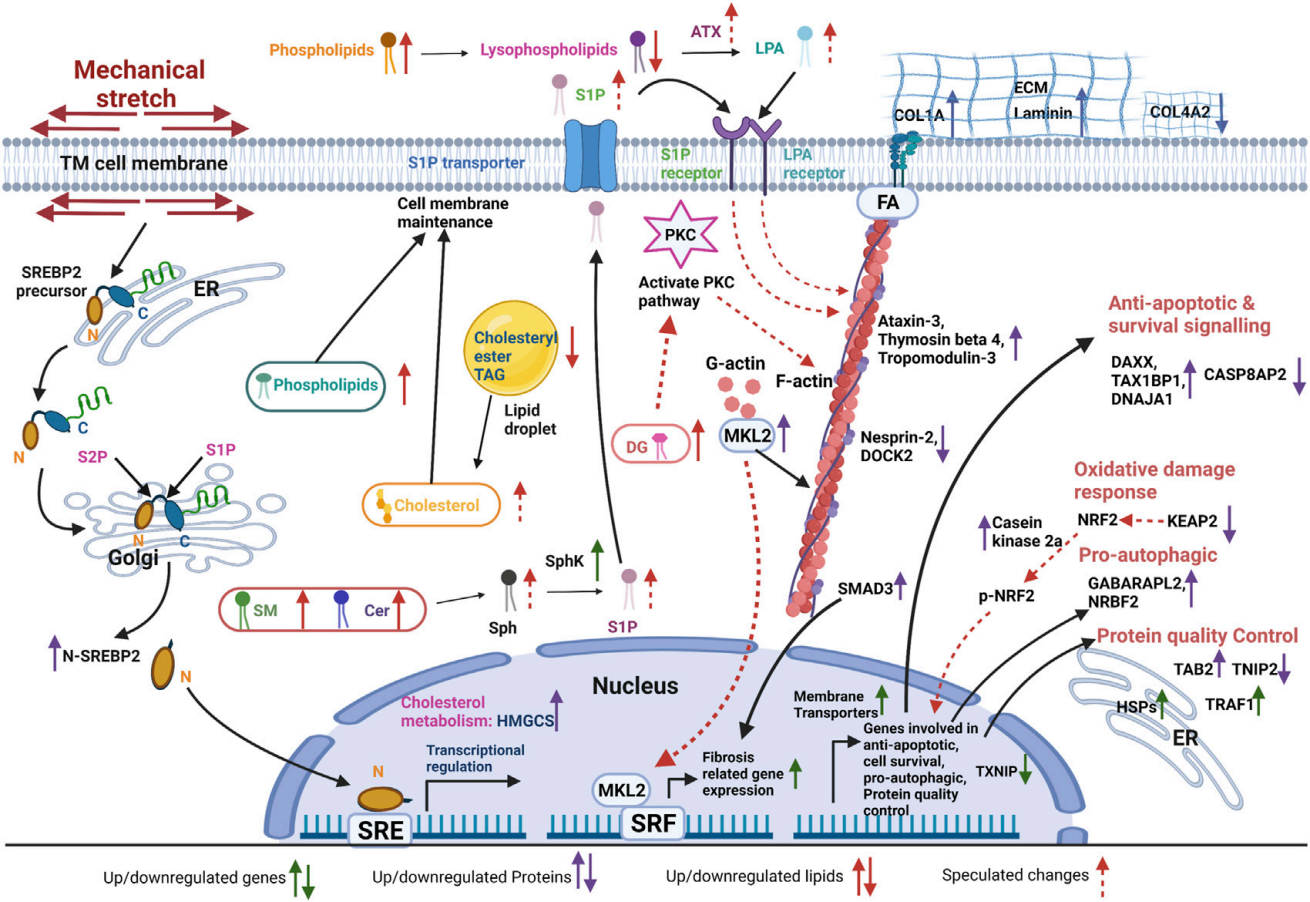
Graphical abstract derived from the multiomics analysis representing the modulation in signaling mechanisms activated and suppressed in TM due to the mechanical (created using BioRender.com).

Based on multiomics analysis of the different pathways activated and suppressed in TM due to mechanical stress (Figure 7), we propose that, in TM, CMS activated cholesterol biosynthesis and lipid messengers contribute to the maintenance of cell membrane fluidity and cell permeability as well as in the regulation of actin-based cell tension and ECM based cell stiffness. Additionally, TM cells turn on the protein quality control machinery, autophagy induction, oxidative damage response, and anti-apoptotic and cell survival signaling to maintain homeostasis against the mechanical insult.

## Data Availability

The datasets presented in this study can be found in online repositories. The names of the repository/repositories and accession number(s) can be found in the article/Supplementary Material.
