# Supplementary material for: Multiomics analysis reveals the mechanical stress-dependent changes in trabecular meshwork cytoskeletal-extracellular matrix interactions
Source: Front Cell Dev Biol. 2022 Sep 13;10:874828. doi: 10.3389/fcell.2022.874828 (PMC9513235; doi:10.3389/fcell.2022.874828)
Supplement: Supplementary file 1 [file Table1.docx]

**SUPPLEMENTARY MATERIAL**

**Supplementary Table 1: List of differentially expressed genes.**

List of all the genes that were differentially expressed in TM subjected to CMS with inclusion criteria of FDR<6% and log2FC ≥ 0.3 for upregulated and ≤ -0.3 for downregulated. Columns (left to right) shows the gene symbol, gene description, fold change, log2FC determined by mRNA sequencing analysis and calculated p value, where p<0.05 was statistically significant.

*# - Upregulated as seen in Youngblood, H.; et al, 2020*

*$ - Downregulated as seen in Youngblood, H.; et al, 2020*

*φ - Upregulated here and downregulated in Youngblood, H.; et al, 2020*

**GENES UPREGULATED:**

| Gene Symbol | Description | Fold change | log2FC | p-value |
| --- | --- | --- | --- | --- |
| MSH4 | mutS homolog 4 | 5.827 | 2.543 | 4.8E-10 |
| SLC8A2 | solute carrier family 8 member A2 | 4.425 | 2.146 | 4.54E-05 |
| AADACP1 | arylacetamide deacetylase pseudogene 1 | 4.347 | 2.120 | 2.12E-06 |
| C9orf152 | chromosome 9 open reading frame 152 | 4.081 | 2.029 | 0.0002 |
| HSPE1-MOB4 | HSPE1-MOB4 readthrough | 3.702 | 1.888 | 2.84E-05 |
| NRIR | negative regulator of interferon response | 3.351 | 1.745 | 3.6E-07 |
| C11orf94 | chromosome 11 open reading frame 94 | 3.059 | 1.613 | 7.6E-05 |
| HIST1H1E^φ^ | N Encodes histone H1.4 protein, one of 11 H1 linker histones | 3.022 | 1.595 | 2.25E-05 |
| ATP6V1C2 | ATPase H+ transporting V1 subunit C2 | 2.955 | 1.563 | 1.06E-04 |
| SLCO4A1 | solute carrier organic anion transporter family member 4A1 | 2.940 | 1.556 | 4.89E-11 |
| SPTBN5 | spectrin beta, non-erythrocytic 5 | 2.637 | 1.399 | 6.09E-06 |
| FBXW10 | F-box and WD repeat domain containing 10 | 2.618 | 1.389 | 2.40E-05 |
| HRK | harakiri, BCL2 interacting protein | 2.609 | 1.383 | 7.08E-04 |
| LSMEM1 | leucine rich single-pass membrane protein 1 | 2.560 | 1.356 | 2E-05 |
| LOC284344 | uncharacterized LOC284344 | 2.497 | 1.320 | 0.00012 |
| SLCO5A1 | solute carrier organic anion transporter family member 5A1 | 2.444 | 1.289 | 8.01E-06 |
| FAM222A | family with sequence similarity 222 member A | 2.424 | 1.277 | 2.99E-04 |
| C9 | complement C9 | 2.387 | 1.255 | 3.28E-04 |
| NPPA-AS1 | NPPA antisense RNA 1 | 2.379 | 1.250 | 2.81E-04 |
| LOC100130705 | Uncharacterized | 2.338 | 1.225 | 3.9E-08 |
| CEP295NL ^#^ | CEP295 N-terminal like | 2.272 | 1.184 | 0.00081 |
| CASP12 | caspase 12 (gene/pseudogene) | 2.191 | 1.132 | 3.93E-04 |
| MTRNR2L8^φ^ | MT-RNR2 like 8 | 2.129 | 1.090 | 1.94E-04 |
| HIST1H3D | N Histone gene cluster-1 (HIST1) encoding H3.1 | 2.089 | 1.063 | 2.00E-04 |
| CCIN | calicin | 2.083 | 1.058 | 1.05E-04 |
| LONRF3 | LON peptidase N-terminal domain and ring finger 3 | 2.070 | 1.049 | 1.3E-05 |
| FZD8 | frizzled class receptor 8 | 2.059 | 1.042 | 1.2E-08 |
| CELF6 | CUGBP Elav-like family member 6 | 2.039 | 1.028 | 4E-07 |
| CAVIN4 | caveolae associated protein 4 | 2.031 | 1.022 | 1.7E-05 |
| KLKB1 | kallikrein B1 | 2.008 | 1.006 | 3.82E-04 |
| GRIP2 | glutamate receptor interacting protein 2 | 1.972 | 0.980 | 1.6E-05 |
| QRICH2 | glutamine rich 2 | 1.944 | 0.959 | 1.56E-04 |
| HES1 | hes family bHLH transcription factor 1 | 1.916 | 0.938 | 9.4E-05 |
| FAM167B | family with sequence similarity 167 member B | 1.903 | 0.928 | 2.25E-04 |
| HSPA1B | heat shock protein family A (Hsp70) member 1B | 1.897 | 0.923 | 2.35E-05 |
| PAX8-AS1 | PAX8 antisense RNA 1 | 1.883 | 0.913 | 6.2E-05 |
| RET | ret proto-oncogene | 1.870 | 0.903 | 3.91E-04 |
| IL11 | interleukin 11 | 1.847 | 0.885 | 6.18E-05 |
| ADCY10P1 | ADCY10 pseudogene 1 | 1.792 | 0.841 | 0.00018 |
| LDLRAD4 | low density lipoprotein receptor class A domain containing 4 | 1.788 | 0.838 | 4.64E-04 |
| GEM | GTP binding protein overexpressed in skeletal muscle | 1.786 | 0.836 | 5.4E-11 |
| PMEPA1 | prostate transmembrane protein, androgen induced 1 | 1.782 | 0.834 | 6.32E-05 |
| RGS17 | regulator of G protein signaling 17 | 1.777 | 0.830 | 5.5E-06 |
| SOX4 | SRY-box transcription factor 4 | 1.777 | 0.829 | 3.1E-10 |
| SPHK1 | sphingosine kinase 1 | 1.775 | 0.828 | 1.03E-06 |
| WHRN | whirlin | 1.761 | 0.817 | 7.35E-09 |
| SLC5A3^#^ | solute carrier family 5 member 3 | 1.755 | 0.811 | 2.95E-07 |
| ZNF503-AS2 | ZNF503 antisense RNA 2 | 1.752 | 0.809 | 1.1E-06 |
| RASEF | RAS and EF-hand domain containing | 1.748 | 0.806 | 2.62E-06 |
| MCM3AP-AS1 | MCM3AP antisense RNA 1 | 1.745 | 0.803 | 2.10E-04 |
| PLEKHA8P1 | pleckstrin homology domain containing A8 pseudogene 1 | 1.743 | 0.802 | 5.69E-05 |
| FAM180A | family with sequence similarity 180 member A | 1.742 | 0.801 | 9.6E-05 |
| MAF | MAF bZIP transcription factor | 1.735 | 0.795 | 5.4E-07 |
| HS3ST3A1 | heparan sulfate-glucosamine 3-sulfotransferase 3A1 | 1.729 | 0.790 | 6.05E-06 |
| RCC1^#^ | regulator of chromosome condensation 1 | 1.716 | 0.779 | 9E-07 |
| HEY1 | hes related family bHLH transcription factor with YRPW motif 1 | 1.714 | 0.777 | 1.60E-06 |
| HERC2P3 | hect domain and RLD 2 pseudogene 3 | 1.710 | 0.774 | 5.2E-07 |
| RSRP1 | arginine and serine rich protein 1 | 1.707 | 0.771 | 4.16E-06 |
| DNAJB9 | DnaJ heat shock protein family (Hsp40) member B9 | 1.699 | 0.765 | 3.65E-09 |
| ITGA2 | integrin subunit alpha 2 | 1.681 | 0.749 | 3.32E-09 |
| NOV | cysteine-rich matricellular protein CCN3 | 1.673 | 0.742 | 8.76E-05 |
| SKIL | SKI like proto-oncogene | 1.664 | 0.735 | 8.43E-06 |
| SGK1 | serum/glucocorticoid regulated kinase 1 | 1.662 | 0.733 | 3.3E-06 |
| MEX3B | mex-3 RNA binding family member B | 1.659 | 0.730 | 1.79E-04 |
| HSPH1 | heat shock protein family H (Hsp110) member 1 | 1.656 | 0.728 | 3.66E-07 |
| SOX11 | SRY-box transcription factor 11 | 1.652 | 0.724 | 7.45E-05 |
| ESM1 | endothelial cell specific molecule 1 | 1.648 | 0.721 | 2.51E-04 |
| NRROS | negative regulator of reactive oxygen species | 1.630 | 0.705 | 8.02E-04 |
| EID3 | EP300 interacting inhibitor of differentiation 3 | 1.629 | 0.704 | 0.00032 |
| KCNJ15 | potassium inwardly rectifying channel subfamily J member 15 | 1.621 | 0.697 | 1.28E-04 |
| JDP2 | Jun dimerization protein 2 | 1.606 | 0.683 | 6.9E-07 |
| MX2 | MX dynamin like GTPase 2 | 1.588 | 0.668 | 6.38E-04 |
| C11orf96 | chromosome 11 open reading frame 96 | 1.588 | 0.667 | 1.33E-05 |
| CHORDC1 | cysteine and histidine rich domain containing 1 | 1.583 | 0.663 | 1.51E-07 |
| XAF1 | XIAP associated factor 1 | 1.581 | 0.661 | 8.17E-07 |
| FZD4 | frizzled class receptor 4 | 1.565 | 0.646 | 6.20E-07 |
| RASD1 ^φ^ | ras related dexamethasone induced 1 | 1.559 | 0.641 | 5.09E-04 |
| F2RL1 | F2R like trypsin receptor 1 | 1.557 | 0.639 | 9.33E-07 |
| TRIB1 | tribbles pseudokinase 1 | 1.553 | 0.635 | 1.21E-04 |
| E2F7 | E2F transcription factor 7 | 1.551 | 0.634 | 2.1E-08 |
| CDC6 | cell division cycle 6 | 1.535 | 0.618 | 5.61E-04 |
| ZNF460 | zinc finger protein 460 | 1.522 | 0.606 | 0.00048 |
| DYRK3 | dual specificity tyrosine phosphorylation regulated kinase 3 | 1.520 | 0.604 | 4.40E-05 |
| RSPO3 | R-spondin 3 | 1.520 | 0.604 | 3.85E-05 |
| RPLP0P2 | ribosomal protein lateral stalk subunit P0 pseudogene 2 | 1.514 | 0.599 | 2.72E-04 |
| HSPA5 | heat shock protein family A (Hsp70) member 5 | 1.511 | 0.596 | 2.12E-04 |
| ITPRIPL2 | ITPRIP like 2 | 1.505 | 0.590 | 3.9E-05 |
| IRF2BP2 | interferon regulatory factor 2 binding protein 2 | 1.503 | 0.588 | 9.7E-06 |
| SLC38A2^#^ | solute carrier family 38 member 2 | 1.502 | 0.586 | 1.98E-05 |
| DHX34 | DExH-box helicase 34 | 1.500 | 0.585 | 3.3E-05 |
| SYT14 | synaptotagmin 14 | 1.499 | 0.584 | 2.09E-07 |
| SLC6A9 | solute carrier family 6 member 9 | 1.498 | 0.583 | 4.35E-04 |
| RNR1 | s-rRNA | 1.484 | 0.570 | 1.03E-05 |
| MEDAG | mesenteric estrogen dependent adipogenesis | 1.479 | 0.565 | 8.31E-04 |
| ZNF326 | zinc finger protein 326 | 1.476 | 0.562 | 1.43E-05 |
| ABCC5 | ATP binding cassette subfamily C member 5 | 1.475 | 0.561 | 0.00028 |
| LOC100379224 | uncharacterized LOC100379224 | 1.472 | 0.558 | 4.65E-04 |
| THAP2 | THAP domain containing 2 | 1.469 | 0.555 | 0.00015 |
| HERPUD1 | homocysteine inducible ER protein with ubiquitin like domain 1 | 1.467 | 0.553 | 3.12E-04 |
| TRAF1 | TNF receptor associated factor 1 | 1.462 | 0.548 | 5.03E-04 |
| ZBTB2 | zinc finger and BTB domain containing 2 | 1.459 | 0.545 | 1.55E-04 |
| DKK1 | dickkopf WNT signaling pathway inhibitor 1 | 1.454 | 0.540 | 2.7E-05 |
| SAMD1 | sterile alpha motif domain containing 1 | 1.448 | 0.534 | 0.00031 |
| HIST1H2BC | Codes for Histone H2B type 1-C/E/F/G/I | 1.446 | 0.532 | 6.44E-04 |
| RPP25 | ribonuclease P and MRP subunit p25 | 1.437 | 0.523 | 0.00036 |
| BHLHE40 | basic helix-loop-helix family member e40 | 1.431 | 0.517 | 5E-05 |
| SPEN | spen family transcriptional repressor | 1.428 | 0.514 | 1.06E-04 |
| RNF144A | ring finger protein 144A | 1.427 | 0.513 | 9.83E-05 |
| SMAD7 | SMAD family member 7 | 1.424 | 0.510 | 0.00013 |
| FRMD5 | FERM domain containing 5 | 1.422 | 0.508 | 8.95E-05 |
| CD3EAP | CD3e molecule associated protein | 1.421 | 0.507 | 6.91E-04 |
| LRRC8C | leucine rich repeat containing 8 VRAC subunit C | 1.420 | 0.506 | 5.93E-05 |
| PM20D2 | peptidase M20 domain containing 2 | 1.417 | 0.503 | 1.93E-04 |
| HIST1H4H | Histone H4 | 1.408 | 0.494 | 0.00082 |
| RBM14 | RNA binding motif protein 14 | 1.408 | 0.493 | 1.62E-05 |
| C3orf52 | chromosome 3 open reading frame 52 | 1.407 | 0.493 | 3.52E-04 |
| PRPF38B | pre-mRNA processing factor 38B | 1.405 | 0.491 | 5.2E-06 |
| SFPQ | splicing factor proline and glutamine rich | 1.398 | 0.484 | 7.2E-05 |
| TMEM233 | transmembrane protein 233 | 1.397 | 0.483 | 0.00014 |
| LUC7L | LUC7 like | 1.397 | 0.482 | 2.91E-05 |
| GPR180 | G protein-coupled receptor 180 | 1.394 | 0.479 | 5.9E-06 |
| LOC646762 | uncharacterized LOC646762 | 1.389 | 0.475 | 0.00033 |
| LURAP1L ^#^ | leucine rich adaptor protein 1 like | 1.382 | 0.467 | 0.00016 |
| MARCKSL1^#^ | MARCKS like 1 | 1.381 | 0.466 | 9.61E-05 |
| TMEM170A | transmembrane protein 170A | 1.378 | 0.462 | 6.2E-05 |
| DDX17 | DEAD-box helicase 17 | 1.374 | 0.458 | 0.0002 |
| MIR100HG | mir-100-let-7a-2-mir-125b-1 cluster host gene | 1.373 | 0.457 | 5.82E-05 |
| DNAJA1 | DnaJ heat shock protein family (Hsp40) member A1 | 1.372 | 0.456 | 1.06E-04 |
| ZBTB1 | zinc finger and BTB domain containing 1 | 1.368 | 0.453 | 5.7E-05 |
| DNAJB1 | DnaJ heat shock protein family (Hsp40) member B1 | 1.362 | 0.446 | 2.01E-05 |
| BANP | BTG3 associated nuclear protein | 1.359 | 0.442 | 4.33E-04 |
| MLKL | mixed lineage kinase domain like pseudokinase | 1.353 | 0.436 | 8.02E-04 |
| KLHL21 | kelch like family member 21 | 1.351 | 0.434 | 0.0008 |
| RALA | RAS like proto-oncogene A | 1.348 | 0.431 | 9.44E-05 |
| HSPB8 | heat shock protein family B (small) member 8 | 1.346 | 0.429 | 0.00041 |
| NXF1 | nuclear RNA export factor 1 | 1.346 | 0.429 | 5.06E-04 |
| HRH1 | histamine receptor H1 | 1.343 | 0.425 | 0.00018 |
| PAXBP1 | PAX3 and PAX7 binding protein 1 | 1.342 | 0.425 | 5.29E-04 |
| SGIP1 | SH3GL interacting endocytic adaptor 1 | 1.334 | 0.416 | 5.68E-04 |
| DNER | delta/notch like EGF repeat containing | 1.318 | 0.398 | 6.70E-04 |
| SLC33A1 | solute carrier family 33 member 1 | 1.315 | 0.395 | 0.0004 |
| SRGAP1 | SLIT-ROBO Rho GTPase activating protein 1 | 1.313 | 0.393 | 0.00077 |
| SRSF6 | serine and arginine rich splicing factor 6 | 1.305 | 0.384 | 3.00E-04 |
| FMNL2 | formin like 2 | 1.303 | 0.382 | 7.31E-04 |
| DUSP10 | dual specificity phosphatase 10 | 1.295 | 0.373 | 5.39E-04 |
| HEXIM1 | HEXIM P-TEFb complex subunit 1 | 1.295 | 0.373 | 6.99E-04 |
| MGA | MAX dimerization protein MGA | 1.291 | 0.368 | 5.27E-04 |
| MMP16 | matrix metallopeptidase 16 | 1.288 | 0.365 | 0.00082 |

**GENES DOWNREGULATED:**

| Gene Symbol | Description | Fold change | log2FC | P-value |
| --- | --- | --- | --- | --- |
| CCDC65 | coiled-coil domain containing 65 | -3.093 | -1.629 | 1.80E-04 |
| GMNC | geminin coiled-coil domain containing | -3.023 | -1.596 | 5.4E-05 |
| OXCT1-AS1 | OXCT1 antisense RNA 1 | -2.876 | -1.524 | 2.02E-04 |
| PLIN4 | perilipin 4 | -2.761 | -1.465 | 0.0003 |
| LINC01006 | long intergenic non-protein coding RNA 1006 | -2.691 | -1.428 | 0.00036 |
| RIPOR2 | RHO family interacting cell polarization regulator 2 | -2.339 | -1.226 | 4.23E-04 |
| HRCT1 | histidine rich carboxyl terminus 1 | -2.163 | -1.113 | 0.00075 |
| LINC00957 | long intergenic non-protein coding RNA 957 | -2.142 | -1.099 | 5.14E-04 |
| ECHDC3 | enoyl-CoA hydratase domain containing 3 | -2.079 | -1.056 | 1.24E-07 |
| ESPNL | espin like | -1.998 | -0.998 | 0.00015 |
| FAHD2CP | fumarylacetoacetate hydrolase domain containing 2C, pseudogene | -1.899 | -0.925 | 1.86E-04 |
| SEMA4G | semaphorin 4G | -1.892 | -0.920 | 2.50E-05 |
| SLC7A14 | solute carrier family 7 member 14 | -1.868 | -0.902 | 5.99E-04 |
| USP27X-AS1 | USP27X antisense RNA 1 (head to head) | -1.845 | -0.883 | 0.0002 |
| SLC16A14 | solute carrier family 16 member 14 | -1.844 | -0.883 | 1.36E-07 |
| HYAL1 | hyaluronidase 1 | -1.798 | -0.847 | 9.25E-05 |
| RAP2C-AS1 | RAP2C antisense RNA 1 | -1.752 | -0.809 | 1.94E-05 |
| KRT19 | keratin 19 | -1.723 | -0.785 | 6.72E-06 |
| SMIM10L2B ^#^ | small integral membrane protein 10 like 2B | -1.676 | -0.745 | 0.00026 |
| CLDN11 | claudin 11 | -1.667 | -0.737 | 7.94E-07 |
| GGACT | gamma-glutamylamine cyclotransferase | -1.663 | -0.734 | 0.00021 |
| DNAJB5 | DnaJ heat shock protein family (Hsp40) member B5 | -1.640 | -0.714 | 2.85E-05 |
| NYNRIN | NYN domain and retroviral integrase containing | -1.568 | -0.649 | 8.45E-05 |
| MRVI1 | murine retrovirus integration site 1 homolog | -1.565 | -0.646 | 3.91E-06 |
| RAB42 | RAB42, member RAS oncogene family | -1.543 | -0.626 | 6.78E-04 |
| CHAF1B | chromatin assembly factor 1 subunit B | -1.539 | -0.622 | 2.43E-04 |
| ACADS | acyl-CoA dehydrogenase short chain | -1.533 | -0.617 | 2.25E-04 |
| TXNRD3 | thioredoxin reductase 3 | -1.518 | -0.602 | 3.27E-05 |
| IDH2 | isocitrate dehydrogenase (NADP(+)) 2 | -1.517 | -0.601 | 2.42E-05 |
| MAP3K6 | mitogen-activated protein kinase kinase kinase 6 | -1.498 | -0.583 | 8.8E-05 |
| NAT6 | Member of the N-acetyltransferase family | -1.497 | -0.582 | 6.78E-04 |
| TXNIP ^$^ | thioredoxin interacting protein | -1.489 | -0.574 | 4.62E-04 |
| ALDH3A1 | aldehyde dehydrogenase 3 family member A1 | -1.473 | -0.559 | 0.00063 |
| ETNK2 | ethanolamine kinase 2 | -1.470 | -0.556 | 7.22E-04 |
| OSCP1 | organic solute carrier partner 1 | -1.447 | -0.533 | 3.38E-04 |
| FLT3LG | fms related tyrosine kinase 3 ligand | -1.446 | -0.532 | 7.85E-04 |
| VCAM1 | vascular cell adhesion molecule 1 | -1.437 | -0.523 | 4.74E-05 |
| GCNT1 | glucosaminyl (N-acetyl) transferase 1 | -1.428 | -0.514 | 1.58E-04 |
| CPT2 | carnitine palmitoyltransferase 2 | -1.423 | -0.509 | 3.25E-05 |
| EFS | embryonal Fyn-associated substrate | -1.423 | -0.508 | 3.43E-04 |
| PLCD1 | phospholipase C delta 1 | -1.413 | -0.498 | 5.14E-05 |
| ALKBH3 | alkB homolog 3, alpha-ketoglutaratedependent dioxygenase | -1.409 | -0.495 | 1.83E-04 |
| ARMCX5 | armadillo repeat containing X-linked 5 | -1.399 | -0.485 | 5.15E-04 |
| CCL2 | C-C motif chemokine ligand 2 | -1.399 | -0.484 | 3.76E-05 |
| CCDC113 | coiled-coil domain containing 113 | -1.391 | -0.477 | 2.59E-04 |
| BBS4 | Bardet-Biedl syndrome 4 | -1.389 | -0.474 | 2.94E-05 |
| BAIAP2-AS1 | heat-to-head antisense lncRNA of BAIAP2 | -1.382 | -0.467 | 6.67E-04 |
| NOTCH2NL | Notch homolog 2 N-terminal-like protein | -1.375 | -0.460 | 0.0007 |
| BTN2A2 | butyrophilin subfamily 2 member A2 | -1.375 | -0.460 | 2.47E-04 |
| EVI5L | ecotropic viral integration site 5 like | -1.369 | -0.453 | 2.83E-04 |
| ALDH3B1 | aldehyde dehydrogenase 3 family member B1 | -1.367 | -0.451 | 1.89E-04 |
| PIK3IP1 | phosphoinositide-3-kinase interacting protein 1 | -1.366 | -0.450 | 1.81E-04 |
| H1FX | Histone H1x | -1.363 | -0.447 | 3.33E-04 |
| CEBPD | CCAAT enhancer binding protein delta | -1.353 | -0.436 | 0.00073 |
| SLC2A12 | solute carrier family 2 member 12 | -1.344 | -0.426 | 0.00031 |
| NMT2 | N-myristoyltransferase 2 | -1.337 | -0.419 | 5.45E-04 |
| DDB2 | damage specific DNA binding protein 2 | -1.335 | -0.417 | 3.20E-04 |
| SNAPC2 | small nuclear RNA activating complex polypeptide 2 | -1.334 | -0.416 | 6.91E-04 |
| ARFGAP2 | ADP ribosylation factor GTPase activating protein 2 | -1.325 | -0.407 | 0.00068 |
| FECH | ferrochelatase | -1.315 | -0.395 | 4.32E-04 |
| CRAT | carnitine O-acetyltransferase | -1.309 | -0.389 | 5.07E-04 |
| MPI | mannose phosphate isomerase | -1.303 | -0.382 | 5.92E-04 |
| PEX11B | peroxisomal biogenesis factor 11 beta | -1.297 | -0.375 | 8.11E-04 |

**Supplementary Table 2- Pathway enrichment analysis for upregulated genes using ShinyGO based on biological process.**

Columns (left to right) show the pathways and genes involved in the given pathway, in TM subjected to CMS, based on biological process using ShinyGO pathway enrichment analysis.

| **Pathway** | **Genes** |
| --- | --- |
| Transmembrane receptor protein serine/threonine kinase signaling pathway | SMAD7 TMEPAI SKIL LDLRAD4 DKK1 SLC33A1 HEL-S-89n HES1 NOV NRROS SOX11 RBM14 |
| Regulation of signal transduction | HERPUD1 TRAF1 IL11 DKK1 TMEPAI SKIL DUSP10 F2RL1 ESM1 MLKL LDLRAD4 FZD4 SMAD7 DNAJA1 SFPQ NOV RSPO3 HEY1 RET SPHK1 HEL-S-89n RGS17 HES1 SLC8A2 SOX4 DYRK3 LURAP1L CAVIN4 NRROS SOX11 MTRNR2L8 ATP6V1C2 TRIB1 HEXIM1 HSPA1B DNAJB9 SLC6A9 SGK1 BANP SRGAP1 |
| Regulation of response to stimulus | HERPUD1 TRAF1 IL11 DKK1 TMEPAI SKIL DUSP10 F2RL1 ESM1 MLKL LDLRAD4 FZD4 SMAD7 DNAJA1 SFPQ ZBTB1 NOV RSPO3 KLKB1 HEY1 RET SPHK1 HEXIM1 RBM14 HEL-S-89n RGS17 C9 HES1 SLC8A2 SOX4 DYRK3 LURAP1L ITGA2 CAVIN4 NRROS SOX11 MTRNR2L8 SRSF6 SLC38A2 ATP6V1C2 TRIB1 HSPA1B DNAJB9 SLC6A9 CHORDC1 SGK1 HSPH1 DNAJB1 HSPB8 BANP SRGAP1 |
| Negative regulation of pathway-restricted SMAD protein phosphorylation | TMEPAI LDLRAD4 SMAD7 DKK1 |
| Ventricular septum morphogenesis | SMAD7 HES1 SOX4 SOX11 HEY1 |
| Regulation of cellular response to stress | HERPUD1 TRAF1 DUSP10 DNAJA1 SFPQ F2RL1 SPHK1 SKIL DYRK3 SLC38A2 HEL-S-89n DNAJB9 CHORDC1 HSPH1 DNAJB1 HSPB8 HSPA1B |
| Negative regulation of transmembrane receptor protein serine/threonine kinase signaling pathway | TMEPAI SKIL LDLRAD4 SMAD7 DKK1 HEL-S-89n NOV NRROS |
| Regulation of transmembrane receptor protein serine/threonine kinase signaling pathway | TMEPAI SKIL LDLRAD4 SMAD7 DKK1 HEL-S-89n HES1 NOV NRROS SOX11 |
| Regulation of cell communication | HERPUD1 TRAF1 IL11 DKK1 TMEPAI SKIL DUSP10 F2RL1 ESM1 MLKL LDLRAD4 FZD4 SMAD7 DNAJA1 SFPQ NOV RSPO3 HEY1 RET SPHK1 HEL-S-89n RGS17 HES1 SLC8A2 SOX4 DYRK3 LURAP1L ITGA2 CAVIN4 NRROS SOX11 HRH1 MTRNR2L8 ATP6V1C2 TRIB1 HEXIM1 HSPA1B DNAJB9 SLC6A9 SGK1 BANP SRGAP1 |
| Negative regulation of nitrogen compound metabolic process | TMEPAI DNAJB1 SKIL DUSP10 H1-4 LDLRAD4 BANP SOX11 HEXIM1 SPEN DNAJA1 SMAD7 DKK1 HES1 SFPQ SRSF6 ZBTB1 BHLHB2 JDP2 HEY1 E2F7 IRF2BP2 SPHK1 ZBTB2 HSPA1B HERPUD1 RASD1 MAF SOX4 QRICH2 DHX34 TRIB1 KLKB1 |
| Regulation of transforming growth factor beta receptor signaling pathway | TMEPAI SKIL LDLRAD4 SMAD7 HEL-S-89n NRROS SOX11 |
| Negative regulation of transforming growth factor beta receptor signaling pathway | TMEPAI SKIL LDLRAD4 SMAD7 HEL-S-89n NRROS |
| Regulation of RNA metabolic process | HES1 SFPQ SOX4 ZBTB1 DNAJB1 BHLHB2 SKIL JDP2 CELF6 ZNF326 HEY1 E2F7 IRF2BP2 H1-4 MGA SOX11 MAF ZBTB2 HEXIM1 ZNF460 PAXBP1 TRAF1 SPEN IL11 DDX17 SMAD7 DKK1 SRSF6 F2RL1 FZD4 RBM14 RASD1 RET CAVIN4 TRIB1 DHX34 BANP SPHK1 HSPA1B SLC38A2 HEL-S-89n CDC6 SGK1 |
| Response to unfolded protein | HEL-S-89n HERPUD1 HSPB8 HSPA1B DNAJB9 DNAJA1 HSPH1 DNAJB1 |
| Negative regulation of transcription by RNA polymerase II | DNAJB1 SKIL SOX11 HEXIM1 SPEN SMAD7 DKK1 HES1 SFPQ ZBTB1 BHLHB2 JDP2 HEY1 E2F7 IRF2BP2 ZBTB2 MAF |

**Supplementary Table 3: List of differentially expressed proteins.**

List of all the proteins that were differentially expressed in TM subjected to CMS with inclusion criteria of FDR≤5% and log2FC ≥0.1 for upregulated and ≤-0.1 for downregulated. Columns (left to right) shows the UniProt accession number, protein description, fold change, log2FC determined by global proteomic analysis and calculated p value, where p<0.05 was statistically significant.

| Accession | Description | Fold change | log2FC | P-value |
| --- | --- | --- | --- | --- |
| Q8N6R0 | Methyltransferase-like protein 13 OS=Homo sapiens GN=METTL13 PE=1 SV=1 | 1.879 | 0.91 | 3.46E-02 |
| Q12872 | Splicing factor, suppressor of white-apricot homolog OS=Homo sapiens GN=SFSWAP PE=1 SV=3 | 1.399 | 0.48 | 3.15E-02 |
| Q9UBU6 | Protein FAM8A1 OS=Homo sapiens GN=FAM8A1 PE=1 SV=1 | 1.324 | 0.41 | 5.04E-02 |
| Q8WXI9 | Transcriptional repressor p66-beta OS=Homo sapiens GN=GATAD2B PE=1 SV=1 | 1.312 | 0.39 | 1.82E-02 |
| Q96QD8 | Sodium-coupled neutral amino acid transporter 2 OS=Homo sapiens GN=SLC38A2 PE=1 SV=2 | 1.308 | 0.39 | 3.78E-02 |
| Q8WVK2 | U4/U6.U5 small nuclear ribonucleoprotein 27 kDa protein OS=Homo sapiens GN=SNRNP27 PE=1 SV=1 | 1.3 | 0.38 | 1.20E-02 |
| Q96F24 | Nuclear receptor-binding factor 2 OS=Homo sapiens GN=NRBF2 PE=1 SV=1 | 1.272 | 0.35 | 6.58E-03 |
| P54252 | Ataxin-3 OS=Homo sapiens GN=ATXN3 PE=1 SV=4 | 1.263 | 0.34 | 1.44E-02 |
| P37268 | Squalene synthase OS=Homo sapiens GN=FDFT1 PE=1 SV=1 | 1.256 | 0.33 | 4.35E-02 |
| Q9ULH7 | MKL/myocardin-like protein 2 OS=Homo sapiens GN=MKL2 PE=1 SV=3 | 1.249 | 0.32 | 2.00E-02 |
| Q8NFC6 | Biorientation of chromosomes in cell division protein 1-like 1 OS=Homo sapiens GN=BOD1L1 PE=1 SV=2 | 1.218 | 0.28 | 2.67E-02 |
| Q6PJT7 | Zinc finger CCCH domain-containing protein 14 OS=Homo sapiens GN=ZC3H14 PE=1 SV=1 | 1.209 | 0.27 | 2.43E-02 |
| Q9NYJ8 | TGF-beta-activated kinase 1 and MAP3K7-binding protein 2 OS=Homo sapiens GN=TAB2 PE=1 SV=1 | 1.208 | 0.27 | 1.52E-02 |
| Q96P47 | Arf-GAP with GTPase, ANK repeat and PH domain-containing protein 3 OS=Homo sapiens GN=AGAP3 PE=1 SV=2 | 1.2 | 0.26 | 1.38E-02 |
| Q9NX62 | Inositol monophosphatase 3 OS=Homo sapiens GN=IMPAD1 PE=1 SV=1 | 1.198 | 0.26 | 1.28E-02 |
| Q9Y561 | Low-density lipoprotein receptor-related protein 12 OS=Homo sapiens GN=LRP12 PE=1 SV=1 | 1.187 | 0.25 | 2.63E-03 |
| Q9H814 | Phosphorylated adapter RNA export protein OS=Homo sapiens GN=PHAX PE=1 SV=1 | 1.184 | 0.24 | 1.41E-02 |
| Q13772 | Nuclear receptor coactivator 4 OS=Homo sapiens GN=NCOA4 PE=1 SV=1 | 1.181 | 0.24 | 4.14E-03 |
| Q15027 | Arf-GAP with coiled-coil, ANK repeat and PH domain-containing protein 1 OS=Homo sapiens GN=ACAP1 PE=1 SV=1 | 1.174 | 0.23 | 3.70E-02 |
| Q15397 | Pumilio homolog 3 OS=Homo sapiens GN=PUM3 PE=1 SV=3 | 1.17 | 0.23 | 3.17E-02 |
| P40189 | Interleukin-6 receptor subunit beta OS=Homo sapiens GN=IL6ST PE=1 SV=2 | 1.167 | 0.22 | 3.68E-02 |
| Q01581 | Hydroxymethylglutaryl-CoA synthase, cytoplasmic OS=Homo sapiens GN=HMGCS1 PE=1 SV=2 | 1.159 | 0.21 | 1.38E-02 |
| P60520 | Gamma-aminobutyric acid receptor-associated protein-like 2 OS=Homo sapiens GN=GABARAPL2 PE=1 SV=1 | 1.157 | 0.21 | 2.60E-02 |
| Q9P0M9 | 39S ribosomal protein L27, mitochondrial OS=Homo sapiens GN=MRPL27 PE=1 SV=1 | 1.156 | 0.21 | 2.38E-02 |
| P47914 | 60S ribosomal protein L29 OS=Homo sapiens GN=RPL29 PE=1 SV=2 | 1.154 | 0.21 | 4.99E-02 |
| Q9NYF8 | Bcl-2-associated transcription factor 1 OS=Homo sapiens GN=BCLAF1 PE=1 SV=2 | 1.152 | 0.2 | 2.56E-02 |
| O00505 | Importin subunit alpha-4 OS=Homo sapiens GN=KPNA3 PE=1 SV=2 | 1.151 | 0.2 | 3.12E-02 |
| Q96PZ0 | Pseudouridylate synthase 7 homolog OS=Homo sapiens GN=PUS7 PE=1 SV=2 | 1.149 | 0.2 | 1.40E-02 |
| Q99805 | Transmembrane 9 superfamily member 2 OS=Homo sapiens GN=TM9SF2 PE=1 SV=1 | 1.148 | 0.2 | 1.84E-02 |
| Q71RC2 | La-related protein 4 OS=Homo sapiens GN=LARP4 PE=1 SV=3 | 1.145 | 0.2 | 4.42E-02 |
| Q99735 | Microsomal glutathione S-transferase 2 OS=Homo sapiens GN=MGST2 PE=1 SV=1 | 1.144 | 0.19 | 1.55E-02 |
| Q9NVS2 | 28S ribosomal protein S18a, mitochondrial OS=Homo sapiens GN=MRPS18A PE=1 SV=1 | 1.143 | 0.19 | 4.97E-02 |
| Q9Y223 | Bifunctional UDP-N-acetylglucosamine 2-epimerase/N-acetylmannosamine kinase OS=Homo sapiens GN=GNE PE=1 SV=1 | 1.139 | 0.19 | 2.96E-02 |
| P47974 | Zinc finger protein 36, C3H1 type-like 2 OS=Homo sapiens GN=ZFP36L2 PE=1 SV=3 | 1.138 | 0.19 | 1.28E-02 |
| P40818 | Ubiquitin carboxyl-terminal hydrolase 8 OS=Homo sapiens GN=USP8 PE=1 SV=1 | 1.134 | 0.18 | 4.11E-02 |
| Q8NHG7 | Small VCP/p97-interacting protein OS=Homo sapiens GN=SVIP PE=1 SV=1 | 1.134 | 0.18 | 1.57E-02 |
| Q96EP5 | DAZ-associated protein 1 OS=Homo sapiens GN=DAZAP1 PE=1 SV=1 | 1.132 | 0.18 | 3.57E-02 |
| Q9NVP1 | ATP-dependent RNA helicase DDX18 OS=Homo sapiens GN=DDX18 PE=1 SV=2 | 1.131 | 0.18 | 4.36E-02 |
| P49756 | RNA-binding protein 25 OS=Homo sapiens GN=RBM25 PE=1 SV=3 | 1.126 | 0.17 | 2.48E-03 |
| Q969G3 | SWI/SNF-related matrix-associated actin-dependent regulator of chromatin subfamily E member 1 OS=Homo sapiens GN=SMARCE1 PE=1 SV=2 | 1.124 | 0.17 | 4.42E-02 |
| P83731 | 60S ribosomal protein L24 OS=Homo sapiens GN=RPL24 PE=1 SV=1 | 1.124 | 0.17 | 1.58E-02 |
| O94923 | D-glucuronyl C5-epimerase OS=Homo sapiens GN=GLCE PE=1 SV=3 | 1.124 | 0.17 | 3.45E-03 |
| P31943 | Heterogeneous nuclear ribonucleoprotein H OS=Homo sapiens GN=HNRNPH1 PE=1 SV=4 | 1.122 | 0.17 | 9.26E-03 |
| Q99733 | Nucleosome assembly protein 1-like 4 OS=Homo sapiens GN=NAP1L4 PE=1 SV=1 | 1.121 | 0.16 | 8.79E-03 |
| P31689 | DnaJ homolog subfamily A member 1 OS=Homo sapiens GN=DNAJA1 PE=1 SV=2 | 1.118 | 0.16 | 1.98E-02 |
| Q10471 | Polypeptide N-acetylgalactosaminyltransferase 2 OS=Homo sapiens GN=GALNT2 PE=1 SV=1 | 1.117 | 0.16 | 2.04E-02 |
| Q96HP4 | Oxidoreductase NAD-binding domain-containing protein 1 OS=Homo sapiens GN=OXNAD1 PE=1 SV=1 | 1.116 | 0.16 | 4.94E-02 |
| O75380 | NADH dehydrogenase [ubiquinone] iron-sulfur protein 6, mitochondrial OS=Homo sapiens GN=NDUFS6 PE=1 SV=1 | 1.113 | 0.15 | 9.20E-03 |
| P42892 | Endothelin-converting enzyme 1 OS=Homo sapiens GN=ECE1 PE=1 SV=2 | 1.11 | 0.15 | 5.07E-02 |
| Q15029 | 116 kDa U5 small nuclear ribonucleoprotein component OS=Homo sapiens GN=EFTUD2 PE=1 SV=1 | 1.11 | 0.15 | 1.29E-02 |
| P18615 | Negative elongation factor E OS=Homo sapiens GN=NELFE PE=1 SV=3 | 1.109 | 0.15 | 4.84E-02 |
| Q9H910 | Hematological and neurological expressed 1-like protein OS=Homo sapiens GN=HN1L PE=1 SV=1 | 1.109 | 0.15 | 2.37E-02 |
| P51148 | Ras-related protein Rab-5C OS=Homo sapiens GN=RAB5C PE=1 SV=2 | 1.108 | 0.15 | 3.67E-02 |
| Q9H2J4 | Phosducin-like protein 3 OS=Homo sapiens GN=PDCL3 PE=1 SV=1 | 1.107 | 0.15 | 2.61E-02 |
| U3KPZ7 | Uncharacterized protein OS=Homo sapiens PE=4 SV=1 | 1.106 | 0.15 | 4.66E-02 |
| Q9NYL9 | Tropomodulin-3 OS=Homo sapiens GN=TMOD3 PE=1 SV=1 | 1.105 | 0.14 | 2.53E-02 |
| O15371 | Eukaryotic translation initiation factor 3 subunit D OS=Homo sapiens GN=EIF3D PE=1 SV=1 | 1.105 | 0.14 | 2.28E-02 |
| Q9UER7 | Death domain-associated protein 6 OS=Homo sapiens GN=DAXX PE=1 SV=2 | 1.099 | 0.14 | 2.68E-03 |
| Q9H0R8 | Gamma-aminobutyric acid receptor-associated protein-like 1 OS=Homo sapiens GN=GABARAPL1 PE=1 SV=1 | 1.098 | 0.13 | 2.60E-02 |
| P08123 | Collagen alpha-2(I) chain OS=Homo sapiens GN=COL1A2 PE=1 SV=7 | 1.098 | 0.14 | 2.51E-02 |
| P62328 | Thymosin beta-4 OS=Homo sapiens GN=TMSB4X PE=1 SV=2 | 1.097 | 0.13 | 4.04E-02 |
| P52597 | Heterogeneous nuclear ribonucleoprotein F OS=Homo sapiens GN=HNRNPF PE=1 SV=3 | 1.097 | 0.13 | 3.01E-02 |
| O43684 | Mitotic checkpoint protein BUB3 OS=Homo sapiens GN=BUB3 PE=1 SV=1 | 1.096 | 0.13 | 3.20E-02 |
| Q9UN86 | Ras GTPase-activating protein-binding protein 2 OS=Homo sapiens GN=G3BP2 PE=1 SV=2 | 1.091 | 0.13 | 3.44E-02 |
| Q9NUL3 | Double-stranded RNA-binding protein Staufen homolog 2 OS=Homo sapiens GN=STAU2 PE=1 SV=1 | 1.091 | 0.13 | 2.47E-02 |
| Q9NQP4 | Prefoldin subunit 4 OS=Homo sapiens GN=PFDN4 PE=1 SV=1 | 1.091 | 0.13 | 2.17E-02 |
| Q15043 | Zinc transporter ZIP14 OS=Homo sapiens GN=SLC39A14 PE=1 SV=3 | 1.088 | 0.12 | 4.16E-02 |
| Q9H9P8 | L-2-hydroxyglutarate dehydrogenase, mitochondrial OS=Homo sapiens GN=L2HGDH PE=1 SV=3 | 1.086 | 0.12 | 4.46E-02 |
| Q8IX12 | Cell division cycle and apoptosis regulator protein 1 OS=Homo sapiens GN=CCAR1 PE=1 SV=2 | 1.086 | 0.12 | 3.48E-02 |
| O15372 | Eukaryotic translation initiation factor 3 subunit H OS=Homo sapiens GN=EIF3H PE=1 SV=1 | 1.085 | 0.12 | 1.68E-02 |
| Q13907 | Isopentenyl-diphosphate Delta-isomerase 1 OS=Homo sapiens GN=IDI1 PE=1 SV=2 | 1.084 | 0.12 | 2.19E-02 |
| Q9BUJ2 | Heterogeneous nuclear ribonucleoprotein U-like protein 1 OS=Homo sapiens GN=HNRNPUL1 PE=1 SV=2 | 1.084 | 0.12 | 1.39E-02 |
| P84022 | Mothers against decapentaplegic homolog 3 OS=Homo sapiens GN=SMAD3 PE=1 SV=1 | 1.083 | 0.11 | 2.57E-02 |
| P46934 | E3 ubiquitin-protein ligase NEDD4 OS=Homo sapiens GN=NEDD4 PE=1 SV=4 | 1.083 | 0.12 | 5.04E-02 |
| Q86VP1 | Tax1-binding protein 1 OS=Homo sapiens GN=TAX1BP1 PE=1 SV=2 | 1.083 | 0.12 | 3.23E-02 |
| O75306 | NADH dehydrogenase [ubiquinone] iron-sulfur protein 2, mitochondrial OS=Homo sapiens GN=NDUFS2 PE=1 SV=2 | 1.082 | 0.11 | 1.95E-02 |
| O43447 | Peptidyl-prolyl cis-trans isomerase H OS=Homo sapiens GN=PPIH PE=1 SV=1 | 1.082 | 0.11 | 1.06E-02 |
| Q9GZZ1 | N-alpha-acetyltransferase 50 OS=Homo sapiens GN=NAA50 PE=1 SV=1 | 1.081 | 0.11 | 6.26E-03 |
| P49840 | Glycogen synthase kinase-3 alpha OS=Homo sapiens GN=GSK3A PE=1 SV=2 | 1.08 | 0.11 | 3.00E-05 |
| Q01650 | Large neutral amino acids transporter small subunit 1 OS=Homo sapiens GN=SLC7A5 PE=1 SV=2 | 1.078 | 0.11 | 4.78E-02 |
| Q9H8W4 | Pleckstrin homology domain-containing family F member 2 OS=Homo sapiens GN=PLEKHF2 PE=1 SV=1 | 1.078 | 0.11 | 2.23E-02 |
| Q9Y657 | Spindlin-1 OS=Homo sapiens GN=SPIN1 PE=1 SV=3 | 1.077 | 0.11 | 4.55E-02 |
| Q13190 | Syntaxin-5 OS=Homo sapiens GN=STX5 PE=1 SV=2 | 1.075 | 0.1 | 3.89E-03 |
| Q92626 | Peroxidasin homolog OS=Homo sapiens GN=PXDN PE=1 SV=2 | 1.074 | 0.1 | 3.21E-02 |
| Q8N3F8 | MICAL-like protein 1 OS=Homo sapiens GN=MICALL1 PE=1 SV=2 | 1.072 | 0.1 | 4.70E-02 |
| Q8IXQ4 | GPALPP motifs-containing protein 1 OS=Homo sapiens GN=GPALPP1 PE=1 SV=1 | 1.072 | 0.1 | 4.56E-02 |
| Q96GY0 | Zinc finger C2HC domain-containing protein 1A OS=Homo sapiens GN=ZC2HC1A PE=1 SV=2 | 1.072 | 0.1 | 2.69E-02 |
| P49821 | NADH dehydrogenase [ubiquinone] flavoprotein 1, mitochondrial OS=Homo sapiens GN=NDUFV1 PE=1 SV=4 | 1.072 | 0.1 | 6.74E-05 |
| O00571 | ATP-dependent RNA helicase DDX3X OS=Homo sapiens GN=DDX3X PE=1 SV=3 | 1.071 | 0.1 | 1.10E-02 |
| P98179 | RNA-binding protein 3 OS=Homo sapiens GN=RBM3 PE=1 SV=1 | 1.07 | 0.1 | 1.08E-02 |
| P35637 | RNA-binding protein FUS OS=Homo sapiens GN=FUS PE=1 SV=1 | 1.069 | 0.1 | 4.73E-02 |
| P19784 | Casein kinase II subunit alpha' OS=Homo sapiens GN=CSNK2A2 PE=1 SV=1 | 1.069 | 0.1 | 3.75E-02 |

**PROTEINS DOWNREGULATED:**

| Accession | Description | Fold change | log2FC | P-value |
| --- | --- | --- | --- | --- |
| Q9H0I3 | Coiled-coil domain-containing protein 113 OS=Homo sapiens GN=CCDC113 PE=1 SV=1 | 0.518 | -0.950 | 8.92E-03 |
| Q8IYT3 | Coiled-coil domain-containing protein 170 OS=Homo sapiens GN=CCDC170 PE=1 SV=3 | 0.53 | -0.910 | 2.77E-02 |
| Q86WH2 | Ras association domain-containing protein 3 OS=Homo sapiens GN=RASSF3 PE=1 SV=1 | 0.556 | -0.850 | 4.16E-02 |
| Q8WXH0 | Nesprin-2 OS=Homo sapiens GN=SYNE2 PE=1 SV=3 | 0.588 | -0.770 | 3.75E-02 |
| Q9UKL3 | CASP8-associated protein 2 OS=Homo sapiens GN=CASP8AP2 PE=1 SV=1 | 0.585 | -0.770 | 3.54E-02 |
| Q92608 | Dedicator of cytokinesis protein 2 OS=Homo sapiens GN=DOCK2 PE=1 SV=2 | 0.59 | -0.760 | 2.64E-02 |
| Q96NW7 | Leucine-rich repeat-containing protein 7 OS=Homo sapiens GN=LRRC7 PE=1 SV=1 | 0.643 | -0.640 | 2.33E-02 |
| Q4G0P3 | Hydrocephalus-inducing protein homolog OS=Homo sapiens GN=HYDIN PE=1 SV=3 | 0.676 | -0.570 | 3.95E-02 |
| Q9Y623 | Myosin-4 OS=Homo sapiens GN=MYH4 PE=1 SV=2 | 0.682 | -0.550 | 1.70E-02 |
| Q9NXR1 | Nuclear distribution protein nudE homolog 1 OS=Homo sapiens GN=NDE1 PE=1 SV=2 | 0.681 | -0.550 | 1.61E-02 |
| Q9Y4C0 | Neurexin-3 OS=Homo sapiens GN=NRXN3 PE=1 SV=4 | 0.687 | -0.540 | 7.61E-03 |
| O43670 | BUB3-interacting and GLEBS motif-containing protein ZNF207 OS=Homo sapiens GN=ZNF207 PE=1 SV=1 | 0.704 | -0.510 | 3.56E-02 |
| Q14145 | Kelch-like ECH-associated protein 1 OS=Homo sapiens GN=KEAP1 PE=1 SV=2 | 0.718 | -0.480 | 4.18E-02 |
| Q9HCK8 | Chromodomain-helicase-DNA-binding protein 8 OS=Homo sapiens GN=CHD8 PE=1 SV=5 | 0.729 | -0.460 | 1.51E-02 |
| P23945 | Follicle-stimulating hormone receptor OS=Homo sapiens GN=FSHR PE=1 SV=3 | 0.747 | -0.420 | 2.23E-02 |
| P53677 | AP-3 complex subunit mu-2 OS=Homo sapiens GN=AP3M2 PE=2 SV=1 | 0.755 | -0.410 | 3.96E-02 |
| O15305 | Phosphomannomutase 2 OS=Homo sapiens GN=PMM2 PE=1 SV=1 | 0.756 | -0.400 | 2.33E-02 |
| O75143 | Autophagy-related protein 13 OS=Homo sapiens GN=ATG13 PE=1 SV=1 | 0.766 | -0.390 | 3.56E-02 |
| P57721 | Poly(rC)-binding protein 3 OS=Homo sapiens GN=PCBP3 PE=2 SV=2 | 0.765 | -0.390 | 7.14E-03 |
| Q14DG7 | Transmembrane protein 132B OS=Homo sapiens GN=TMEM132B PE=2 SV=2 | 0.766 | -0.380 | 3.50E-02 |
| Q5EBM0 | UMP-CMP kinase 2, mitochondrial OS=Homo sapiens GN=CMPK2 PE=1 SV=3 | 0.775 | -0.370 | 1.43E-03 |
| O95155 | Ubiquitin conjugation factor E4 B OS=Homo sapiens GN=UBE4B PE=1 SV=1 | 0.786 | -0.350 | 7.79E-03 |
| P46736 | Lys-63-specific deubiquitinase BRCC36 OS=Homo sapiens GN=BRCC3 PE=1 SV=2 | 0.803 | -0.320 | 1.35E-04 |
| Q9HC36 | rRNA methyltransferase 3, mitochondrial OS=Homo sapiens GN=MRM3 PE=1 SV=2 | 0.802 | -0.320 | 2.86E-02 |
| Q4V328 | GRIP1-associated protein 1 OS=Homo sapiens GN=GRIPAP1 PE=1 SV=1 | 0.8 | -0.320 | 3.56E-02 |
| Q96KR7 | Phosphatase and actin regulator 3 OS=Homo sapiens GN=PHACTR3 PE=1 SV=1 | 0.799 | -0.320 | 3.83E-02 |
| Q4U2R6 | 39S ribosomal protein L51, mitochondrial OS=Homo sapiens GN=MRPL51 PE=1 SV=1 | 0.806 | -0.310 | 4.94E-02 |
| P04217 | Alpha-1B-glycoprotein OS=Homo sapiens GN=A1BG PE=1 SV=4 | 0.812 | -0.300 | 2.01E-02 |
| Q8NFZ5 | TNFAIP3-interacting protein 2 OS=Homo sapiens GN=TNIP2 PE=1 SV=1 | 0.811 | -0.300 | 3.61E-02 |
| P00742 | Coagulation factor X OS=Homo sapiens GN=F10 PE=1 SV=2 | 0.816 | -0.290 | 3.20E-02 |
| Q9NXD2 | Myotubularin-related protein 10 OS=Homo sapiens GN=MTMR10 PE=1 SV=3 | 0.839 | -0.250 | 4.32E-02 |
| Q6P5R6 | 60S ribosomal protein L22-like 1 OS=Homo sapiens GN=RPL22L1 PE=1 SV=2 | 0.855 | -0.230 | 9.86E-03 |
| P34096 | Ribonuclease 4 OS=Homo sapiens GN=RNASE4 PE=1 SV=3 | 0.866 | -0.210 | 3.64E-03 |
| P51888 | Prolargin OS=Homo sapiens GN=PRELP PE=1 SV=1 | 0.864 | -0.210 | 4.61E-02 |
| O14948 | Transcription factor EC OS=Homo sapiens GN=TFEC PE=1 SV=1 | 0.87 | -0.200 | 4.47E-02 |
| O94760 | N(G),N(G)-dimethylarginine dimethylaminohydrolase 1 OS=Homo sapiens GN=DDAH1 PE=1 SV=3 | 0.877 | -0.190 | 2.36E-02 |
| P00761 | Trypsin CONTAMINANT OS=Sus scrofa PE=1 SV=1 | 0.876 | -0.190 | 3.26E-02 |
| P78381 | UDP-galactose translocator OS=Homo sapiens GN=SLC35A2 PE=1 SV=1 | 0.885 | -0.180 | 4.43E-02 |
| P30419 | Glycylpeptide N-tetradecanoyltransferase 1 OS=Homo sapiens GN=NMT1 PE=1 SV=2 | 0.884 | -0.180 | 7.07E-03 |
| P08572 | Collagen alpha-2(IV) chain OS=Homo sapiens GN=COL4A2 PE=1 SV=4 | 0.904 | -0.150 | 1.75E-02 |
| P07741 | Adenine phosphoribosyltransferase OS=Homo sapiens GN=APRT PE=1 SV=2 | 0.907 | -0.140 | 3.83E-02 |
| O14776 | Transcription elongation regulator 1 OS=Homo sapiens GN=TCERG1 PE=1 SV=2 | 0.916 | -0.130 | 7.26E-03 |
| Q13586 | Stromal interaction molecule 1 OS=Homo sapiens GN=STIM1 PE=1 SV=3 | 0.925 | -0.110 | 4.91E-02 |
| Q9BUT1 | 3-hydroxybutyrate dehydrogenase type 2 OS=Homo sapiens GN=BDH2 PE=1 SV=2 | 0.931 | -0.100 | 2.64E-02 |
| Q86SR1 | Polypeptide N-acetylgalactosaminyltransferase 10 OS=Homo sapiens GN=GALNT10 PE=1 SV=2 | 0.930 | -0.100 | 3.36E-02 |
| Q96BX8 | MOB kinase activator 3A OS=Homo sapiens GN=MOB3A PE=1 SV=1 | 0.930 | -0.100 | 1.50E-02 |

**Supplementary Table 4- Pathway enrichment analysis for upregulated proteins using ShinyGO based on biological process.**

Columns (left to right) show the pathways and proteins involved in the given pathway, in TM subjected to CMS, based on biological process using ShinyGO pathway enrichment analysis.

| **Pathway** | **Proteins** |
| --- | --- |
| RNA processing | SFSWAP DAZAP1 DDX18 ZC3H14 RBM3 EFTUD2 RBM25 HNRNPF HNRNPH1 PPIH FUS PUS7 BCLAF1 HNRNPUL1 SNRNP27 DDX3X NELFE SLC38A2 CCAR1 RPL24 RPL29 SMAD3 |
| MRNA processing | SFSWAP DAZAP1 ZC3H14 RBM3 EFTUD2 RBM25 HNRNPF PPIH HNRNPH1 BCLAF1 SNRNP27 NELFE CCAR1 FUS HNRNPUL1 |
| MRNA metabolic process | SFSWAP DAZAP1 ZC3H14 RBM3 EFTUD2 RBM25 HNRNPF PPIH HNRNPH1 FUS PUS7 BCLAF1 SNRNP27 ZFP36L2 NELFE CCAR1 HNRNPUL1 RPL24 RPL29 |
| RNA splicing | SFSWAP DAZAP1 RBM3 EFTUD2 RBM25 HNRNPF HNRNPH1 PPIH FUS BCLAF1 SNRNP27 SLC38A2 CCAR1 HNRNPUL1 |
| RNA splicing, via transesterification reactions and spliceosome | SFSWAP DAZAP1 RBM3 EFTUD2 RBM25 HNRNPF PPIH HNRNPH1 CCAR1 FUS HNRNPUL1 SNRNP27 |
| Regulation of RNA splicing | DAZAP1 RBM3 RBM25 HNRNPF HNRNPH1 FUS SLC38A2 SFSWAP |
| Peptide metabolic process | PUM3 EIF3D RBM3 RPL24 ECE1 RPL29 EIF3H MGST2 PUS7 DDX3X BCLAF1 MRPS18A MRPL27 ZFP36L2 LARP4 GSK3A |
| Macromolecule catabolic process | NEDD4 ZC3H14 CENTG3 SVIP FAM8A1 FUS EIF3H BCLAF1 GSK3A USP8 ZFP36L2 SMAD3 CSNK2A2 GABARAPL2 ATXN3 PDCL3 STX5 PHAX RPL24 BUB3 RPL29 |
| Negative regulation of catabolic process | SVIP GSK3A FUS EIF3H SMAD3 CSNK2A2 GABARAPL2 PDCL3 PHAX TAB2 |
| Negative regulation of cellular catabolic process | SVIP GSK3A FUS EIF3H CSNK2A2 GABARAPL2 PDCL3 PHAX TAB2 |
| Regulation of protein catabolic process | NEDD4 SVIP EIF3H USP8 SMAD3 CSNK2A2 GABARAPL2 ATXN3 GSK3A PDCL3 STX5 |
| Protein-containing complex subunit organization | SFSWAP RPL24 SVIP TMSB4X DAXX NAP1L4 SMARCE1 FUS SMAD3 DDX3X EIF3D TMOD3 EIF3H COL1A2 G3BP2 NDUFS2 GABARAPL2 MRPS18A KPNA3 MRPL27 GABARAPL1 NDUFS6 STX5 NDUFV1 PPIH |
| Cellular macromolecule catabolic process | NEDD4 ZC3H14 CENTG3 SVIP FAM8A1 FUS EIF3H BCLAF1 GSK3A USP8 ZFP36L2 CSNK2A2 GABARAPL2 ATXN3 PDCL3 PHAX RPL24 BUB3 RPL29 |
| Peptide biosynthetic process | PUM3 EIF3D RBM3 RPL24 RPL29 EIF3H MGST2 PUS7 DDX3X BCLAF1 MRPS18A MRPL27 ZFP36L2 LARP4 |
| Cellular amide metabolic process | PUM3 EIF3D RBM3 HMGCS1 RPL24 ECE1 RPL29 EIF3H MGST2 PUS7 DDX3X BCLAF1 MRPS18A MRPL27 ZFP36L2 LARP4 GSK3A GNE |
| Posttranscriptional regulation of gene expression | PUM3 ZC3H14 RBM3 FUS PUS7 LARP4 DDX3X BCLAF1 ZFP36L2 PHAX EIF3D EIF3H SMAD3 |
| Translation | PUM3 EIF3D RBM3 RPL24 RPL29 EIF3H PUS7 DDX3X BCLAF1 MRPS18A MRPL27 ZFP36L2 LARP4 |
| Regulation of catabolic process | CSNK2A2 NEDD4 ZC3H14 SVIP GSK3A FUS EIF3H USP8 ZFP36L2 SMAD3 GABARAPL2 ATXN3 PDCL3 STX5 PHAX TAB2 |
| Negative regulation of macromolecule metabolic process | BOD1L1 SMARCE1 ZC3H14 SVIP NELFE DAXX GATAD2B GSK3A BCLAF1 DNAJA1 FUS PUS7 PFDN4 EIF3H SMAD3 TMSB4X CCAR1 ZFP36L2 MRTFB DDX3X CSNK2A2 GABARAPL2 NEDD4 SLC7A5 PDCL3 PHAX SFSWAP TAX1BP1 RPL24 RPL29 |
| Catabolic process | CSNK2A2 GABARAPL2 NEDD4 ZC3H14 CENTG3 GABARAPL1 NRBF2 SVIP GSK3A FAM8A1 MGST2 FUS ECE1 PXDN EIF3H BCLAF1 IMPAD1 USP8 ZFP36L2 SMAD3 ATXN3 PDCL3 STX5 PHAX TAB2 RPL24 BUB3 RPL29 |
| Negative regulation of metabolic process | BOD1L1 SMARCE1 ZC3H14 SVIP NELFE DAXX GATAD2B GSK3A BCLAF1 DNAJA1 FUS PUS7 PFDN4 EIF3H SMAD3 TMSB4X CCAR1 ZFP36L2 MRTFB DDX3X CSNK2A2 GABARAPL2 NEDD4 SLC7A5 PDCL3 PHAX SFSWAP TAB2 TAX1BP1 RPL24 RPL29 |
| Negative regulation of proteolysis involved in cellular protein catabolic process | SVIP EIF3H CSNK2A2 GABARAPL2 PDCL3 |
| Cellular catabolic process | CSNK2A2 GABARAPL2 NEDD4 ZC3H14 CENTG3 GABARAPL1 NRBF2 SVIP GSK3A FAM8A1 MGST2 FUS ECE1 PXDN EIF3H BCLAF1 USP8 ZFP36L2 ATXN3 PDCL3 PHAX TAB2 RPL24 BUB3 RPL29 |
| Negative regulation of protein catabolic process | SVIP EIF3H SMAD3 CSNK2A2 GABARAPL2 PDCL3 |
| Regulation of cellular amide metabolic process | PUM3 RBM3 PUS7 DDX3X BCLAF1 ZFP36L2 GSK3A LARP4 EIF3D EIF3H |
| Amide biosynthetic process | PUM3 EIF3D RBM3 RPL24 RPL29 EIF3H MGST2 PUS7 DDX3X BCLAF1 MRPS18A MRPL27 ZFP36L2 LARP4 |
| Negative regulation of cellular protein catabolic process | SVIP EIF3H CSNK2A2 GABARAPL2 PDCL3 |
