# Supplementary material for: Multiomics analysis reveals the mechanical stress-dependent changes in trabecular meshwork cytoskeletal-extracellular matrix interactions
Source: Front Cell Dev Biol. 2022 Sep 13;10:874828. doi: 10.3389/fcell.2022.874828 (PMC9513235; doi:10.3389/fcell.2022.874828)
Supplement: Supplementary file 2 [file Image1.pdf]

Supplementary Figure 1- Primary human TM cell characterization with demographics

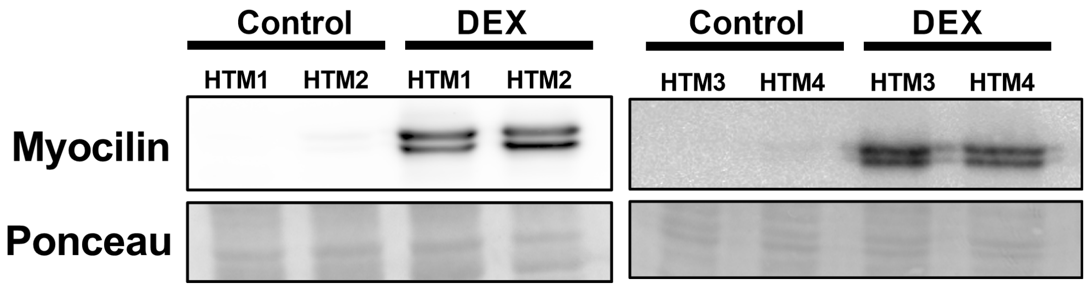

HTM1- 49/ Female/ Caucasian

HTM2- 68/ Male/ Caucasian

HTM3- 68/ Male/ African American

HTM4- 69/ Male/ Caucasian

HTM cell characterization using dexamethasone induced myocilin expression and demographics of the HTM donors.

Supplementary Figure 2- Protein abundance ratio distribution plot

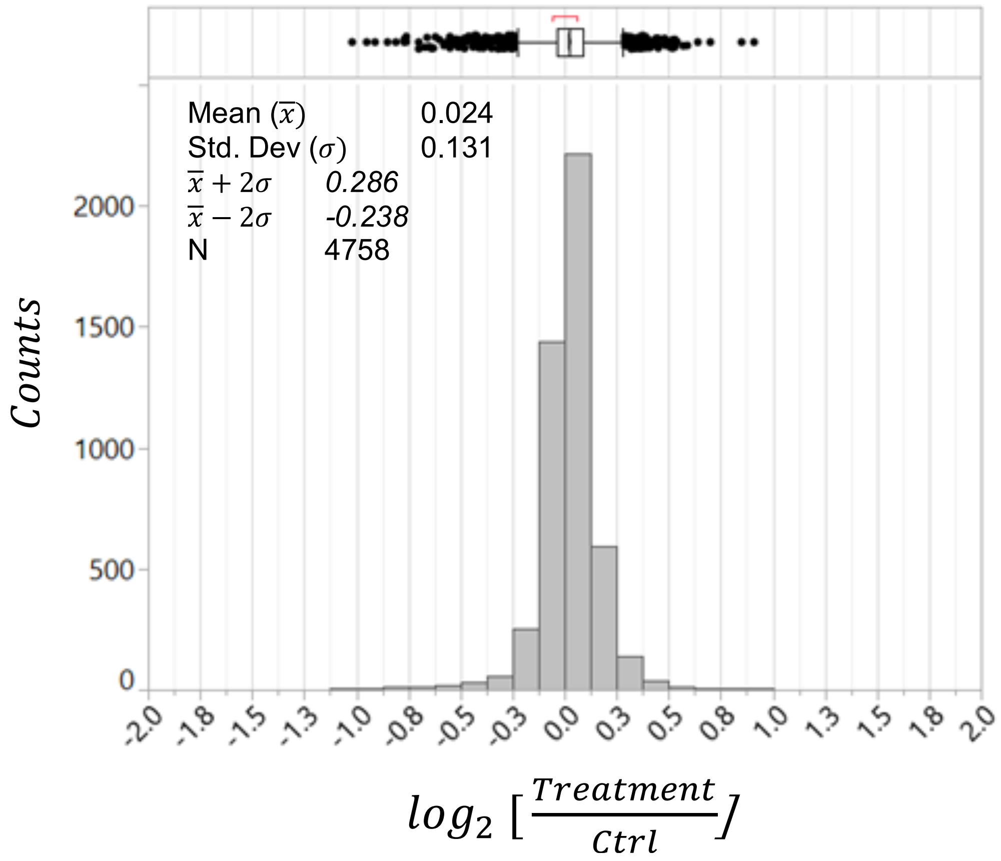

Boxplot and the respective distribution plot of log2 ratio values of protein abundances for "treatment/control" values, indicating the derived mean (x̄), standard deviation (σ) and N—the number of protein abundance ratio values. Also specified are the derived (x̄ ± 2σ) values to indicate the symmetry and the asymmetry of the distribution.

Supplementary Figure 3- STRING network analysis of upregulated genes.

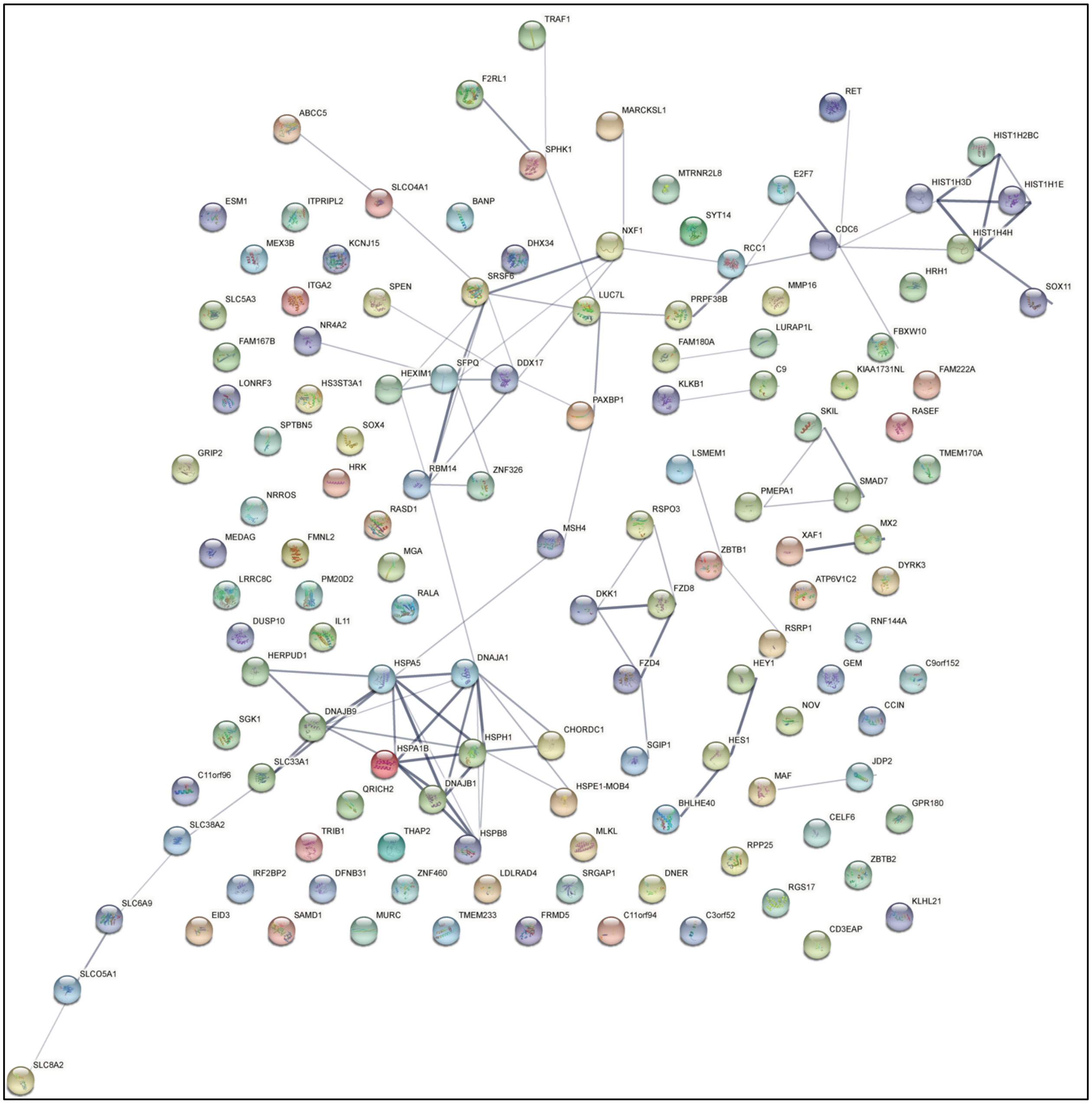

Network analysis of all upregulated genes. Lines drawn between genes indicate at their interconnection.

Supplementary Figure 4- STRING network analysis of downregulated genes.

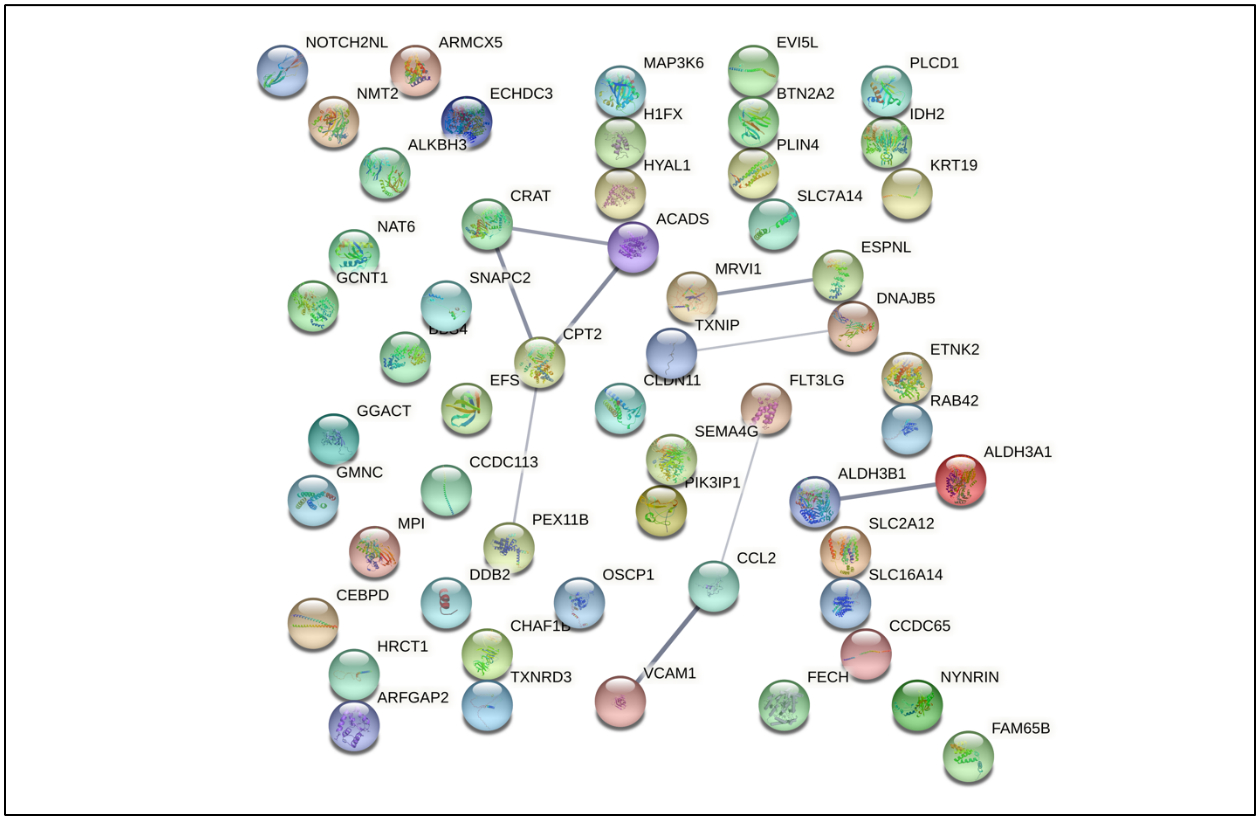

Network analysis of all downregulated genes. Lines drawn between genes indicate at their interconnection.

Supplementary Figure 5- STRING network analysis of upregulated proteins.

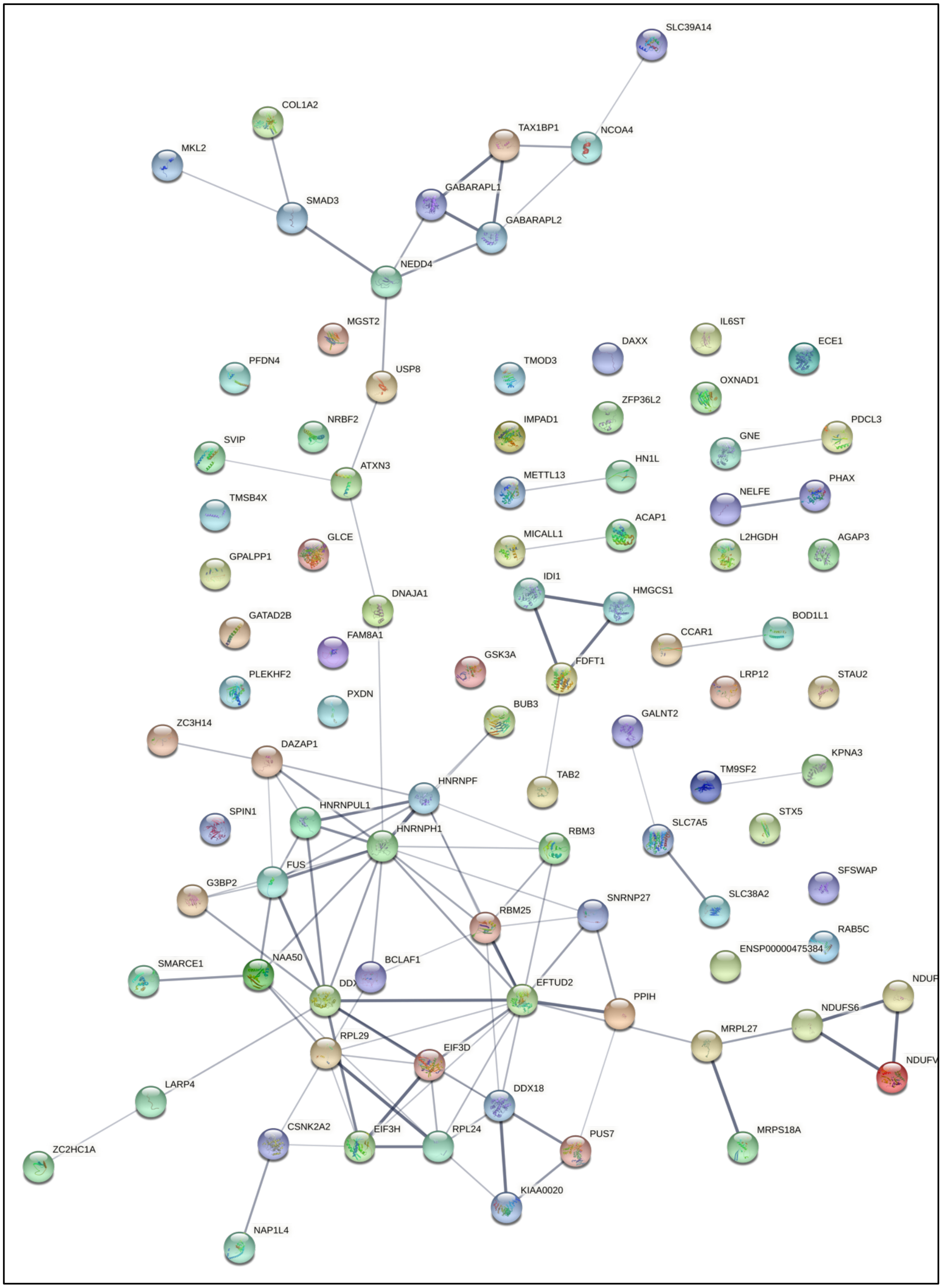

Network analysis of all upregulated proteins. Lines between proteins indicate at their interconnection.

Supplementary Figure 6- STRING network analysis of downregulated proteins.

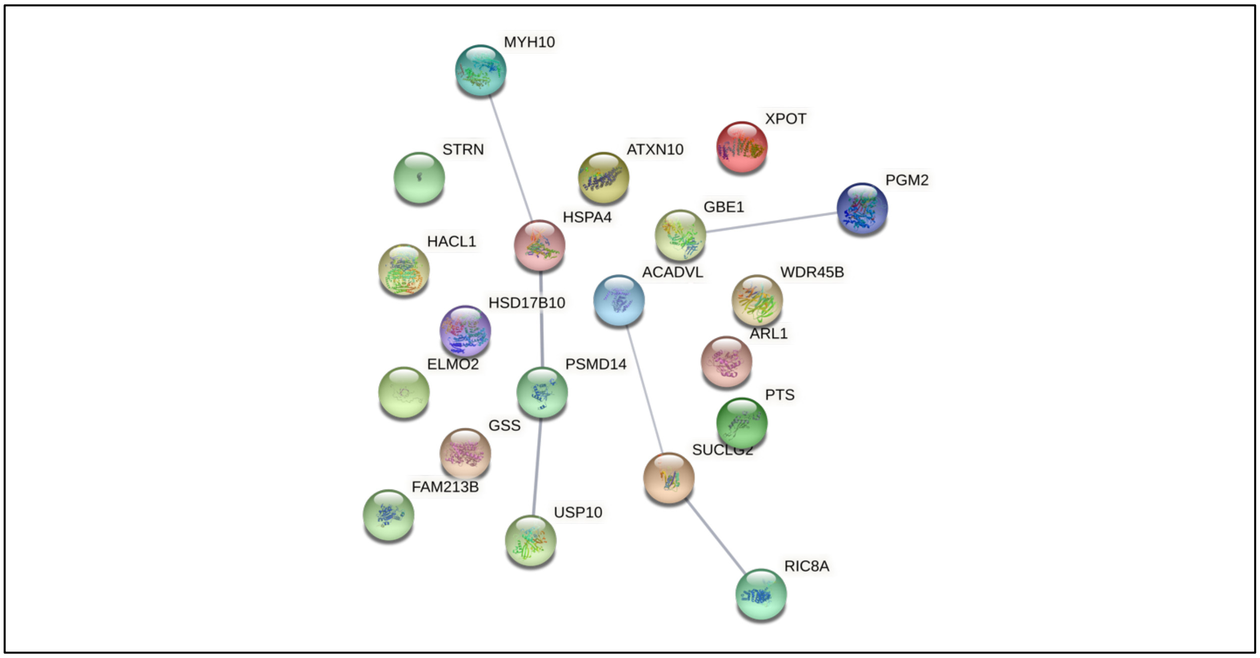

Network analysis of all downregulated proteins. Lines between proteins indicate at their interconnection.

Supplementary Figure 7: Principal component analysis (PCA) of lipid classes.

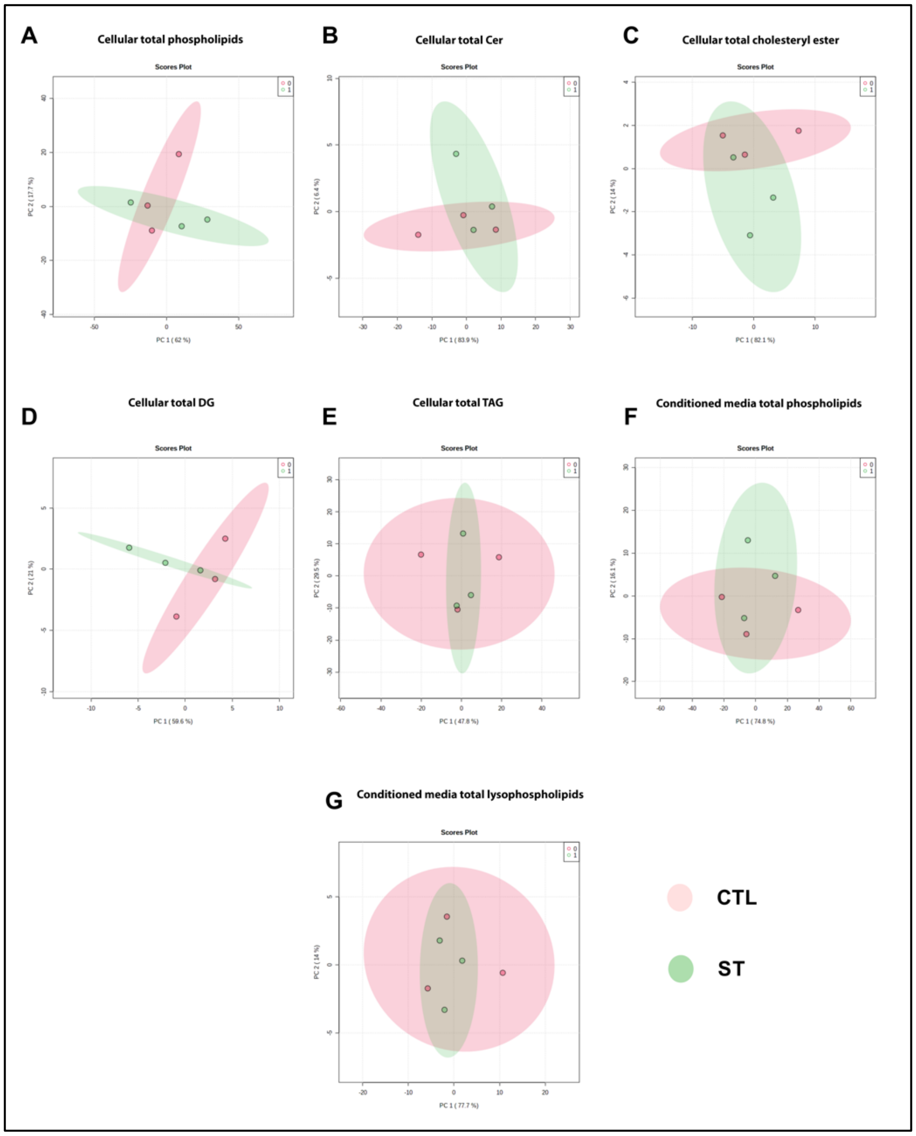

A: Two distinct clusters of cellular total phospholipids in control and stretched HTM cells, and the first two components explained >50% variance, which means cellular total phospholipids were highly different in control (CTL) and stretched (ST) HTM cells. **B:** Two distinct clusters of cellular total ceramide (Cer) in control and stretched HTM cells, and the first two components explained >50% variance, which means cellular Cer was highly different in control and stretched HTM cells. **C:** Two distinct clusters of cellular total cholesteryl ester in control and stretched HTM cells, and the first two components explained >50% variance, which means cellular total cholesteryl ester was highly different in control and stretched HTM cells. **D:** Two distinct clusters of cellular total diacylglycerol (DG) in control and stretched HTM cells, and the first two components explained >50% variance, which means cellular total DG was highly different in control and stretched HTM cells. **E:** There was no distinct cluster of triglycerides (TAG) in control and stretched HTM cells. **F:** Two distinct clusters of media total phospholipids in control and stretched HTM conditioned media, and the first two components explained >50% variance, which means media total phospholipids were highly different in control and stretched HTM conditioned media. **G:** there was no distinct cluster of media total lysophospholipids in control and stretched HTM conditioned media.

Supplementary Figure 8: Comparison of lipid classes between control and stretched HTM cells.

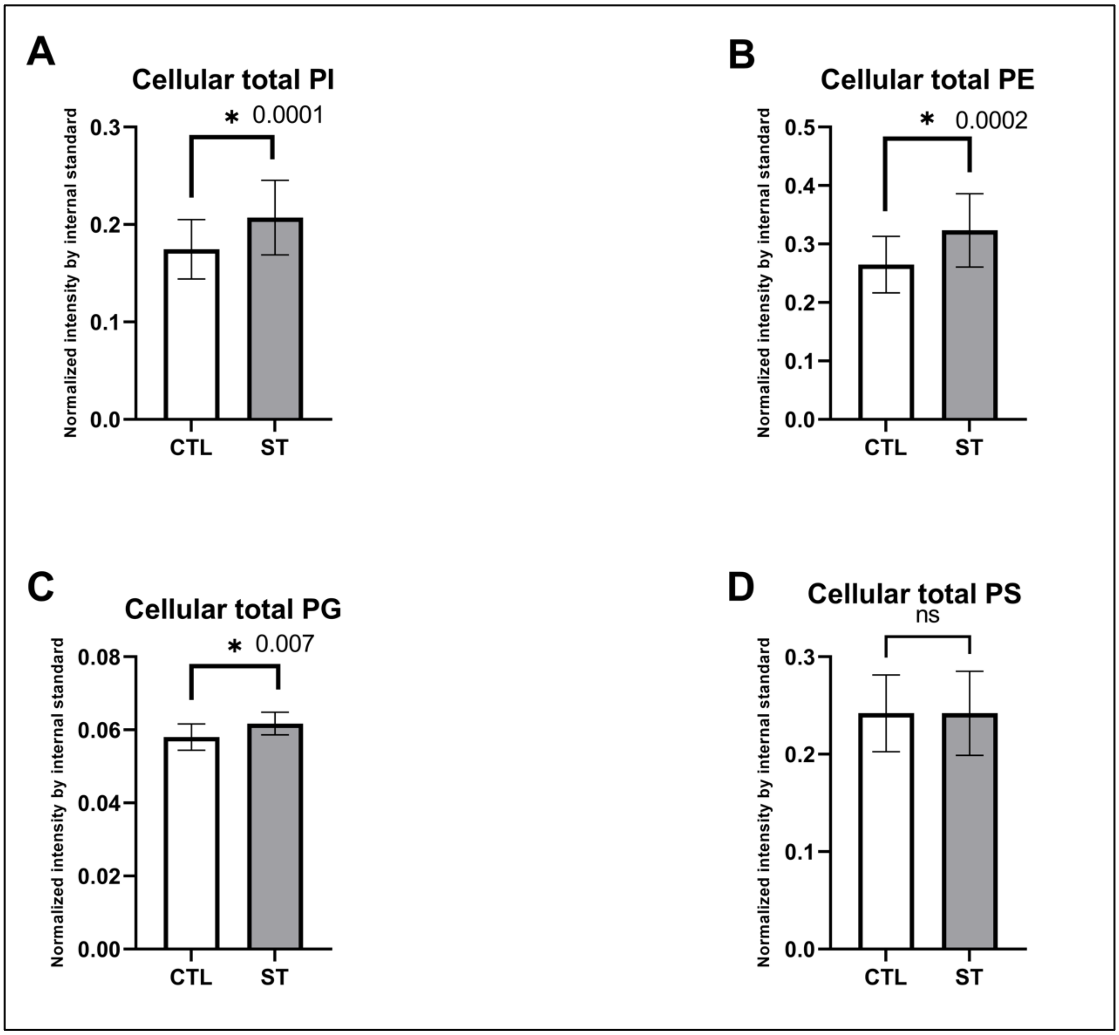

Comparison between control and stretched HTM cells: **A:** Cellular total phosphatidylinositol (PI) was significantly increased in stretched HTM cells. **B:** Cellular total phosphatidylethanolamine (PE) was significantly increased in stretched HTM cells. **C:** Cellular total phosphatidylglycerol (PG) was significantly increased in stretched HTM cells. **D:** No difference of cellular total phosphatidylserine (PS). Values represent the mean ± SEM, where n = 3 (biological replicates). * $p \leq 0.050$ was considered significant.

Supplementary Figure 9: S1P and LPA biosynthesis pathway and downstream effects in IOP regulation.

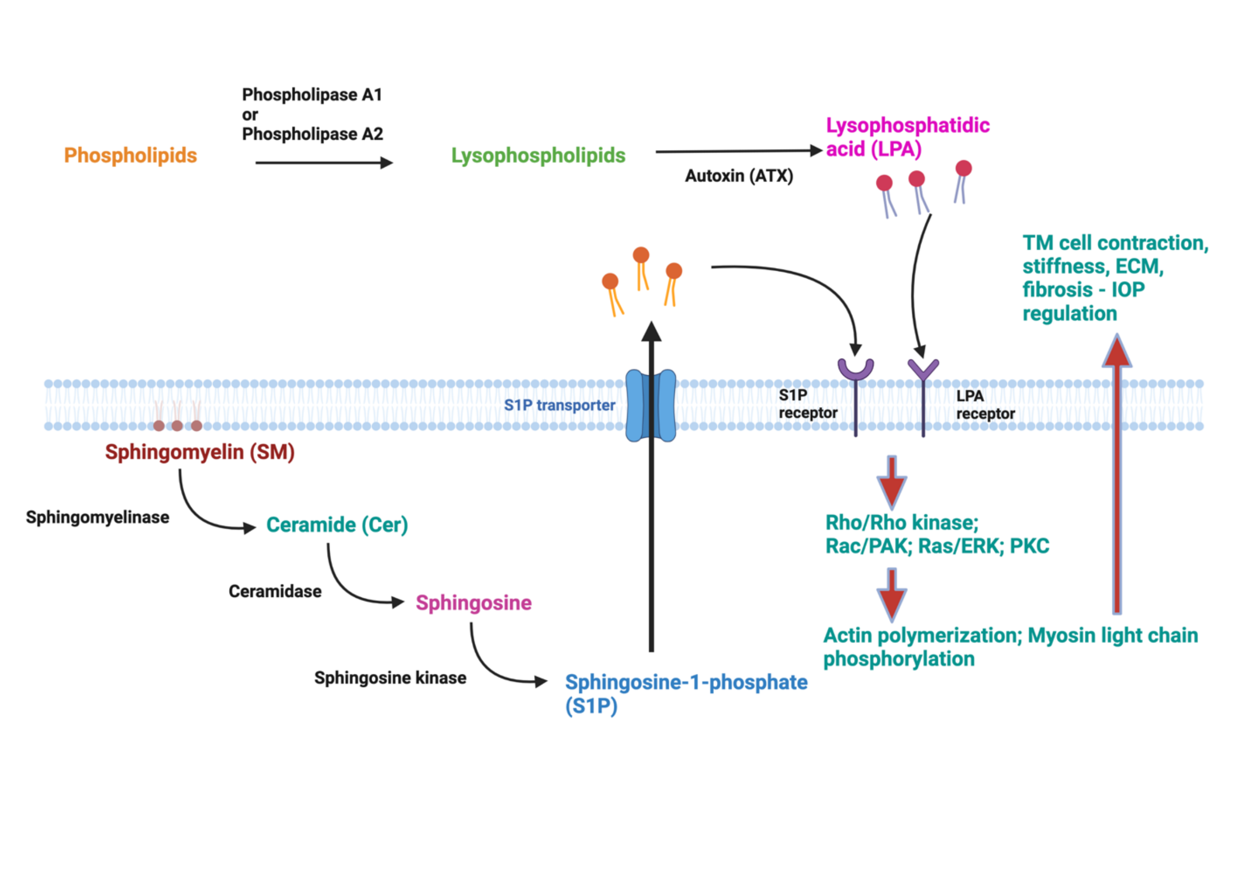

S1P is synthesized in an intracellular environment, then it is exported into the extracellular space, where it binds to its specific receptors located on the cell membrane. LPA is synthesized in the extracellular environment and binds to its specific receptors located on the cell membrane. In HTM cells, both S1P and LPA binding to their cognate receptors lead to the activation of series signaling pathways inside the cell to regulate the actin cytoskeleton, the TM tissue stiffness and IOP (created using BioRender.com)

Supplementary Figure 10: STRING network analysis of upregulated apoptosis associated genes and proteins.

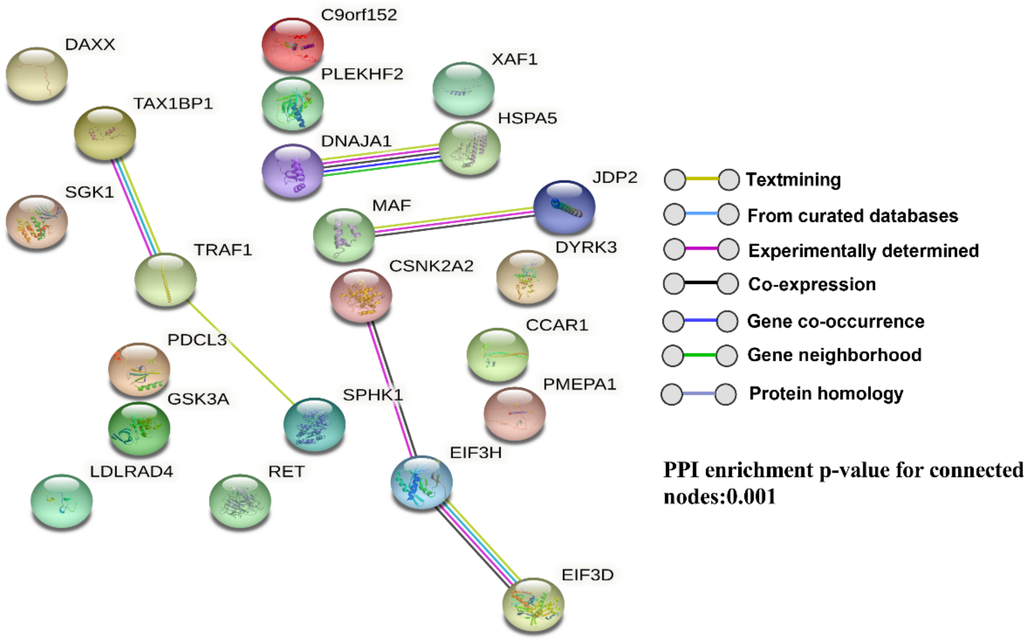

Upregulated apoptosis associated genes	Upregulated apoptosis associated proteins
DNAJA1$	DNAJA1$
TRAF1$	PDCL3$
HSPA5$	DAXX&
DYRK3$	EIF3D$
XAF1&	EIF3H$
JDP2$	TAX1BP1$
SGK1$	CCAR1&
MAF&	GSK3A$&
SPHK1$	PLEKHF2&
PMEPA1&	CSNK2A2$
LDLRAD4&	
RET&	
CASP12&	
C9orf152&	

$ - Anti-apoptotic
&- Pro-apoptotic

Network analysis of all upregulated apoptosis associated genes and proteins. Colored lines between the nodes indicate at their basis for interconnection. Table below gives the list of inclusive genes and proteins. Protein–protein interactions (PPIs) enrichment p-value for connected nodes is considered significant if $p<0.05$. '$' and '&' indicate whether the gene or protein is an anti- or pro-apoptotic respectively.

Supplementary Figure 11- STRING network analysis of downregulated lipid related genes and proteins.

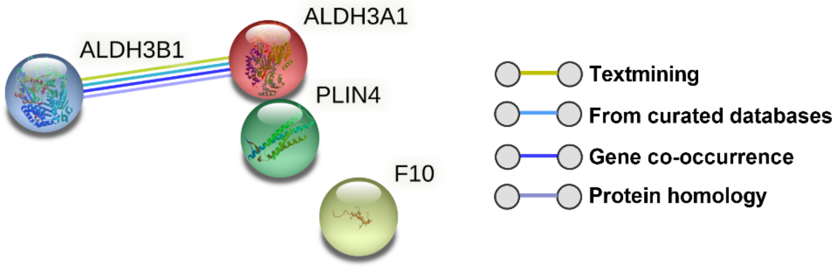

PPI enrichment p-value for connected nodes:0.0039

Downregulated lipid-metabolism and binding genes	Downregulated lipid-metabolism and binding proteins
PLIN4	F10
ALDH3A1	
ALDH3B1	

Network analysis of all downregulated lipid-metabolism and binding related genes and proteins. Colored lines between the nodes indicate at their basis for interconnection. Table below gives the list of inclusive genes and proteins. Protein–protein interactions (PPIs) enrichment p-value for connected nodes is considered significant if $p<0.05$.

Supplementary Figure 12: STRING network analysis of downregulated oxidative stress associated genes and proteins.

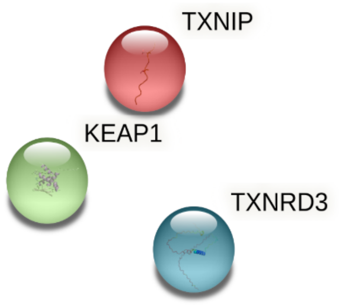

Downregulated oxidative stress associated genes	Downregulated oxidative stress associated proteins
TXNRD3	KEAP1
TXNIP	

Network analysis of all downregulated oxidative stress associated genes and proteins. Table below gives the list of inclusive genes and proteins.

Supplementary Figure 13- STRING network analysis of downregulated apoptosis associated proteins.

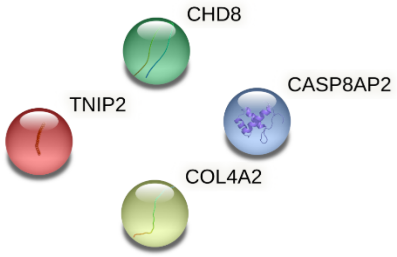

Downregulated apoptosis associated proteins
COL4A2[$]
TNIP2[&]
CHD8[$]
CASP8AP2[&]

$ - Anti-apoptotic
&- Pro-apoptotic

Network analysis of all downregulated apoptosis associated proteins. Table below gives the list of inclusive genes and proteins. '$' and '&' indicates whether the gene or protein is an anti- or pro-apoptotic respectively.

Supplementary Figure 14- STRING network analysis of upregulated autophagy associated proteins.

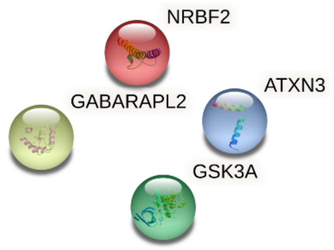

**Upregulated autophagy associated proteins**
NRBF2
ATXN3
GABARAPL2
GSK3A

Network analysis of all upregulated autophagy associated proteins. Table below gives the list of inclusive proteins.

Supplementary Figure 15- STRING network analysis of downregulated autophagy associated proteins.

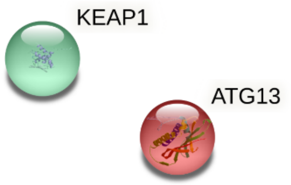

Downregulated autophagy associated proteins
ATG13
KEAP1

Network analysis of all downregulated autophagy associated proteins. Table below gives the list of inclusive proteins.

Supplementary Figure 16- STRING network analysis of upregulated lipid related genes and proteins.
Network analysis of all upregulated lipid-metabolism, binding and homeostasis related genes and proteins. Table below gives the list of inclusive genes and proteins.

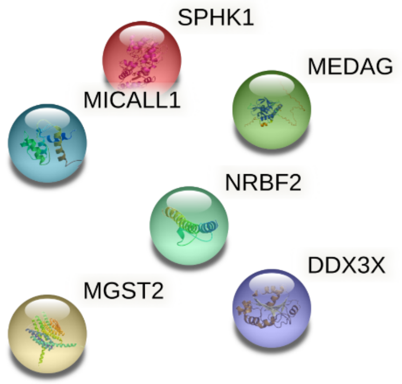

**Upregulated lipid-metabolism, binding, and homeostasis related genes**	**Upregulated lipid-metabolism, binding, and homeostasis related proteins**
MEDAG	NRBF2
SPHK1	MGST2
	DDX3X
	MICALL1
